# Integrated Bioinformatics and Experimental Validation Reveal the Diagnostic and Prognostic Value of SMDT1 in Thyroid Carcinoma

**DOI:** 10.3390/diagnostics16142250

**Published:** 2026-07-18

**Authors:** Tenghong Liu, Hongyi Wu, Zhijun Chen, Wenxin Zhao

**Affiliations:** Department of Thyroid Surgery, Fujian Medical University Union Hospital, Fuzhou 350001, China; tenghong000608@163.com (T.L.); wuhongyi1215@163.com (H.W.); chen_zj1201@163.com (Z.C.)

**Keywords:** *SMDT1*, thyroid cancer, biomarker, prognosis, tumor immune infiltration

## Abstract

**Background**: Thyroid carcinoma (THCA), especially papillary thyroid carcinoma (PTC), remains clinically challenging because recurrence and metastasis occur in a subset of patients. *SMDT1* is an essential regulator of the mitochondrial calcium uniporter complex that may influence tumor progression, but its role in thyroid carcinoma is unclear. This study investigated the expression, clinical significance, and biological functions of *SMDT1* in thyroid carcinoma. **Methods:** Public databases were used to analyze *SMDT1* expression, diagnostic value, prognostic relevance, co-expression networks, functional enrichment, protein interactions, and immune infiltration. *SMDT1* expression was validated in 50 paired PTC and adjacent non-tumorous tissues. In vitro *SMDT1* overexpression was performed in PTC cell lines, followed by quantitative real-time polymerase chain reaction (qRT-PCR), Western blotting (WB), CCK-8, colony formation, wound healing, and transwell assays. **Results:** *SMDT1* was significantly downregulated in thyroid carcinoma tissues, PTC tissues, and PTC cell lines. Low *SMDT1* expression was associated with lymph node metastasis and shorter disease-free survival. Functional analyses linked *SMDT1* with mitochondrial calcium transport, oxidative phosphorylation, apoptosis, cellular senescence, and immune infiltration, including CD8^+^ T cells and activated NK cells. *SMDT1* overexpression significantly suppressed PTC cell proliferation, colony formation, migration, and invasion. **Conclusions:** *SMDT1* may function as a tumor suppressor in thyroid carcinoma and has potential diagnostic and prognostic value. Its effects may involve mitochondrial calcium homeostasis, metabolic regulation, and immune microenvironment remodeling.

## 1. Introduction

Thyroid cancer (THCA) is one of the fastest-increasing malignancies of the endocrine system worldwide, with papillary thyroid carcinoma (PTC) accounting for over 80% of all thyroid cancer cases [1]. Although the majority of PTC patients have favorable outcomes following surgery and adjuvant therapies, a subset of patients experience local recurrence, lymph node metastasis, or even distant metastasis, which substantially impairs long-term quality of life and disease management [2,3]. Current research in the field of thyroid cancer has primarily concentrated on classical driver genes such as BRAF, RAS, and TERT [1]. However, THCA is highly heterogeneous, and existing biomarkers have not fully elucidated the deep metabolic reprogramming that drives tumor progression nor the mechanisms of immune evasion within the tumor microenvironment. Therefore, a comprehensive dissection of the molecular mechanisms underlying THCA initiation and progression and the identification of novel diagnostic and prognostic biomarkers are critical for enabling precise stratification and targeted interventions.

In recent years, an increasing body of evidence indicates that mitochondrial dysfunction, cellular metabolic reprogramming, and dysregulation of the tumor immune microenvironment play pivotal roles in cancer progression [4,5,6,7,8]. The mitochondrial calcium uniporter (MCU) complex and its regulatory components are involved in proliferation, metastasis, and therapeutic resistance in various malignancies, suggesting that the mitochondrial calcium signaling network may represent a novel target for cancer therapy [9,10,11,12]. Single-pass membrane protein with aspartate-rich tail 1 (*SMDT1*), also known as essential MCU regulator (EMRE) or Chromosome 22 open reading frame 32 (*C22orf32*), is an essential regulatory component of the MCU complex that maintains channel stability and enables mitochondrial calcium uptake [13,14,15]. Studies have shown that *SMDT1* mediates mitochondrial calcium homeostasis and thereby influences multiple biological processes, including oxidative phosphorylation, reactive oxygen species production, apoptosis, and cellular senescence [13,16,17,18]. Although accumulating evidence links mitochondrial dysfunction to the initiation and progression of THCA, it remains unclear whether *SMDT1* participates in thyroid cancer progression, what the underlying molecular mechanisms might be, and how it affects the tumor immune microenvironment.

This study systematically investigates the biological role of *SMDT1* in THCA. We performed a pan-cancer expression profile analysis of *SMDT1* using public databases, and subsequently explored *SMDT1*-associated biological processes and potential molecular mechanisms by integrating co-expressed genes, protein–protein interaction networks, and functional enrichment analyses. In addition, immune infiltration analysis was conducted to assess the relationship between *SMDT1* and the tumor immune microenvironment. Finally, clinical PTC tissue samples and in vitro experiments were used to validate *SMDT1* expression patterns and its effects on malignant phenotypes of tumor cells. This study aims to elucidate the function and clinical relevance of *SMDT1* in THCA and to provide new theoretical rationale and molecular targets for early diagnosis, prognostic assessment, and potential targeted therapeutic strategies for THCA.

## 2. Materials and Methods

### 2.1. Pan-Cancer Expression Analysis of SMDT1 and Its Expression in THCA

Pan-cancer expression analysis of *SMDT1* was conducted using the Gene Expression Profiling Interactive Analysis 3 (GEPIA3) database (https://gepia3.bioinfoliu.com/; accessed on 1 March 2026.). *SMDT1* mRNA expression levels in THCA were examined using The Cancer Genome Atlas (TCGA) dataset via the Xiantao Academic platform (https://www.helixlife.cn/; accessed on 1 March 2026.) and datasets (GSE33630 and GSE29265) from the Gene Expression Omnibus (GEO) databases (https://ncbi.nlm.nih.gov/geo/; accessed on 1 March 2026.). The Human Protein Atlas (HPA) (https://www.proteinatlas.org/; accessed on 1 March 2026.) database was employed to assess *SMDT1* protein expression in THCA.

### 2.2. Analysis of SMDT1 Expression Levels and Diagnostic Potential Across Pan-Cancer

Using data from the TCGA database within the Xiantao Academic platform, the diagnostic potential of *SMDT1* expression across pan-cancer was evaluated via receiver operating characteristic (ROC) curve analysis. Two datasets (GSE33630 and GSE29265) from the GEO database were also included in this study for external validation. When the area under the curve (AUC) > 0.5, values of AUC approaching 1 indicate superior diagnostic performance of the variable in predicting outcomes.

### 2.3. Correlation Analysis Between SMDT1 Expression and Clinical Characteristics and Disease-Free Survival (DFS) in THCA Patients

Using the thyroid carcinoma dataset from the TCGA repository accessed via the Xiantao Academic platform, we analyzed differential expression of *SMDT1* across clinical variables, including age, gender, pathologic stage, and pathologic T, N, and M stages. Samples were dichotomized into high- and low-expression groups based on the median *SMDT1* expression. The association between *SMDT1* expression and disease-free survival (DFS) was assessed using the TCGA data on the Home-for-Researchers platform (https://www.home-for-researchers.com/; accessed on 1 March 2026.). For Kaplan–Meier analysis, *p* values were obtained by the log-rank test and hazard ratios (HRs) with 95% confidence intervals (CIs) estimated by univariate Cox regression. The survival package was used to perform proportional hazard hypothesis testing and multivariate Cox regression analysis.

### 2.4. Co-Expression Gene Analysis of SMDT1 in THCA

Using the LinkedOmics database (http://www.linkedomics.org; accessed on 1 March 2026.), co-expressed genes of *SMDT1* (also known as *C22orf32*) in THCA were investigated. The results obtained from the database were analyzed using Spearman correlation coefficients and visualized with heatmaps. The clusterProfiler R package was employed to elucidate and visualize GO (including cellular component (CC), biological process (BP), and molecular function (MF)) and KEGG enrichment analyses of the co-expressed genes. The co-expressed genes were ranked according to the absolute values of Spearman’s correlation coefficients, the top 10 co-expressed genes related to *C22orf32* were selected from all co-expressed genes, and scatterplots depicting the correlations between these 10 co-expressed genes and *C22orf32* were analyzed and visualized using the LinkedOmics database. Additionally, the expression levels of these 10 co-expressed genes in THCA were analyzed via the Xiantao Academic platform.

### 2.5. Co-Expressed Proteins of SMDT1 in THCA

The protein–protein interaction (PPI) network of *SMDT1* co-expressed proteins in THCA was analyzed and constructed using the STRING database (https://cn.string-db.org/; accessed on 1 March 2026.). Concurrently, GO and KEGG enrichment analyses of the co-expressed proteins were performed using the clusterProfiler R package (version 4.5.2).

### 2.6. Immune Infiltrate Analysis of SMDT1 in THCA

Samples were stratified into high-expression and low-expression groups according to the median *SMDT1* expression. Using the TCGA dataset accessible via the Xiantao Academic platform, the CIBERSORT algorithm was applied to assess infiltration differences of 22 immune cell subtypes between *SMDT1* high- and low-expression groups in THCA. Intergroup comparisons were performed using the Wilcoxon rank-sum test, with *p* < 0.05 considered statistically significant.

### 2.7. Clinical Samples

We randomly collected primary PTC tissues and paired non-cancerous tissues from 50 patients who underwent surgery at the Affiliated Union Hospital of Fujian Medical University between January 2026 and March 2026. Tumor and adjacent non-tumor tissues were separated by more than 3 cm, and all specimens were rapidly frozen in liquid nitrogen. All diagnoses were confirmed by histopathological examination, and clinicopathological data were extracted from patient medical records. The clinical characteristics of the PTC patients are detailed in Supplementary Table S1. Individual consent for this analysis was waived due to the retrospective nature. All patients had not received radiotherapy, chemotherapy, or targeted therapy prior to surgery. This study was conducted in accordance with the Declaration of Helsinki and was approved by the Research Ethics Committee of the Affiliated Union Hospital of Fujian Medical University (Approval No. 2026KY586).

### 2.8. Quantitative Real-Time Polymerase Chain Reaction (qRT-PCR)

A detailed experimental protocol was described previously [19]. Briefly, total RNA was extracted from samples using an RNA rapid extraction kit and reverse-transcribed into cDNA with an RNA reverse transcription kit. qRT-PCR was performed on a 7500 Real-Time PCR System. Relative mRNA expression levels of the specified genes were normalized to *β-actin* as the internal control, and each sample was analyzed in triplicate at a minimum. The primer sequences are listed in Supplementary Table S2.

### 2.9. Cell Culture

Normal thyroid follicular epithelial cell line Nthy-ori3-1 and PTC cell lines TPC-1 and BCPAP were obtained from iCell Bioscience Inc (Shanghai, China). All cells were cultured in RPMI 1640 medium (Gibco, Shanghai, China) supplemented with 10% fetal bovine serum (FBS). The cells were maintained in an incubator at 37 °C with 5% CO_2_ (Thermo Fisher Scientific, Waltham, MA, USA).

### 2.10. Western Blotting (WB)

The procedures followed in this study were conducted in accordance with previous studies [20]. In brief, total protein was extracted from samples using RIPA buffer containing protease inhibitors, and protein concentration was determined with a BCA assay kit (Beyotime, Jiangsu, China). Equal amounts of protein were separated by SDS-PAGE and transferred onto PVDF membranes. Membranes were incubated with primary antibodies overnight at 4 °C. After incubation with secondary antibodies the next day, protein bands were detected using a chemiluminescence kit (Beyotime, Jiangsu, China), and band intensities were quantified with ImageJ software (version 2.0). The antibodies used are listed in Supplementary Table S3.

### 2.11. Cell Transfection

To induce overexpression of a specific gene in the cells, PTC cells were seeded in 6-well plates at a density of 5 × 10^4^ cells per well and cultured overnight in complete medium until reaching approximately 70–80% confluence. Transfections were performed using Lipofectamine 3000 (Thermo Fisher Scientific, Waltham, MA, USA) to introduce the overexpression plasmid and a negative control into the cells. Forty-eight hours after transfection, the cells were harvested for further analyses. The *SMDT1* overexpression plasmid used in this study was purchased from GenePharma (Shanghai, China), and the plasmid sequence information is provided in Supplementary Table S4. All transfection procedures followed standardized protocols, and transfection efficiency was assessed by qRT-PCR and WB to ensure reproducibility and accuracy.

### 2.12. Cell Counting Kit-8 (CCK-8) Assay

To assess cell proliferation, transfected PTC cells were seeded into 96-well plates at a density of 2 × 10^3^ cells per well. At 24, 48, 72, and 96 h after seeding, 10 µL of CCK-8 solution (Dojindo) was added to each well and incubated for 4 h, after which absorbance at 450 nm was measured using a multifunctional microplate reader (BioTek Instruments Inc., Winooski, VT, USA).

### 2.13. Colony Formation Assay

A total of 800 cells per well were seeded in 6-well plates and cultured at 37 °C in 5% CO_2_ for 14 days. Thereafter, colonies were fixed with 4% paraformaldehyde and stained with 1% crystal violet. Then, the colonies were photographed and the colony numbers were quantified using ImageJ software (version 2.0). Each experiment was performed in triplicate.

### 2.14. Wound Healing Assay

The cells were plated at 4 × 10^5^ cells per well in 6-well plates and cultured overnight until 80–90% confluence was reached. Scratches in the cells were created using a 200 µL pipette tip, and the cells were maintained in serum-free medium throughout the experiment. Images of the scratched areas were captured at 0 h and 24 h using a microscope.

### 2.15. Transwell Assay

After overnight treatment with serum-free medium, the cell concentration was adjusted to 2 × 10^5^ cells/mL. Two hundred microliters of this cell suspension were seeded into the upper chamber of a 24-well Transwell and incubated overnight. The upper chamber was pre-coated with solidified Matrigel, and 500 µL of complete medium containing 5% FBS was added to the lower chamber. Invading cells were fixed with 4% paraformaldehyde and stained with crystal violet. The number of invading cells was expressed as the mean of five randomly selected microscopic fields. All experiments were performed independently in triplicate.

### 2.16. Statistical Analysis

Data processing and statistical analyses were performed using GraphPad Prism 8.0.2 and R (version 4.5.2). The statistical significance between two groups was assessed using Student's *t*-test or the Wilcoxon rank-sum test, and comparisons between multiple groups were conducted using one-way ANOVA. Each experiment was repeated at least three times, and data are presented as mean ± standard deviation (SD). *p* < 0.05 was considered statistically significant.

## 3. Results

### 3.1. Pan-Cancer Expression Analysis of SMDT1 and Its Differential Expression in THCA

**Figure 1:**
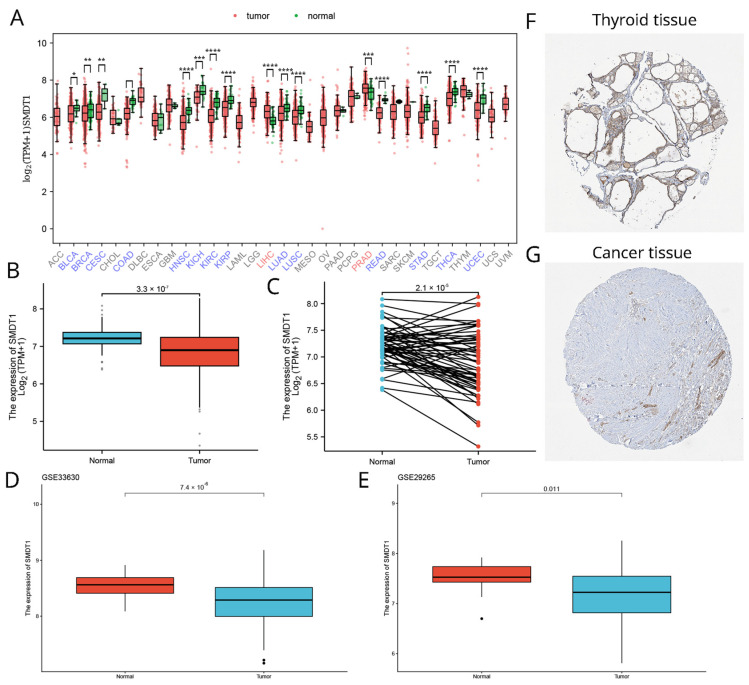
Differential expression of *SMDT1* across various tumors. (**A**) Expression of *SMDT1* in different tumor types. Red text color indicates significantly high expression of SMDT1 in the tumor, while blue text color indicates significantly low expression of SMDT1 in the tumor. (**B**) Differential expression of *SMDT1* mRNA in THCA tissues versus normal thyroid tissues analyzed using the TCGA database. (**C**) Differential expression of *SMDT1* mRNA in THCA tissues and paired adjacent non-tumor tissues analyzed using the TCGA database. (**D**,**E**) Analysis of SMDT1 mRNA expression levels in THCA tissues and normal thyroid tissues based on the GEO database. (**F**,**G**) *SMDT1* protein expression in THCA tissues and normal thyroid tissues from the HPA database. * indicates *p* < 0.05; ** indicates *p* < 0.01; *** indicates *p* < 0.001; **** indicates *p* < 0.0001.

To preliminarily investigate the expression level of *SMDT1*, we performed a pan-cancer expression analysis using the GEPIA3 database. The results indicated that *SMDT1* was downregulated in the majority of cancers, including bladder cancer (BLCA), breast cancer (BRCA), head and neck squamous cell carcinoma (HNSC), kidney chromophobe (KICH), kidney renal clear cell carcinoma (KIRC), kidney renal papillary cell carcinoma (KIRP), lung adenocarcinoma (LUAD), stomach adenocarcinoma (STAD), and thyroid carcinoma (THCA), all reaching statistical significance (*p* < 0.05) (Figure 1A). The full data are provided in Supplementary Table S5.

Further analysis using the TCGA dataset within the Xiantao Academic platform was conducted to validate *SMDT1* mRNA expression in thyroid carcinoma. The results showed that *SMDT1* mRNA levels in THCA tissues were significantly lower than those in normal thyroid tissues (Figure 1B) and in their paired adjacent non-tumorous tissues (Figure 1C). Similar results were also observed in the GSE33630 and GSE29265 datasets (Figure 1D,E). Concurrently, immunohistochemical (IHC) data from the HPA database indicated that *SMDT1* protein expression was markedly downregulated in THCA tissues compared with normal thyroid tissues (Figure 1F,G).

### 3.2. SMDT1 Exhibits Diagnostic Utility Across Multiple Cancer Types

**Figure 2:**
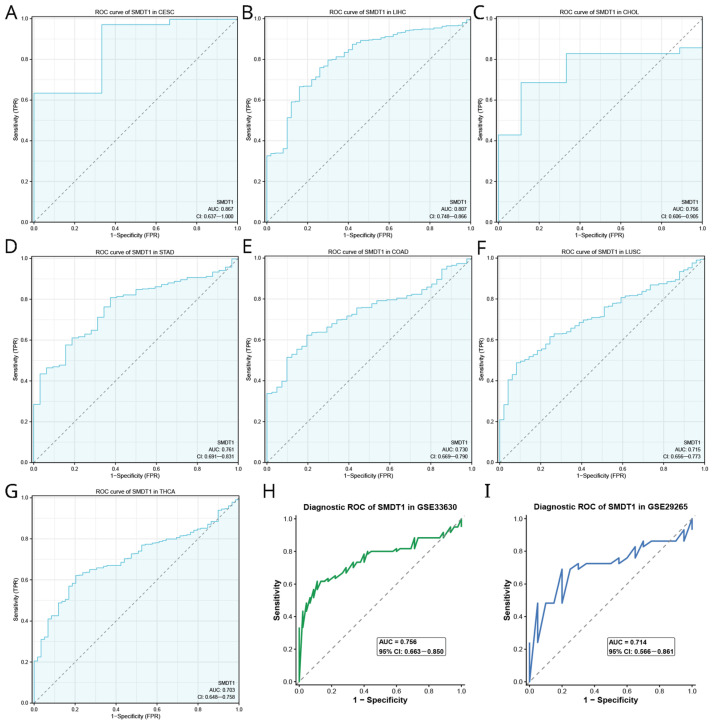
Diagnostic performance of *SMDT1* across multiple cancers. ROC curves of *SMDT1* in (**A**) CESC, (**B**) LIHC, (**C**) CHOL, (**D**) STAD, (**E**) COAD, (**F**) LUSC, and (**G**) THCA. (**H**,**I**) ROC curves were generated to evaluate the diagnostic performance of SMDT1 in THCA based on the GEO database.

ROC curves generated using the Xiantao Academic platform were used to evaluate the diagnostic potential of *SMDT1* across multiple cancers. Figure 2A–G show that *SMDT1* demonstrated biomarker potential in several malignancies, including cervical squamous cell carcinoma and endocervical adenocarcinoma (CESC) with an AUC of 0.867, liver hepatocellular carcinoma (LIHC) with an AUC of 0.807, cholangiocarcinoma (CHOL) with an AUC of 0.756, stomach adenocarcinoma (STAD) with an AUC of 0.761, colon adenocarcinoma (COAD) with an AUC of 0.730, lung squamous cell carcinoma (LUSC) with an AUC of 0.715, and thyroid carcinoma (THCA) with an AUC of 0.703. Additionally, we performed external validation using the GSE33630 and GSE29265 datasets (Figure 2H,I), which similarly confirmed that *SMDT1* exhibits robust diagnostic performance in THCA.

### 3.3. Correlation of SMDT1 Expression with Clinical Features and DFS in THCA Patients

**Figure 3:**
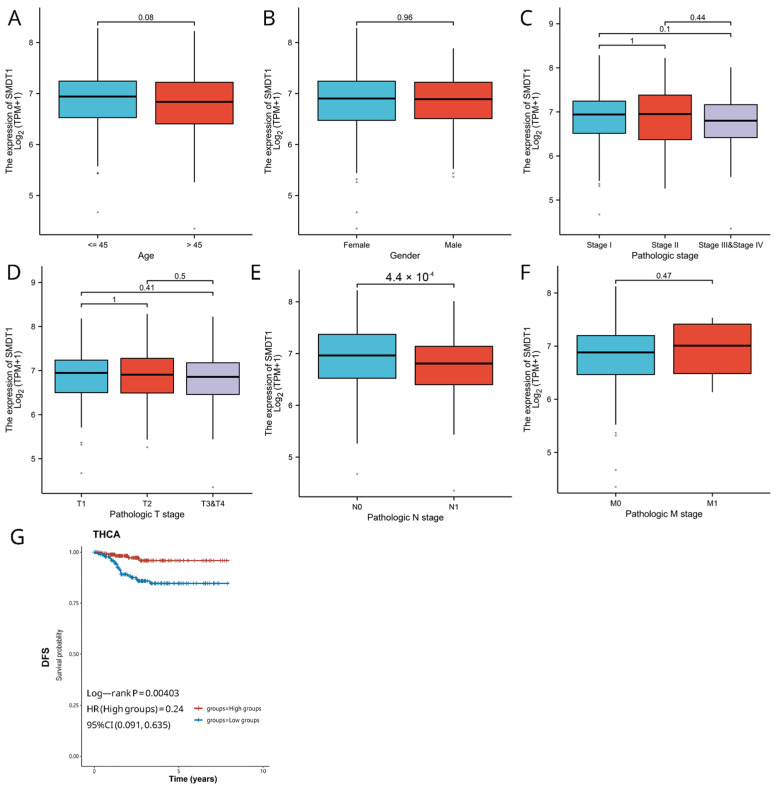
Relationship between *SMDT1* expression and clinical characteristics of THCA patients and DFS analysis. Correlation of *SMDT1* expression with patient (**A**) age, (**B**) gender, (**C**) pathologic stage, (**D**) pathologic T stage, (**E**) pathologic N stage and (**F**) pathologic M stage. (**G**) DFS survival curves for the THCA cohort.

The association between *SMDT1* expression and clinical characteristics of THCA was analyzed using TCGA data via the Xiantao Academic platform. The results showed that *SMDT1* expression was significantly lower in N1 compared with the N0 stage (*p* = 0.00044), indicating a close association between lymph node metastasis status and *SMDT1* expression levels (Figure 3A–F).

Using the TCGA dataset via the Home-for-Researchers platform, patients were dichotomized by the median *SMDT1* expression level. A Kaplan–Meier survival analysis was employed to assess the association between *SMDT1* expression and DFS in THCA patients. In the THCA cohort, patients with high *SMDT1* expression exhibited significantly better DFS compared with those with low expression (*p* < 0.01) (Figure 3G). These findings indicate that high *SMDT1* expression may serve as a protective factor for DFS in thyroid cancer patients, whereas low *SMDT1* expression may be associated with a markedly increased risk of disease recurrence or progression. However, multivariate Cox regression analysis showed that M stage was an independent prognostic factor for DFS in patients with THCA, while high expression of *SMDT1* was not (Supplementary Table S6).

### 3.4. Functional Enrichment and Correlation Analysis of Genes Co-Expressed with SMDT1

**Figure 4:**
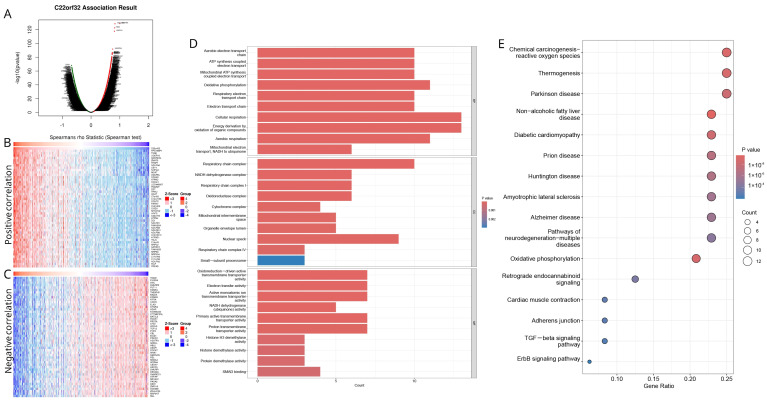
Enrichment analysis of genes co-expressed with *SMDT1* (also known as *C22orf32*) in THCA. (**A**) Volcano plot of *SMDT1* co-expressed genes. Red and green dots represent genes showing significant positive and negative correlation with *SMDT1* expression, respectively. (**B**,**C**) Heatmaps of the top 50 genes positively and negatively correlated with *SMDT1* expression. (**D**) GO enrichment analysis of *SMDT1* co-expressed genes. (**E**) KEGG pathway enrichment analysis of *SMDT1* co-expressed genes.

We performed a genome-wide co-expression analysis of *SMDT1* (also known as *C22orf32*) in THCA using the LinkedOmics database. The volcano plot in Figure 4A depicts the genes as being positively and negatively correlated with *SMDT1* expression. The heatmaps show the top 50 genes as being positively and negatively correlated with *SMDT1* expression (Figure 4B,C), respectively. The gene names are listed in Supplementary Table S7.

Subsequently, GO and KEGG enrichment analyses were performed on all co-expressed genes that were positively and negatively correlated with *SMDT1* expression. GO analysis indicated that *SMDT1* is a protein localized to the mitochondrial inner membrane that is primarily involved in transmembrane transport of mitochondrial calcium, and it also plays a significant role in maintaining mitochondrial architecture (Figure 4D). KEGG analysis revealed that *SMDT1* mainly participates in the calcium signaling pathway, oxidative phosphorylation, and apoptosis pathways (Figure 4E). These findings suggest that *SMDT1* may influence tumor cell apoptosis and senescence by modulating mitochondrial calcium homeostasis and energy metabolism, thereby contributing to thyroid carcinoma pathogenesis.

Additionally, we performed correlation analyses for the top 10 genes co-expressed with *C22orf32*. The results showed that all ten genes exhibited significant positive correlations with *C22orf32* expression in THCA (Supplementary Figure S1). We further evaluated the expression levels of these ten co-expressed genes in THCA tissues versus normal thyroid tissues. Compared with normal thyroid tissues, all ten genes were significantly downregulated in THCA tissues (Supplementary Figure S2), suggesting a co-expression pattern of coordinated downregulation with *SMDT1* in thyroid cancer.

### 3.5. PPI Network and Functional Enrichment Analysis of SMDT1 Co-Expressed Proteins

**Figure 5:**
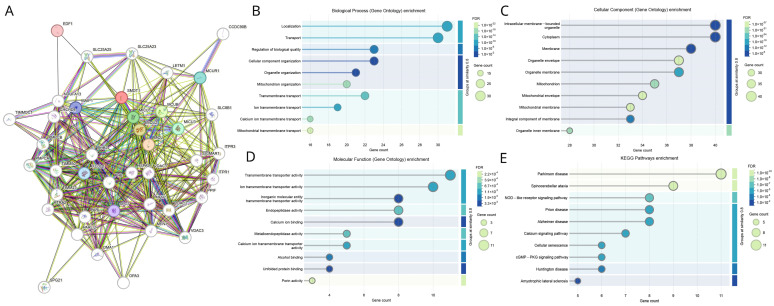
Constructiono f the PPI network and functional enrichment analysis of *SMDT1* co-expressed proteins. (**A**) PPI network of *SMDT1* co-expressed proteins. (**B**–**D**) GO enrichment analysis of *SMDT1* co-expressed proteins, including BP, CC and MF. (**E**) KEGG enrichment analysis of *SMDT1* co-expressed proteins.

We constructed a PPI network of *SMDT1* co-expressed proteins and performed GO and KEGG enrichment analyses based on the STRING database. The PPI network revealed extensive, highly connected interactions between *SMDT1* and key regulators of mitochondrial calcium transport, mitochondrial membrane proteins, and molecules associated with the respiratory chain (Figure 5A). GO analysis indicated that *SMDT1* co-expressed proteins are predominantly localized to mitochondria, the mitochondrial membrane, and organelle inner membranes, and are significantly enriched in mitochondrial organization and transmembrane transport (particularly calcium ion transmembrane transport) and are closely associated with transmembrane transporter activity, ion transmembrane transport activity, and calcium ion binding functions (Figure 5B–D). These findings suggest that *SMDT1* is a mitochondrially localized protein involved in calcium transport across the mitochondrial inner membrane. KEGG analysis showed that *SMDT1* co-expressed proteins are mainly enriched in the calcium signaling pathway, cellular senescence, and NOD-like receptor signaling pathways (Figure 5E).

### 3.6. Correlation Between SMDT1 Expression and Immune Cell Infiltration Levels in THCA

**Figure 6:**
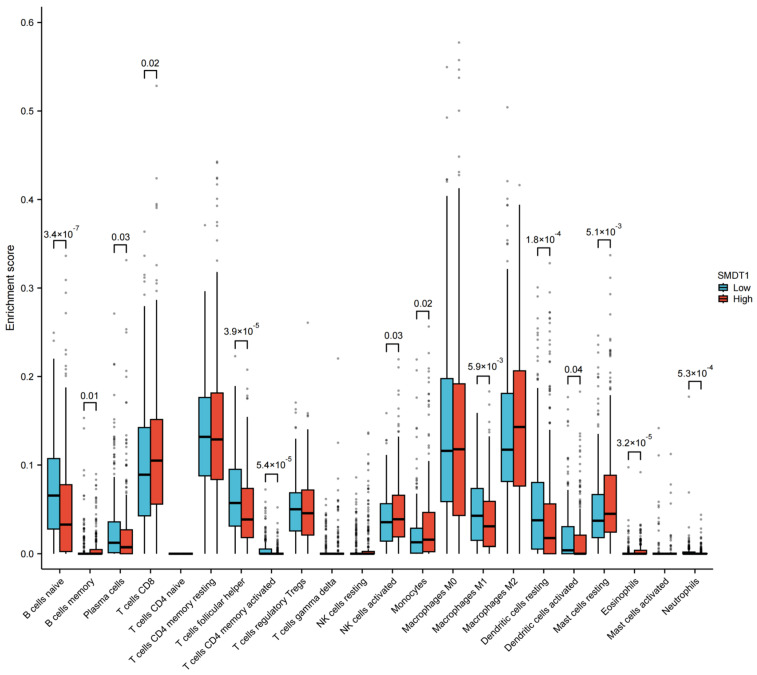
Differential levels of immune cell infiltration in THCA patients with high versus low *SMDT1* expression.

Using the TCGA database accessed via the Xiantao Academic platform, we visualized differential infiltration of 22 immune cell subsets between THCA patients with high versus low *SMDT1* expression (Figure 6). The results showed that the high *SMDT1* expression group exhibited significantly higher infiltration levels of memory B cells (*p* = 0.01), CD8^+^ T cells (*p* = 0.02), activated NK cells (*p* = 0.03), monocytes (*p* = 0.02), resting mast cells (*p* = 0.0051), and eosinophils (*p* < 0.0001). Conversely, the high *SMDT1* expression group had significantly lower infiltration levels of naive B cells (*p* < 0.0001), plasma cells (*p* = 0.03), follicular helper T cells (*p* < 0.0001), M1 macrophages (*p* = 0.0059), resting dendritic cells (*p* = 0.00018), and activated dendritic cells (*p* = 0.04). Notably, activated CD8^+^ T cells and NK cells are key effector immune cells responsible for tumor cell clearance; their increased infiltration suggests that high *SMDT1* expression may suppress THCA progression by enhancing antitumor immunity and improving the immune microenvironment.

### 3.7. Expression of SMDT1 in PTC Tissues and Cells

**Figure 7:**
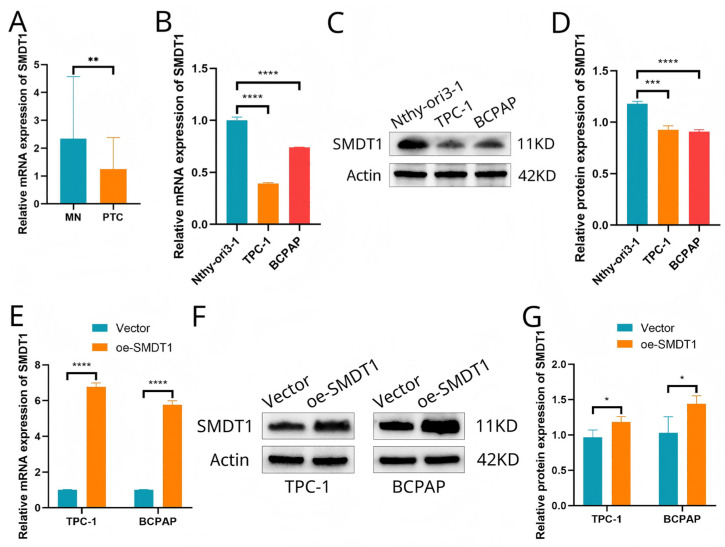
Differential expression of *SMDT1* in PTC tissues and cells. (**A**) *SMDT1* mRNA expression levels in PTC and matched normal (MN) tissues were measured by qRT-PCR. (**B**–**D**) Differential expression of *SMDT1* mRNA and protein in a normal thyroid follicular epithelial cell line and PTC cell lines was assessed by qRT-PCR and WB. (**E**–**G**) Transfection efficiency of the *SMDT1* overexpression plasmid was evaluated by qRT-PCR and WB. * indicates *p* < 0.05; ** indicates *p* < 0.01; *** indicates *p* < 0.001; and **** indicates *p* < 0.0001.

To investigate *SMDT1* expression in PTC, we performed qRT-PCR to assess differential *SMDT1* mRNA levels in 50 primary PTC specimens and their paired matched normal (MN) tissues. The results demonstrated that *SMDT1* mRNA expression was significantly reduced in PTC tissues compared with MN tissues (Figure 7A). In addition, qRT-PCR and WB analyses were used to evaluate *SMDT1* mRNA and protein expression in a normal thyroid follicular epithelial cell line (Nthy-ori3-1) and PTC cell lines (TPC-1 and BCPAP). These assays revealed markedly downregulated *SMDT1* expression in the PTC cell lines (Figure 7B–D). Collectively, these findings indicate that *SMDT1* is significantly underexpressed in PTC tissues and cells.

Subsequently, we transfected TPC-1 and BCPAP cells with an overexpression plasmid to establish control cell lines (Vector) and *SMDT1* overexpression cell lines (oe-*SMDT1*) and assessed transfection efficiency by qRT-PCR and WB. The results showed that, compared with the control group, mRNA and protein expression levels of *SMDT1* were significantly increased in the *SMDT1* overexpression group (Figure 7E–G).

### 3.8. Overexpression of SMDT1 Inhibits the Proliferation, Migration, and Invasion Capacities of PTC Cells

**Figure 8:**
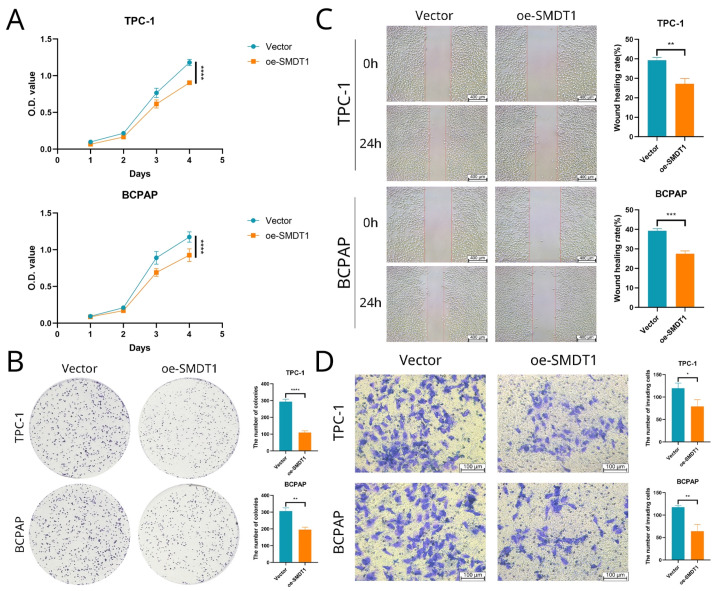
Inhibitory effect of *SMDT1* on the malignant phenotype of PTC cells. (**A**) Effect of *SMDT1* on cell proliferation assessed by CCK-8 assay. (**B**) Effect of *SMDT1* on colony-forming ability assessed by colony formation assay. (**C**) Effect of *SMDT1* on cell migration assessed by wound healing assay. (**D**) Effect of *SMDT1* on cell invasion assessed by Transwell assay. * indicates *p* < 0.05; ** indicates *p* < 0.01; *** indicates *p* < 0.001; and **** indicates *p* < 0.0001.

The CCK-8 assay results showed that *SMDT1* overexpression significantly inhibited cell proliferation compared with the control group (Figure 8A). Colony formation assays further confirmed the growth-suppressive effect of *SMDT1* on PTC cells: the number of colonies formed by cells overexpressing *SMDT1* was markedly reduced relative to the controls (Figure 8B). In addition, wound healing and Transwell invasion assays were performed to assess the effects of *SMDT1* overexpression on PTC cell migration and invasion. The wound healing assay demonstrated a significant reduction in cell migration rate following *SMDT1* overexpression compared with the control group (Figure 8C). Consistently, the Transwell invasion assay showed a significant decrease in the number of cells traversing the membrane upon *SMDT1* overexpression (Figure 8D). Collectively, these results indicate that *SMDT1* overexpression markedly suppresses the malignant phenotype of PTC cells and exerts a tumor-suppressive role in PTC progression.

## 4. Discussion

In this study, by integrating bioinformatic analyses, validation in clinical specimens, and in vitro functional assays, we demonstrate that *SMDT1* is markedly downregulated in THCA and that its low expression is associated with lymph node metastasis and poorer recurrence-free survival. The results from functional enrichment and immune infiltration analyses indicate that *SMDT1* may be involved in regulating mitochondrial calcium signaling, oxidative phosphorylation, apoptosis-related pathways, and remodeling of the tumor immune microenvironment. Moreover, *SMDT1* overexpression significantly suppressed proliferation, migration, and invasion of PTC cells, further supporting a tumor-suppressive role for *SMDT1* in THCA progression.

Mitochondrial calcium homeostasis has emerged as a central regulator of cancer cell metabolism, survival, and metastatic behavior. Increasing evidence in recent years indicates that the MCU complex serves not only as the principal channel for mitochondrial calcium uptake but also as a critical nexus linking metabolic reprogramming to tumor progression [9,11,18]. Several reviews have systematically described the roles of individual MCU complex components in tumorigenesis, among which *SMDT1* is regarded as an indispensable regulatory subunit for maintaining MCU complex stability and calcium-conducting capacity [21,22]. D’Angelo et al. reported that loss of *SMDT1* leads to functional inactivation of the MCU complex, thereby affecting the activity of key tricarboxylic acid cycle enzymes, adenosine triphosphate (ATP) production, and redox homeostasis [18,23]. Our study found that *SMDT1* is significantly downregulated in THCA, a finding that is highly consistent with the view that mitochondrial calcium imbalance promotes adaptive tumor evolution [24]. At the same time, the role of the MCU signaling axis exhibits clear tissue specificity across different cancers. For example, MCU overexpression promotes proliferation and metastasis in pancreatic cancer [25], whereas in some metabolism-dependent tumors, insufficient mitochondrial calcium uptake induces metabolic remodeling and enhances malignant phenotypes [18,26]. This dual effect suggests that different components of the MCU complex may perform distinct biological functions depending on the tumor context. Here, we demonstrate for the first time that *SMDT1* is more likely to act as a tumor suppressor in THCA, thereby enriching the functional repertoire of the MCU signaling network in cancer.

Moreover, our analyses revealed that low *SMDT1* expression is significantly associated with lymph node metastasis and shorter DFS. Lymph node metastasis is a major determinant of recurrence risk in THCA patients and reflects the acquisition of invasive biological traits by the tumor. The inverse correlation between *SMDT1* expression and lymph node metastasis suggests that loss of *SMDT1* may facilitate tumor metastatic progression. This hypothesis is preliminarily supported by in vitro experiments: *SMDT1* overexpression markedly suppressed cell migration and invasion. Although the precise mechanisms require further investigation, several biologically plausible explanations can be proposed. Mitochondrial calcium signaling is known to regulate epithelial–mesenchymal transition (EMT), cytoskeletal remodeling, and focal adhesion dynamics [27,28]. Reduced *SMDT1* expression may impair normal mitochondrial calcium uptake. Previous studies have shown that mitochondrial calcium signals can influence EMT by modulating calmodulin-dependent protein kinase (CaMK), Calcineurin/NFAT, PI3K–AKT, and reactive oxygen species (ROS)-related pathways, thereby promoting cancer cell migration and invasion [24,27,29]. Fernandez-Garcia et al. previously reported that MCU deficiency significantly alters cell cycle progression and metabolic state and affects tumorigenic capacity [11]. Integrating these findings with our results, we speculate that *SMDT1* loss may disrupt MCU complex stability, leading to impaired mitochondrial calcium uptake and subsequent induction of cytoskeletal reorganization and an invasive phenotype.

Our enrichment analyses further elucidated the molecular basis of *SMDT1*’s tumor-suppressive effects. Correlation analyses of co-expressed genes and proteins consistently demonstrated that *SMDT1*-associated genes are significantly enriched in pathways related to calcium signaling, oxidative phosphorylation, apoptosis, cellular senescence, and mitochondrial organization. Notably, oxidative phosphorylation has received increasing attention in thyroid cancer biology. The conventional view holds that thyroid cancers primarily rely on the Warburg effect for growth [30,31], but recent multi-omics studies indicate that PTC is not purely glycolysis-dependent and instead exhibits marked metabolic heterogeneity [32,33]. Related reviews have systematically summarized metabolic reprogramming features in THCA, noting that oxidative phosphorylation, fatty acid oxidation, and glutamine metabolism all contribute to tumor adaptation, and that the integrity of mitochondrial function is a key determinant of tumor metabolic plasticity [30,34,35]. Moreover, accumulating evidence suggests that under conditions of nutrient deprivation or therapeutic stress, thyroid cancer cells can shift toward oxidative phosphorylation-dependent metabolism to sustain survival and therapeutic resistance [34,36,37,38]. In this study, *SMDT1* was found to be significantly positively correlated with genes of the mitochondrial respiratory chain, and these genes are coordinately downregulated in THCA, suggesting that *SMDT1* may participate in the regulation of a thyroid cancer-specific mitochondrial metabolic network.

The PPI network analysis further reinforced the mechanistic relevance of *SMDT1* in maintaining mitochondrial biological homeostasis. *SMDT1* exhibited strong associations with canonical regulators of the mitochondrial calcium uptake (MICU) and mitochondrial calcium uptake regulator 1 (MCUR1), as well as with voltage-dependent anion channels (VDAC) and mitochondrial membrane transport proteins. These observations are consistent with prior studies in which *SMDT1* functions as a bridging component linking MCU to mitochondrial calcium uptake (MICU) regulatory subunits and thereby sustaining channel stability [16,18,39,40]. Disruption of this network could alter endoplasmic-reticulum-to-mitochondria calcium transfer, with downstream effects on mitochondrial membrane potential, ATP synthesis, ROS production, and apoptosis. Given the central roles of these processes in cancer progression, our findings indicate that *SMDT1* may represent a critical node connecting mitochondrial calcium signaling to tumor metabolic adaptation.

Another important finding is that *SMDT1* expression is significantly associated with immune cell infiltration levels in THCA. Tumors in the *SMDT1* high-expression group exhibited increased infiltration of CD8^+^ T cells, activated NK cells, memory B cells, monocytes, and eosinophils, while certain immune dysregulation-related subpopulations were relatively decreased. In recent years, immunometabolism has been recognized as a critical link between tumor cell metabolic states and the immune microenvironment. Previous investigators have proposed the concept of a tumor immune metabolic ecosystem, highlighting that mitochondrial metabolic reprogramming can modulate immune cell function via nutrient competition, metabolite release, and oxidative stress [41,42,43,44]. Concurrently, multiple studies have shown that mitochondrial dysfunction can lead to CD8^+^ T cell exhaustion, reduced NK cell cytotoxicity, and defects in antigen presentation, thereby facilitating immune evasion [45,46,47,48]. Notably, recent work further indicates that tumor cells can even impair T cell function through mitochondrial transfer, resulting in metabolic collapse of tumor-infiltrating lymphocytes and the establishment of an immunosuppressive state [49,50]. In light of our observation of increased infiltration of CD8^+^ T cells and activated NK cells, we hypothesize that *SMDT1* may indirectly promote antitumor immune responses by maintaining mitochondrial homeostasis and metabolic integrity. This finding suggests that *SMDT1* not only participates in intrinsic metabolic regulation of tumor cells but may also serve as an important molecular node linking metabolic reprogramming to immune microenvironment remodeling.

Compared with previous studies, this study indicates that *SMDT1* may have potential clinical utility as a biomarker and therapeutic target. ROC analysis demonstrated its diagnostic performance in THCA, while survival analysis indicated its ability to predict disease recurrence. As molecularly stratified management of thyroid cancer advances, incorporating mitochondrial functional markers such as *SMDT1* into predictive models may outperform models based solely on clinicopathological features and canonical driver mutations. Moreover, pharmacological modulation of mitochondrial calcium transport is being explored across multiple diseases, and targeting the MCU-*SMDT1* axis could represent a novel strategy to inhibit tumor progression or enhance antitumor immunity.

This study has several limitations. First, the clinical validation cohort is small and drawn from a single center, so the findings require confirmation in multi-center, large-sample cohorts. Second, only adult patients were included, leaving pediatric THCA unexamined. Since the molecular features and clinical presentations can vary with age, the role of SMDT1 in pediatric THCA remains to be determined. Third, although the GEO database was used for external validation, the transcriptome analyses rely mainly on retrospective public datasets such as TCGA, and prospective studies are needed for confirmation. Fourth, multivariate Cox regression indicated that SMDT1 is not an independent prognostic factor, possibly because its expression is partly influenced by clinical variables such as TNM stage; thus, its clinical utility should be reassessed in a larger cohort. Finally, the specific molecular mechanisms of SMDT1, particularly its involvement in mitochondrial calcium signaling and in modulating the immune microenvironment, remain to be elucidated through in vivo experiments and mechanistic studies.

## 5. Conclusions

In summary, this study systematically reveals for the first time a potential tumor-suppressive role of *SMDT1* in THCA. Its low expression is significantly associated with tumor progression, lymph node metastasis, and poor prognosis, whereas its overexpression markedly inhibits the malignant phenotype of PTC cells. Mechanistically, *SMDT1* may exert its tumor-suppressive effects by maintaining mitochondrial calcium homeostasis, regulating oxidative phosphorylation and apoptosis-related processes, and participating in the remodeling of the tumor immune microenvironment. Overall, our findings provide a solid theoretical basis for *SMDT1* as a molecular target for early diagnosis, prognostic assessment, and potential targeted intervention in thyroid cancer, thereby laying the groundwork for the development of future precision therapeutic strategies.

## Data Availability

The original contributions presented in this study are included in the article/Supplementary Materials. Further inquiries can be directed to the corresponding author.

## Supplementary Materials

The following supporting information can be downloaded at: https://www.mdpi.com/article/10.3390/diagnostics16142250/s1, Supplementary Figure S1: Correlation analysis of the top 10 co-expressed genes associated with *C22orf32* expression in THCA. Supplementary Figure S2: Expression levels of the top 10 genes co-expressed with *SMDT1* in THCA tissues and normal thyroid tissues. Non-Histone Protein 2-Like Protein 1 (*NHP2L1*) is also known as Small Nuclear Ribonucleoprotein 13 (*SNU13*). Supplementary Table S1: Clinical characteristics of the PTC patients. Supplementary Table S2: The primer sequences. Supplementary Table S3: The information of antibodies. Supplementary Table S4: The sequence information of the *SMDT1* overexpression plasmid. Supplementary Table S5: The pan-cancer expression analysis of *SMDT1*. Supplementary Table S6: Multivariate Cox analysis for predicting DFS of THCA. Supplementary Table S7: The top 50 genes positively and negatively correlated with *SMDT1* expression. Supplementary Original Images: Original images from the Western blotting, colony formation assay, wound healing assay and transwell assays.

## Abbreviations

The following abbreviations are used in this manuscript:

ATP	Adenosine Triphosphate
AUC	Area Under the Curve
BCA	Bicinchoninic Acid
BLCA	Bladder Carcinoma
BP	Biological Process
BRCA	Breast Carcinoma
CaMK	Calmodulin-dependent protein Kinase
*C22orf32*	Chromosome 22 Open Reading Frame 32
CC	Cellular Component
CCK-8	Cell Counting Kit-8
CESC	Cervical Squamous Cell Carcinoma and Endocervical Adenocarcinoma
CHOL	Cholangiocarcinoma
CI	Confidence Interval
COAD	Colon Adenocarcinoma
DFS	Disease-Free Survival
EMT	Epithelial–Mesenchymal Transition
EMRE	Essential MCU Regulator
FBS	Fetal Bovine Serum
GEPIA3	Gene Expression Profiling Interactive Analysis 3
GO	Gene Ontology
HNSC	Head and Neck Squamous Cell Carcinoma
HPA	Human Protein Atlas
HR	Hazard Ratio
IHC	Immunohistochemistry
KEGG	Kyoto Encyclopedia of Genes and Genomes
KICH	Kidney Chromophobe
KIRC	Kidney Renal Clear Cell Carcinoma
KIRP	Kidney Renal Papillary Cell Carcinoma
LIHC	Liver Hepatocellular Carcinoma
LUAD	Lung Adenocarcinoma
LUSC	Lung Squamous Cell Carcinoma
MCU	Mitochondrial Calcium Uniporter
MCUR1	Mitochondrial Calcium Uptake Regulator 1
MF	Molecular Function
MICU	Mitochondrial Calcium Uptake Protein
MN	Matched Normal Tissue
mRNA	Messenger Ribonucleic Acid
NFAT	Nuclear Factor of Activated T Cells
NHP2L1	Non-Histone Protein 2-Like Protein 1
NK	Natural Killer
PPI	Protein–Protein Interaction
PTC	Papillary Thyroid Carcinoma
PVDF	Polyvinylidene Fluoride
qRT-PCR	Quantitative Real-Time Polymerase Chain Reaction
RAS	Rat Sarcoma Viral Oncogene Homolog
RIPA	Radioimmunoprecipitation Assay
RNA	Ribonucleic Acid
ROC	Receiver Operating Characteristic
ROS	Reactive Oxygen Species
SD	Standard Deviation
SDS-PAGE	Sodium Dodecyl Sulfate–Polyacrylamide Gel Electrophoresis
SMDT1	Single-Pass Membrane Protein with Aspartate-Rich Tail 1
SNU13	Small Nuclear Ribonucleoprotein 13
STAD	Stomach Adenocarcinoma
STRING	Search Tool for the Retrieval of Interacting Genes
TCGA	The Cancer Genome Atlas
TERT	Telomerase Reverse Transcriptase
THCA	Thyroid Carcinoma
TNM	Tumor-Node-Metastasis
VDAC	Voltage-Dependent Anion Channel
WB	Western Blotting
