# Supplementary material for: Integrated Bioinformatics and Experimental Validation Reveal the Diagnostic and Prognostic Value of SMDT1 in Thyroid Carcinoma
Source: Diagnostics (Basel). 2026 Jul 18;16(14):2250. doi: 10.3390/diagnostics16142250 (PMC13409411; doi:10.3390/diagnostics16142250)
Supplement: Supplementary file 1 [file diagnostics-16-02250-s001.zip › Supplementary original images/Western blotting original images/WB original images-SMDT1.pdf]

1 Figure 7C:

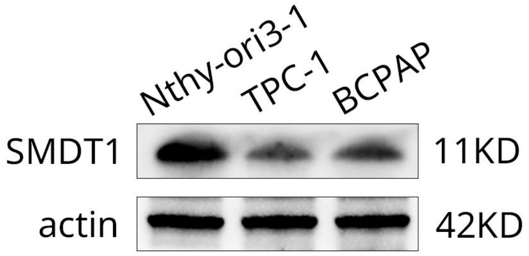

1.1 SMDT1 (11KD):

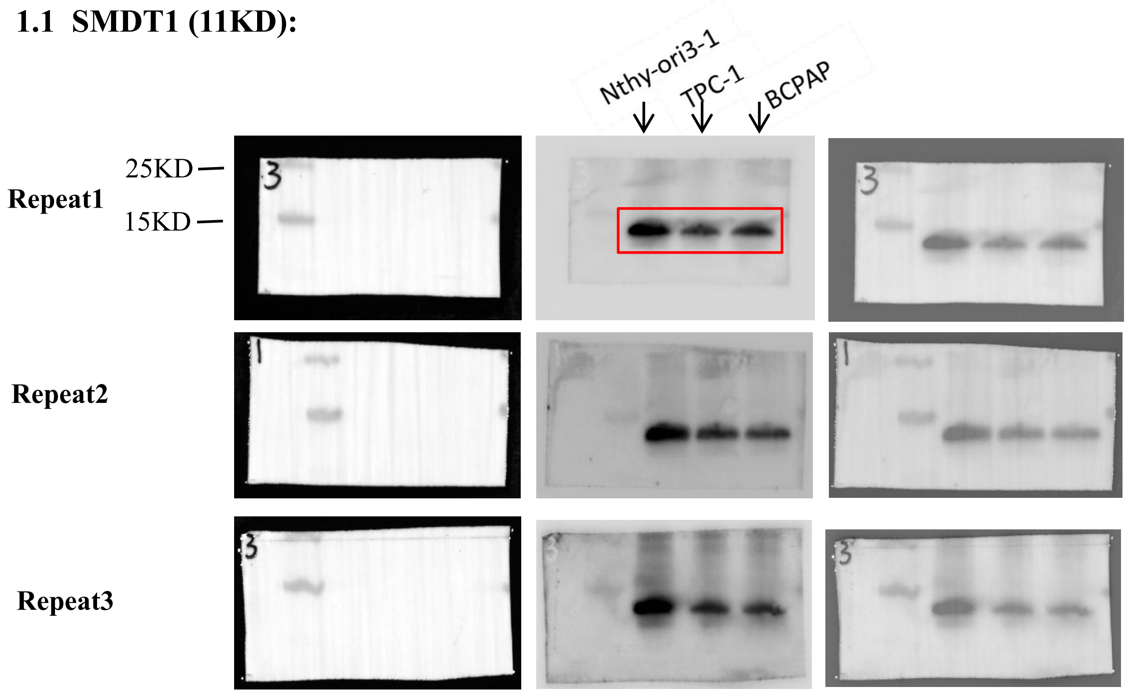

SMDT1 Marker **SMDT1 11KD**

1.2 actin (42KD):

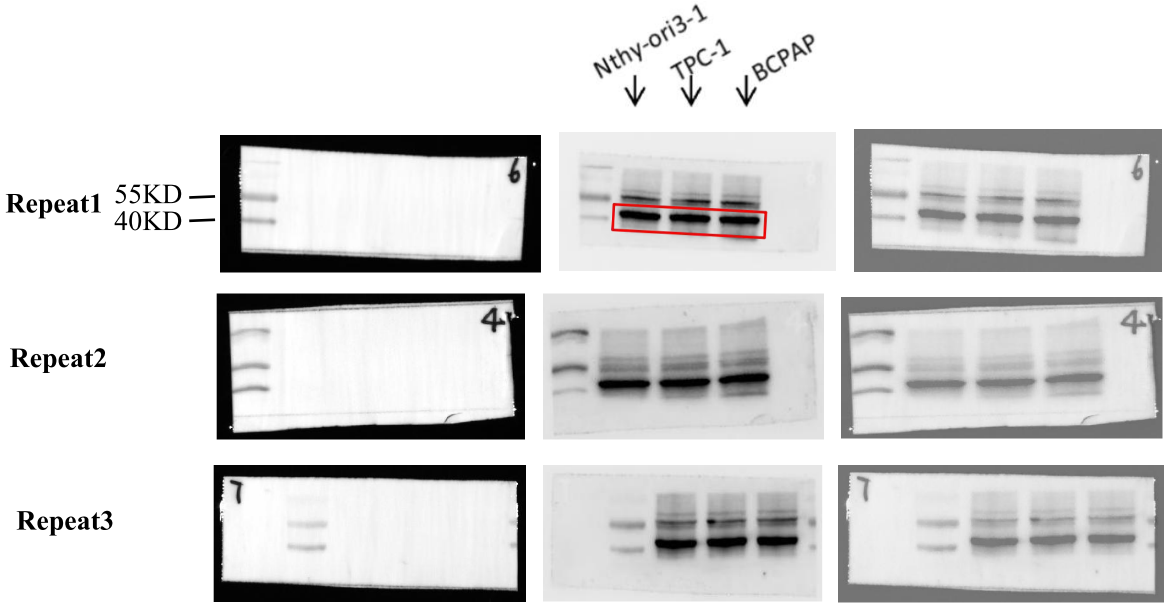

actin Marker **actin 42KD**

2 Figure 7F:

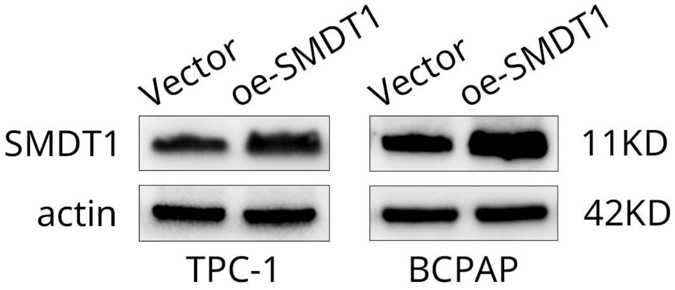

2.1 TPC-1:

SMDT1 (11KD):

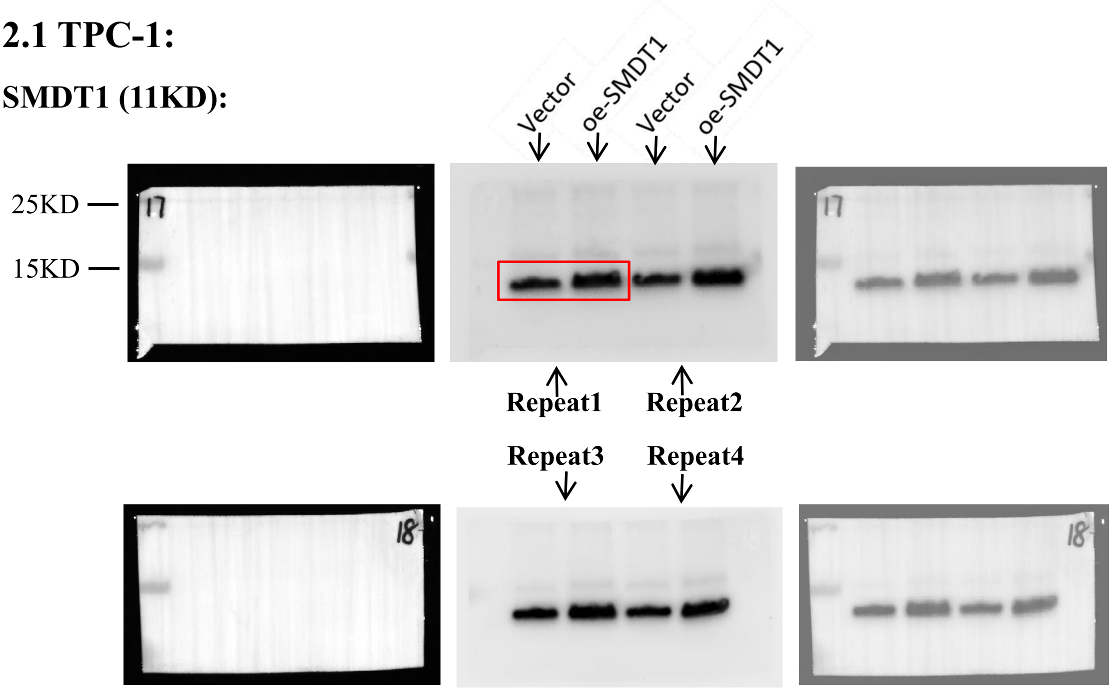

TPC-1 SMDT1 Marker **TPC-1 SMDT1 11KD**

actin (42KD):

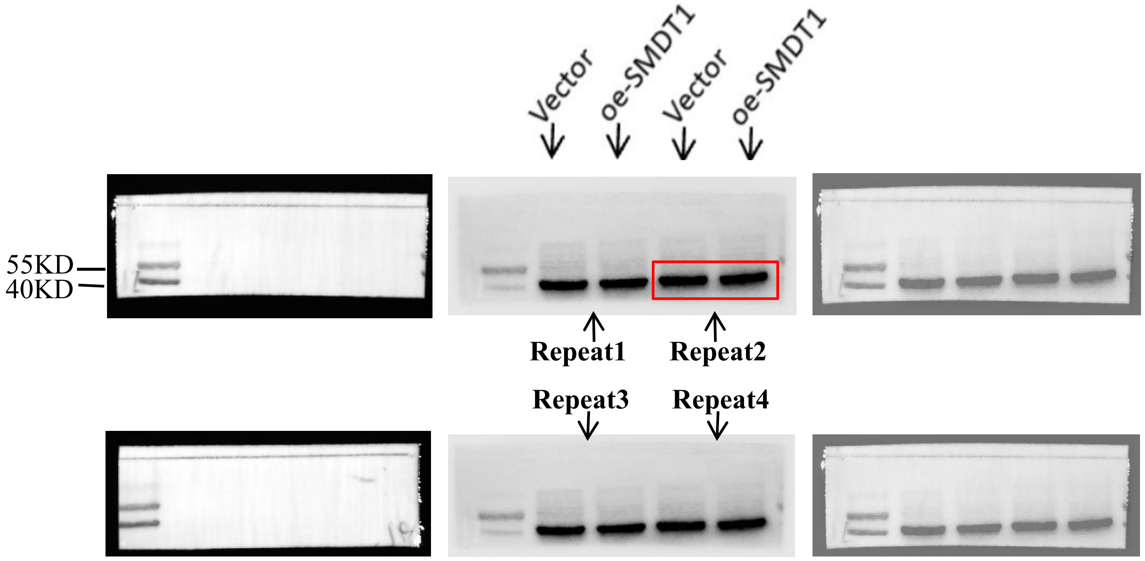

TPC-1 actin Marker **TPC-1 actin 42KD**

2.2 BCPAP:

SMDT1 (11KD):

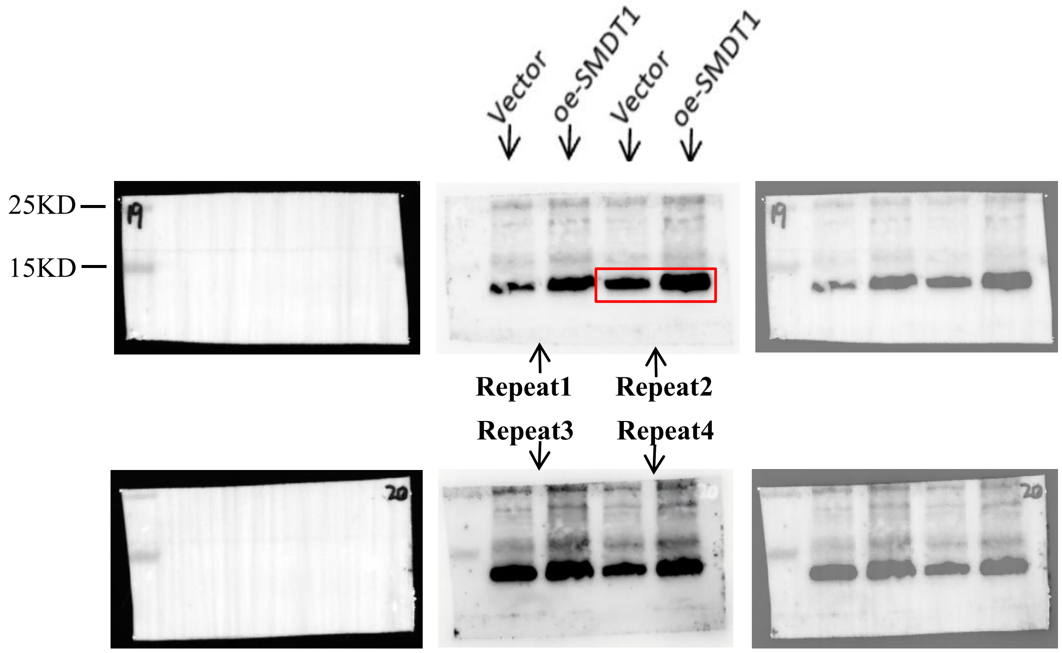

BCPAP SMDT1 Marker BCPAP SMDT1 11KD

actin (42KD):

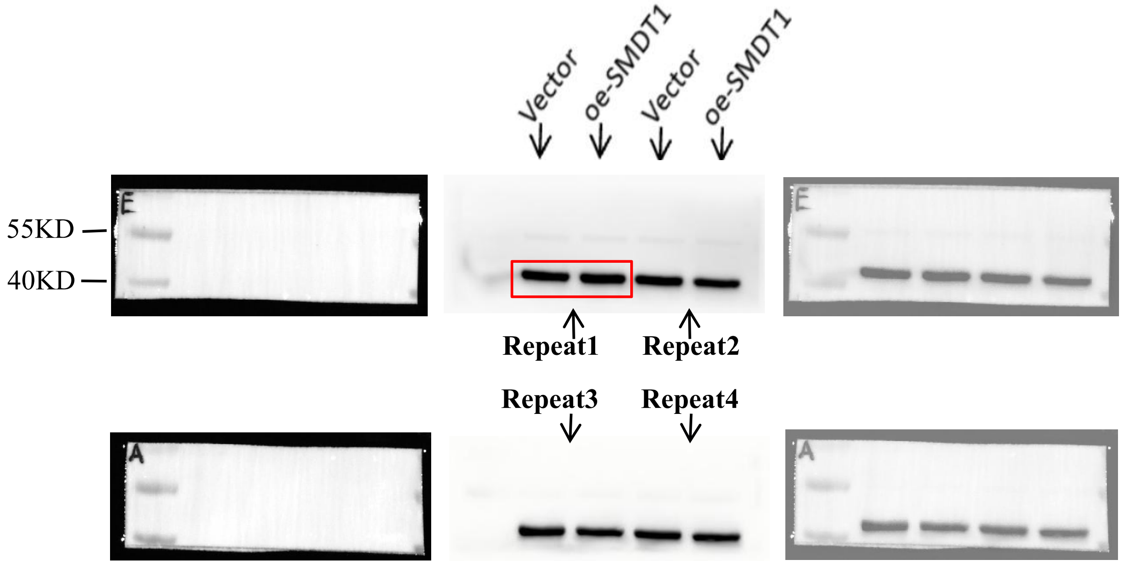

BCPAP actin Marker BCPAP actin 42KD
