# Supplementary figures and images for: Integrated Bioinformatics and Experimental Validation Reveal the Diagnostic and Prognostic Value of SMDT1 in Thyroid Carcinoma
Source: Diagnostics (Basel). 2026 Jul 18;16(14):2250. doi: 10.3390/diagnostics16142250 (PMC13409411; doi:10.3390/diagnostics16142250)

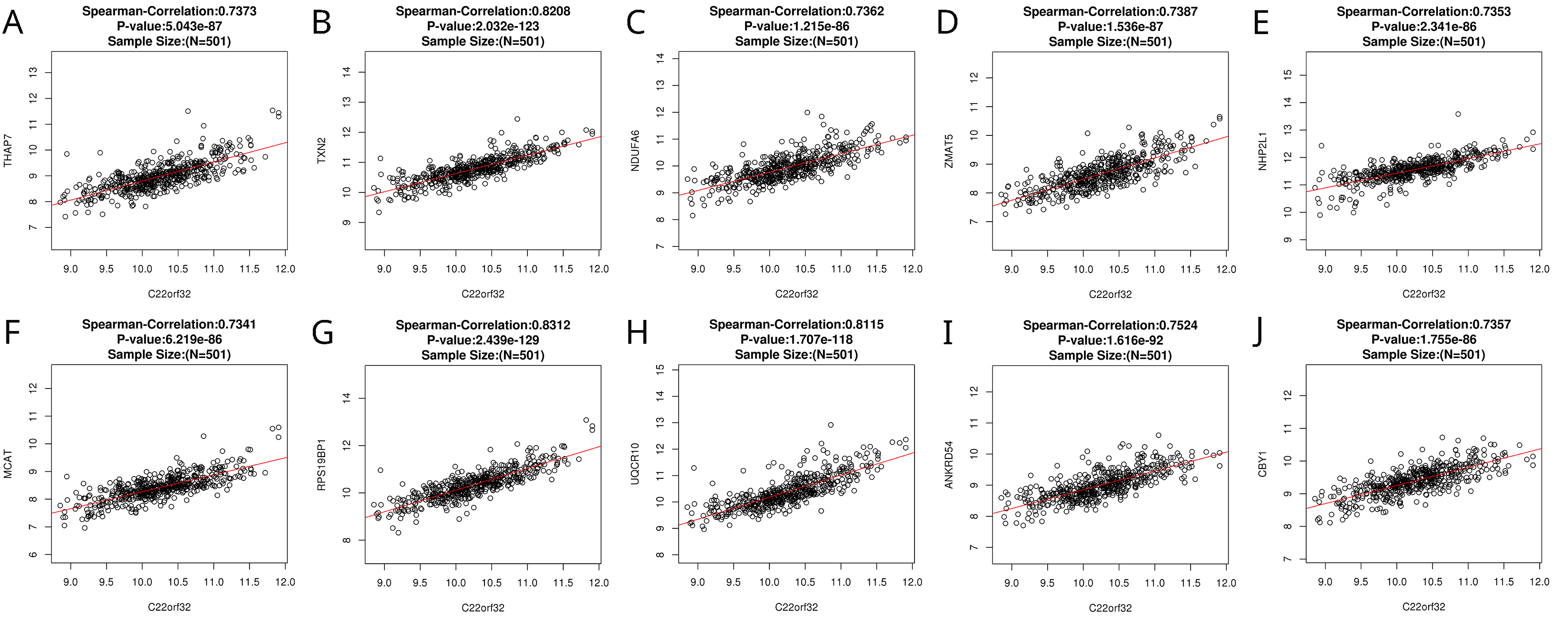

Supplement: Supplementary file 1 [file diagnostics-16-02250-s001.zip › Supplementary Figure S1.tif]

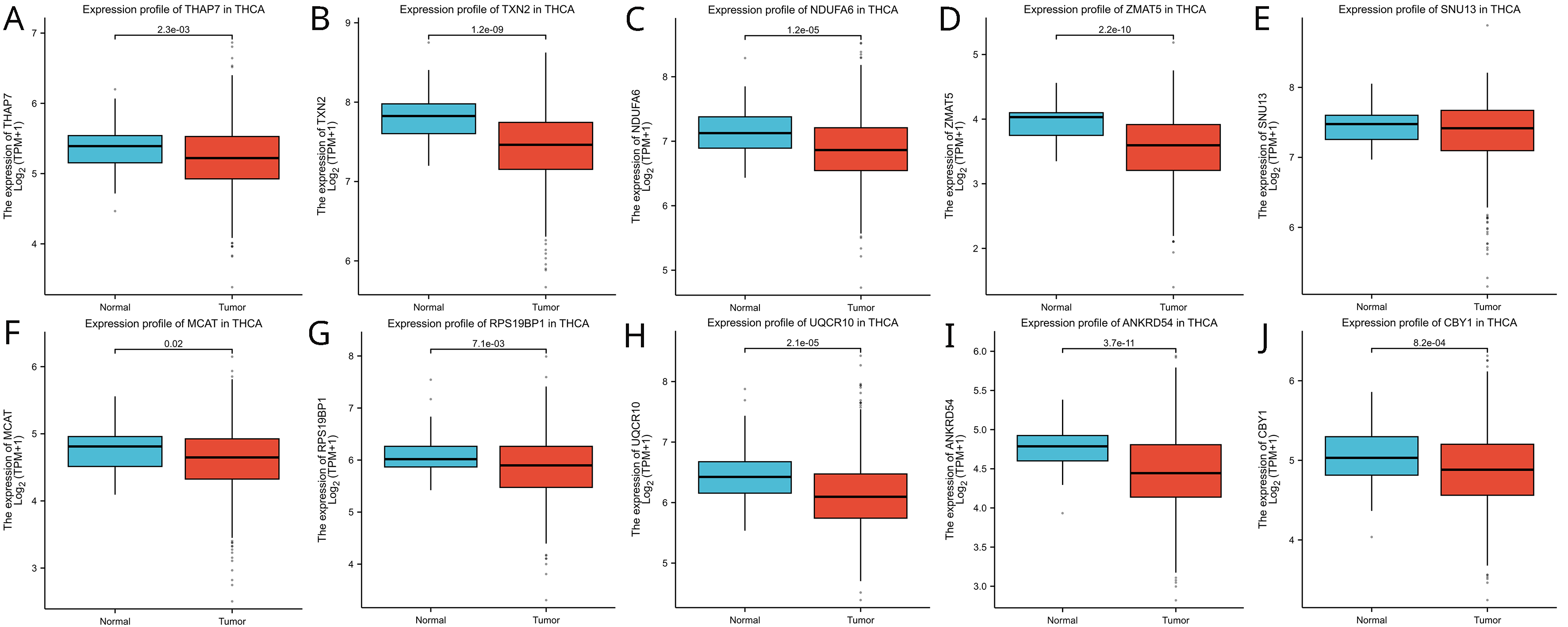

Supplement: Supplementary file 1 [file diagnostics-16-02250-s001.zip › Supplementary Figure S2.tif]

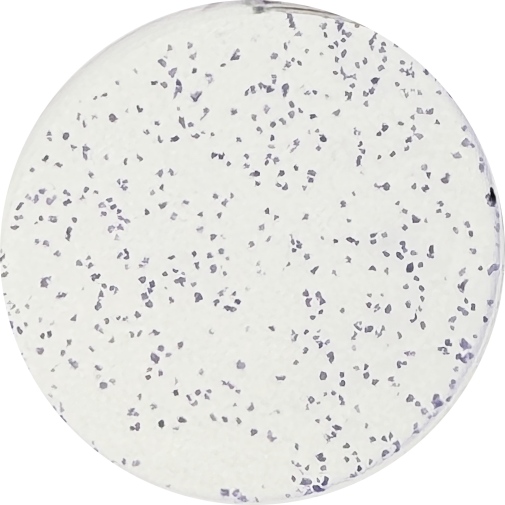

Supplement: Supplementary file 1 [file diagnostics-16-02250-s001.zip › Supplementary original images/Colony formation assay original images/BCPAP oe-SMDT1 (Repeat1).jpg]

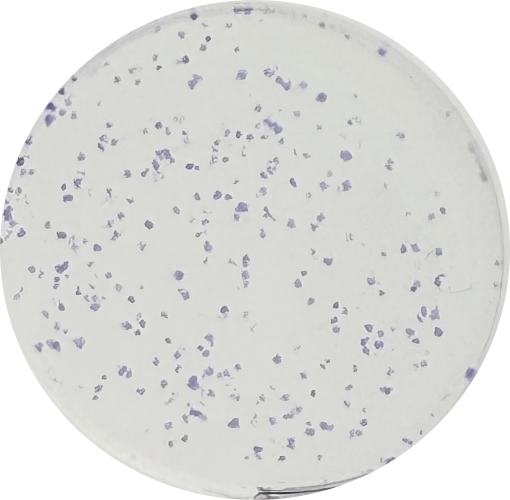

Supplement: Supplementary file 1 [file diagnostics-16-02250-s001.zip › Supplementary original images/Colony formation assay original images/BCPAP oe-SMDT1 (Repeat2).jpg]

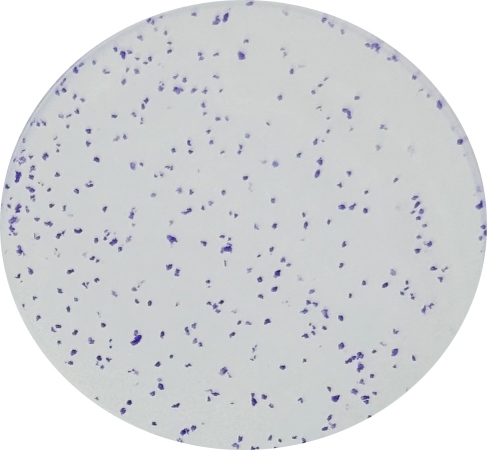

Supplement: Supplementary file 1 [file diagnostics-16-02250-s001.zip › Supplementary original images/Colony formation assay original images/BCPAP oe-SMDT1 (Repeat3).jpg]

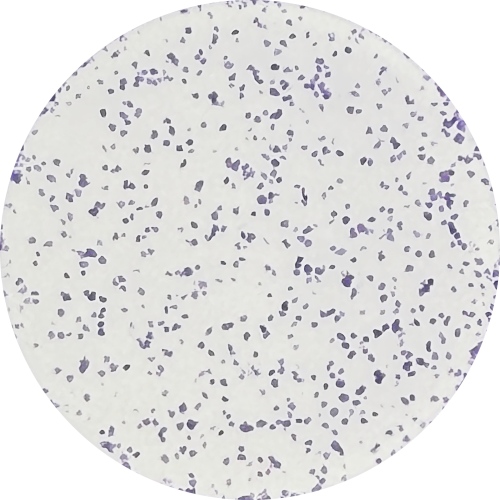

Supplement: Supplementary file 1 [file diagnostics-16-02250-s001.zip › Supplementary original images/Colony formation assay original images/BCPAP Vector (Repeat1).jpg]

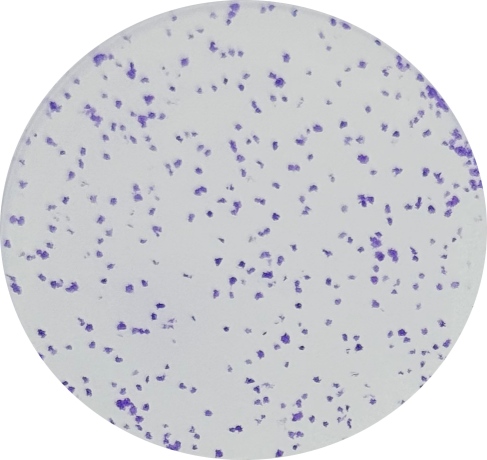

Supplement: Supplementary file 1 [file diagnostics-16-02250-s001.zip › Supplementary original images/Colony formation assay original images/BCPAP Vector (Repeat2).jpg]

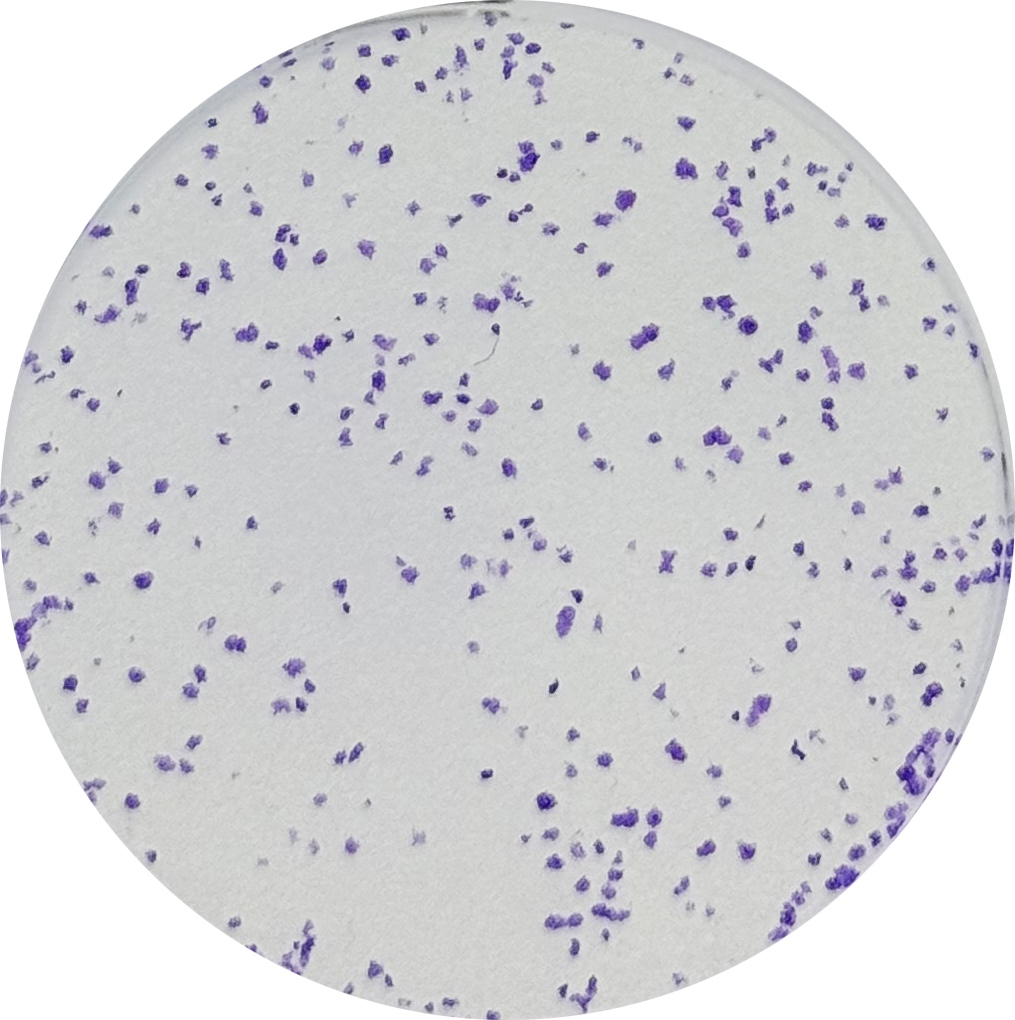

Supplement: Supplementary file 1 [file diagnostics-16-02250-s001.zip › Supplementary original images/Colony formation assay original images/BCPAP Vector (Repeat3).jpg]

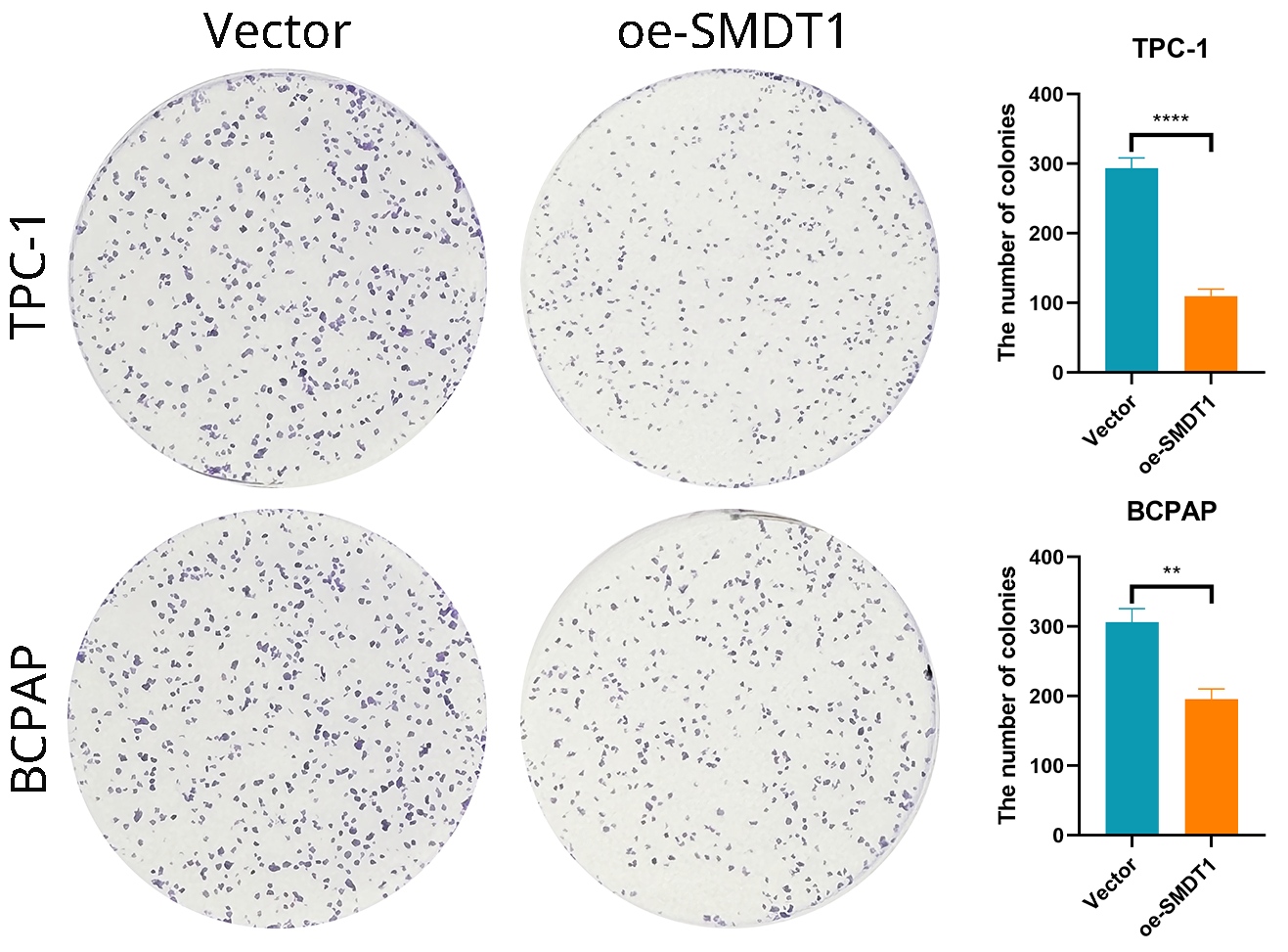

Supplement: Supplementary file 1 [file diagnostics-16-02250-s001.zip › Supplementary original images/Colony formation assay original images/Figure 8B.jpg]

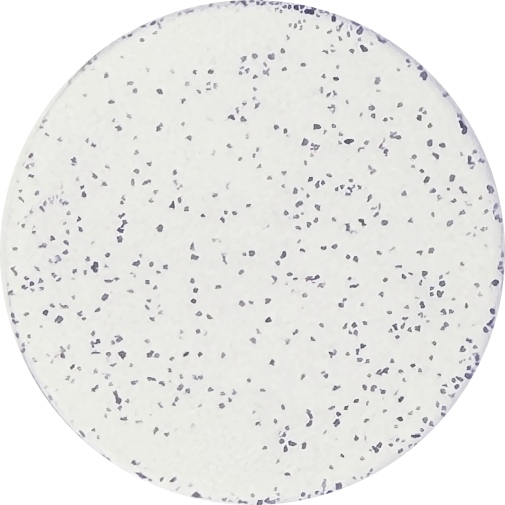

Supplement: Supplementary file 1 [file diagnostics-16-02250-s001.zip › Supplementary original images/Colony formation assay original images/TPC-1 oe-SMDT1 (Repeat1).jpg]

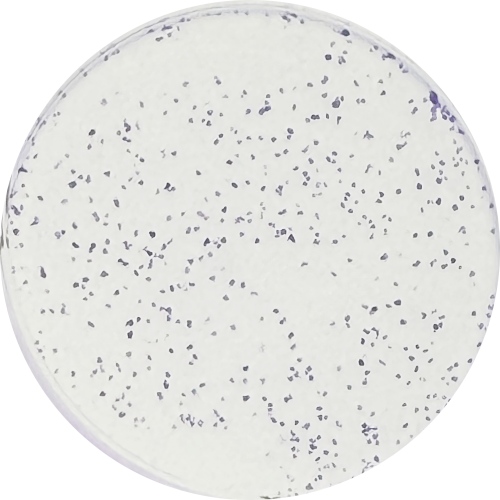

Supplement: Supplementary file 1 [file diagnostics-16-02250-s001.zip › Supplementary original images/Colony formation assay original images/TPC-1 oe-SMDT1 (Repeat2).jpg]

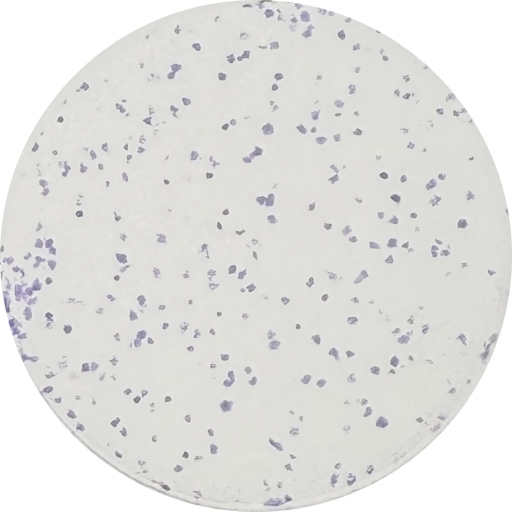

Supplement: Supplementary file 1 [file diagnostics-16-02250-s001.zip › Supplementary original images/Colony formation assay original images/TPC-1 oe-SMDT1 (Repeat3).jpg]

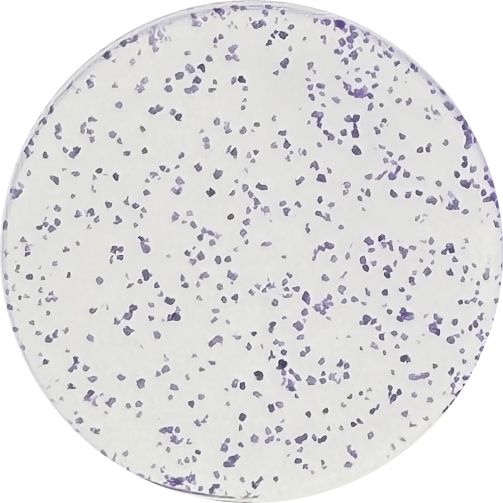

Supplement: Supplementary file 1 [file diagnostics-16-02250-s001.zip › Supplementary original images/Colony formation assay original images/TPC-1 Vector (Repeat1).jpg]

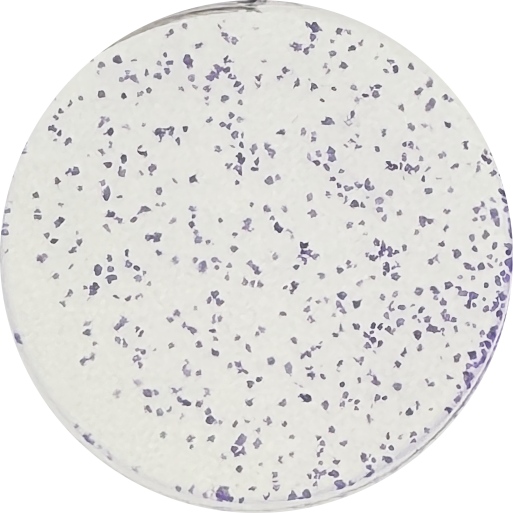

Supplement: Supplementary file 1 [file diagnostics-16-02250-s001.zip › Supplementary original images/Colony formation assay original images/TPC-1 Vector (Repeat2).jpg]

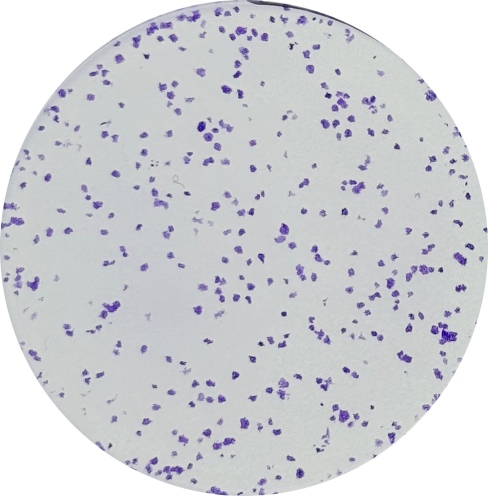

Supplement: Supplementary file 1 [file diagnostics-16-02250-s001.zip › Supplementary original images/Colony formation assay original images/TPC-1 Vector (Repeat3).jpg]

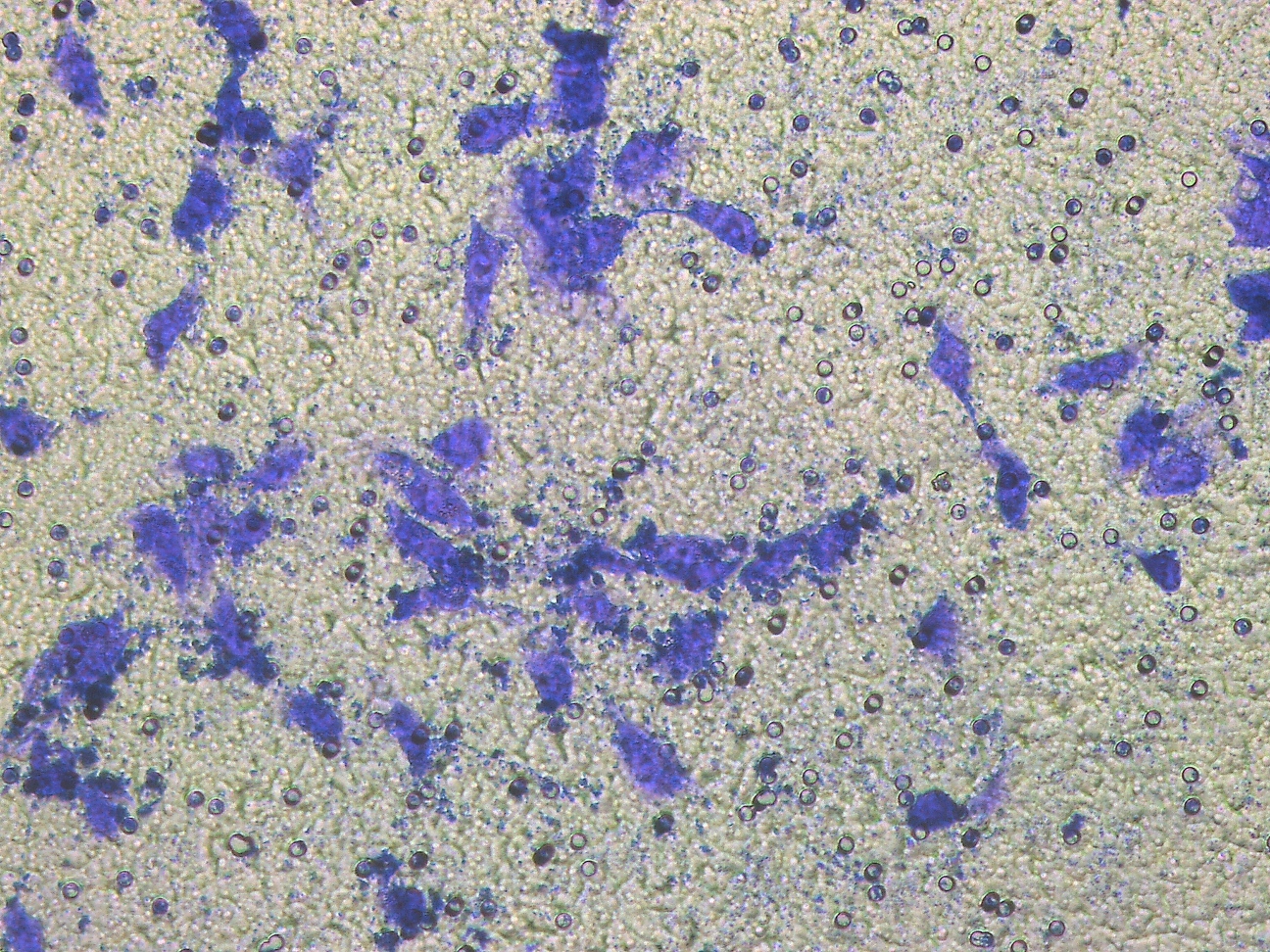

Supplement: Supplementary file 1 [file diagnostics-16-02250-s001.zip › Supplementary original images/Transwell assay original images/BCPAP oe-SMDT1 (Repeat1).jpg]

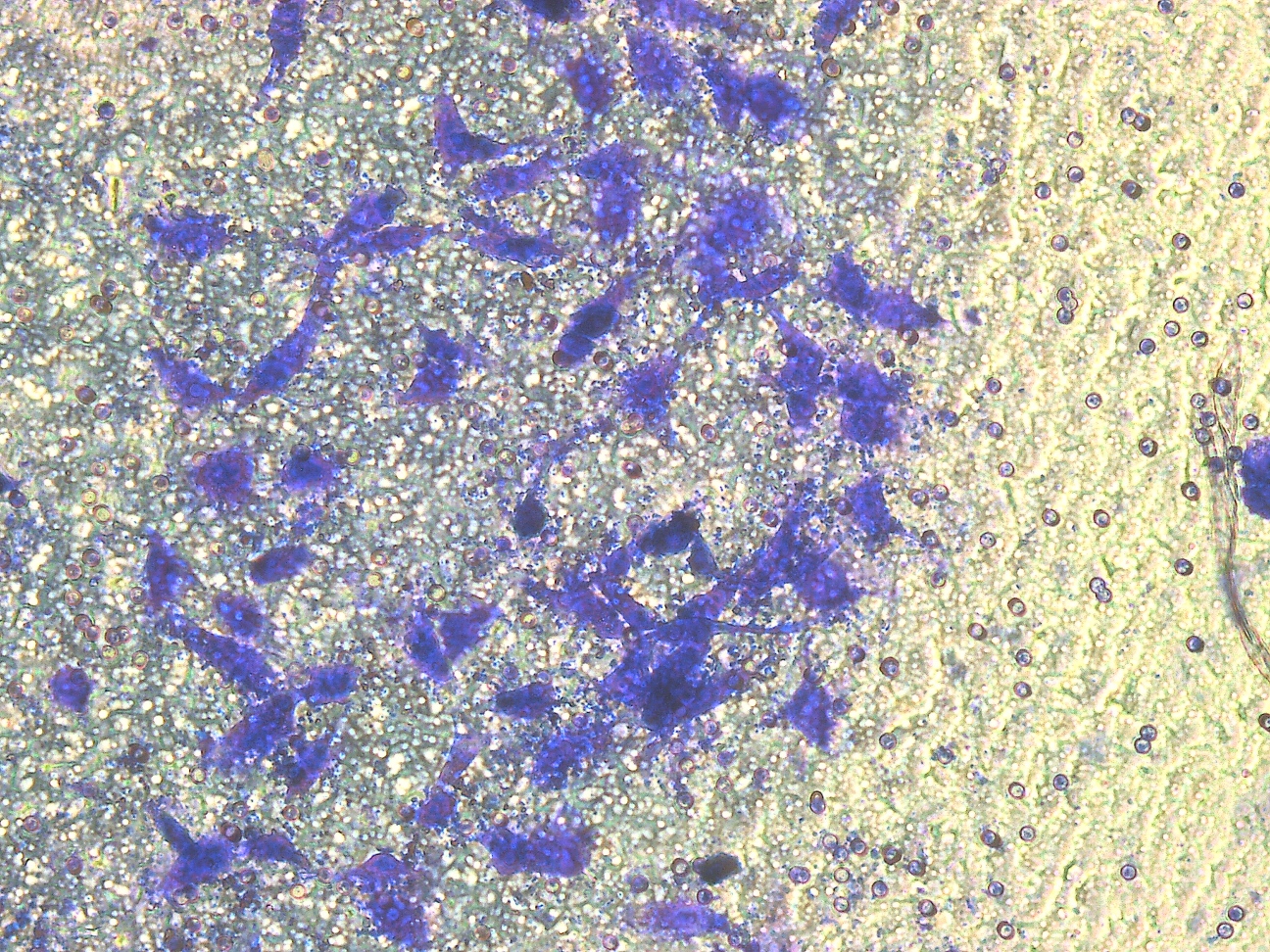

Supplement: Supplementary file 1 [file diagnostics-16-02250-s001.zip › Supplementary original images/Transwell assay original images/BCPAP oe-SMDT1 (Repeat2).jpg]

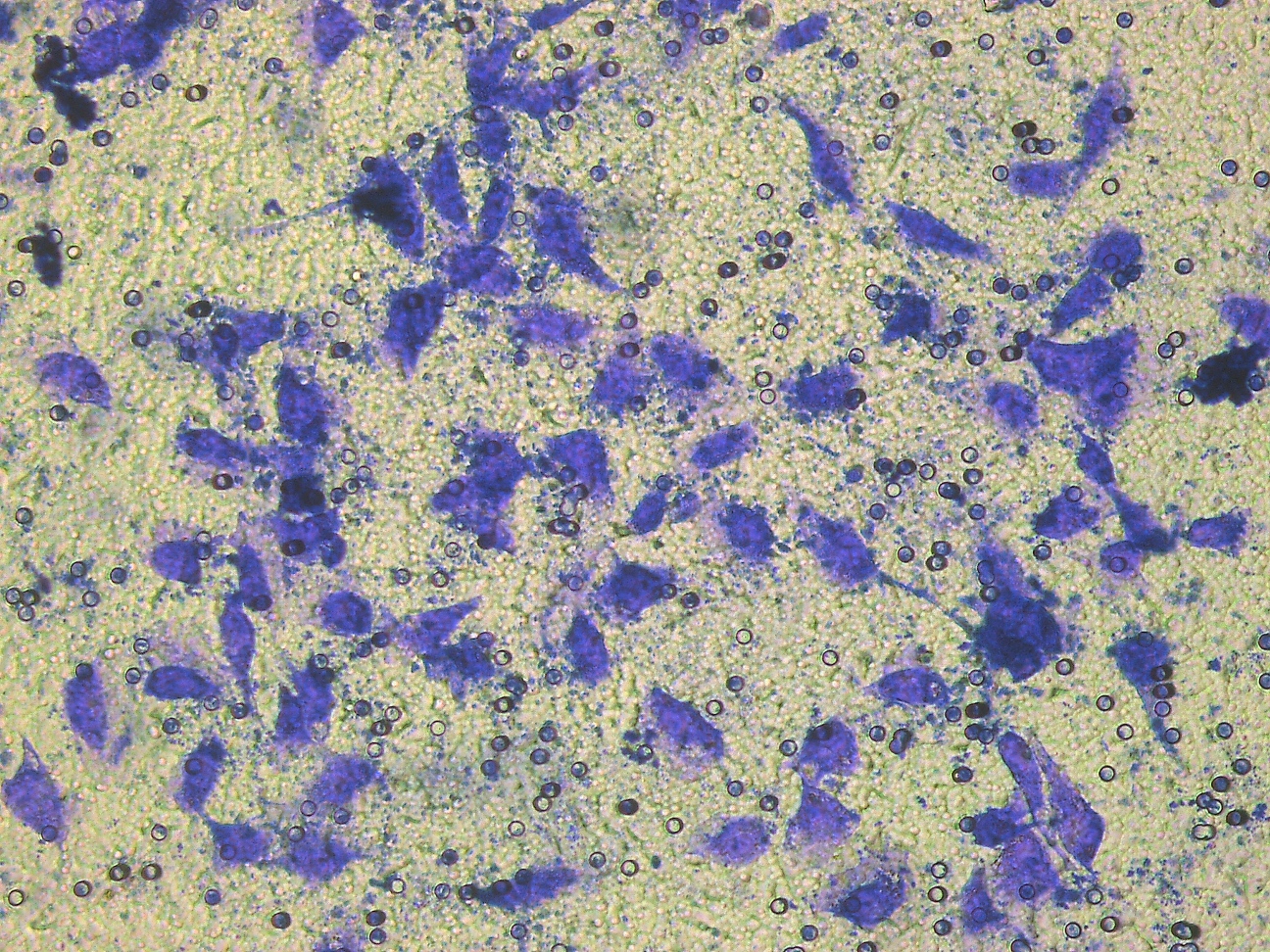

Supplement: Supplementary file 1 [file diagnostics-16-02250-s001.zip › Supplementary original images/Transwell assay original images/BCPAP oe-SMDT1 (Repeat3).jpg]

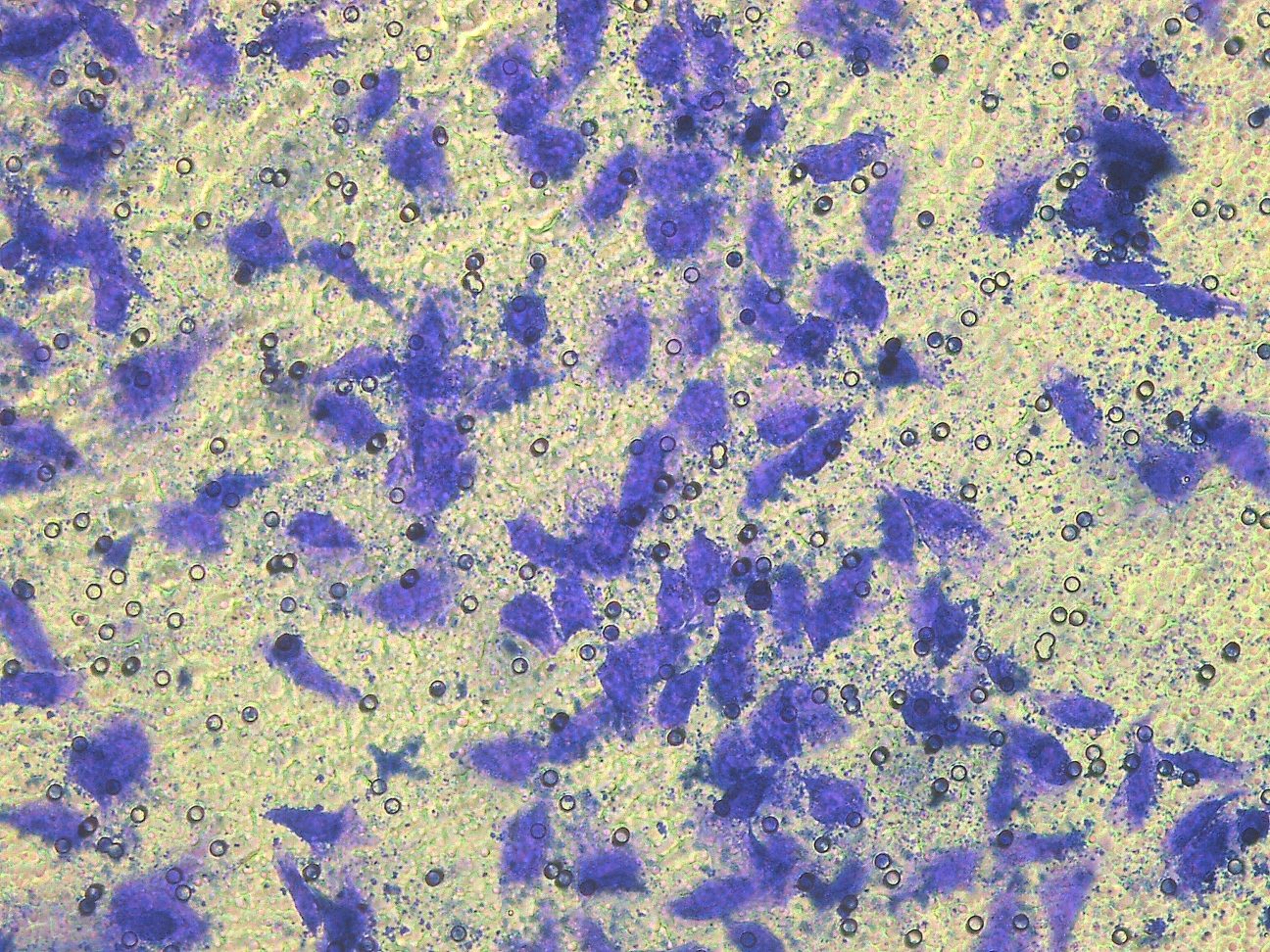

Supplement: Supplementary file 1 [file diagnostics-16-02250-s001.zip › Supplementary original images/Transwell assay original images/BCPAP Vector (Repeat1).jpg]

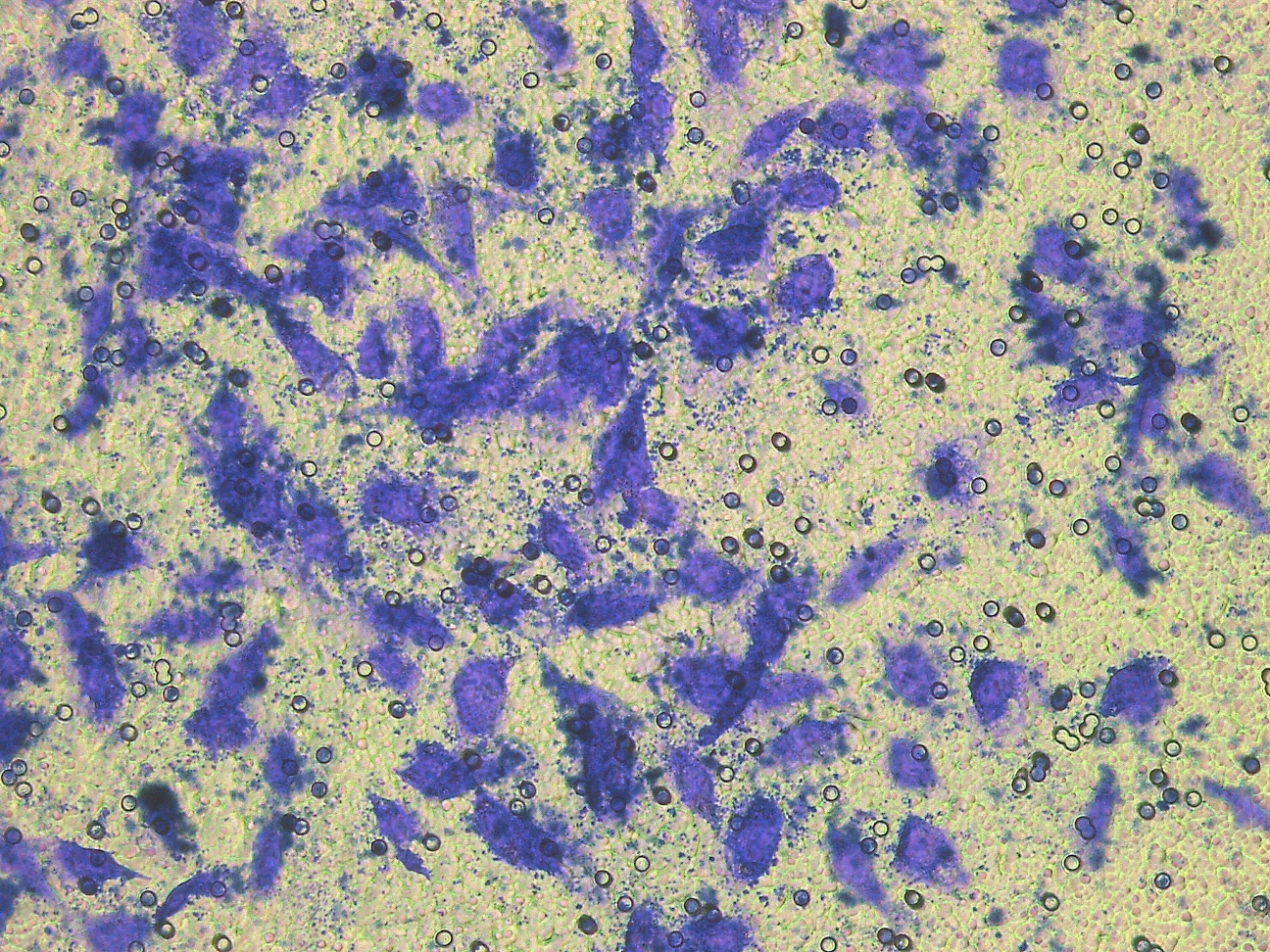

Supplement: Supplementary file 1 [file diagnostics-16-02250-s001.zip › Supplementary original images/Transwell assay original images/BCPAP Vector (Repeat2).jpg]

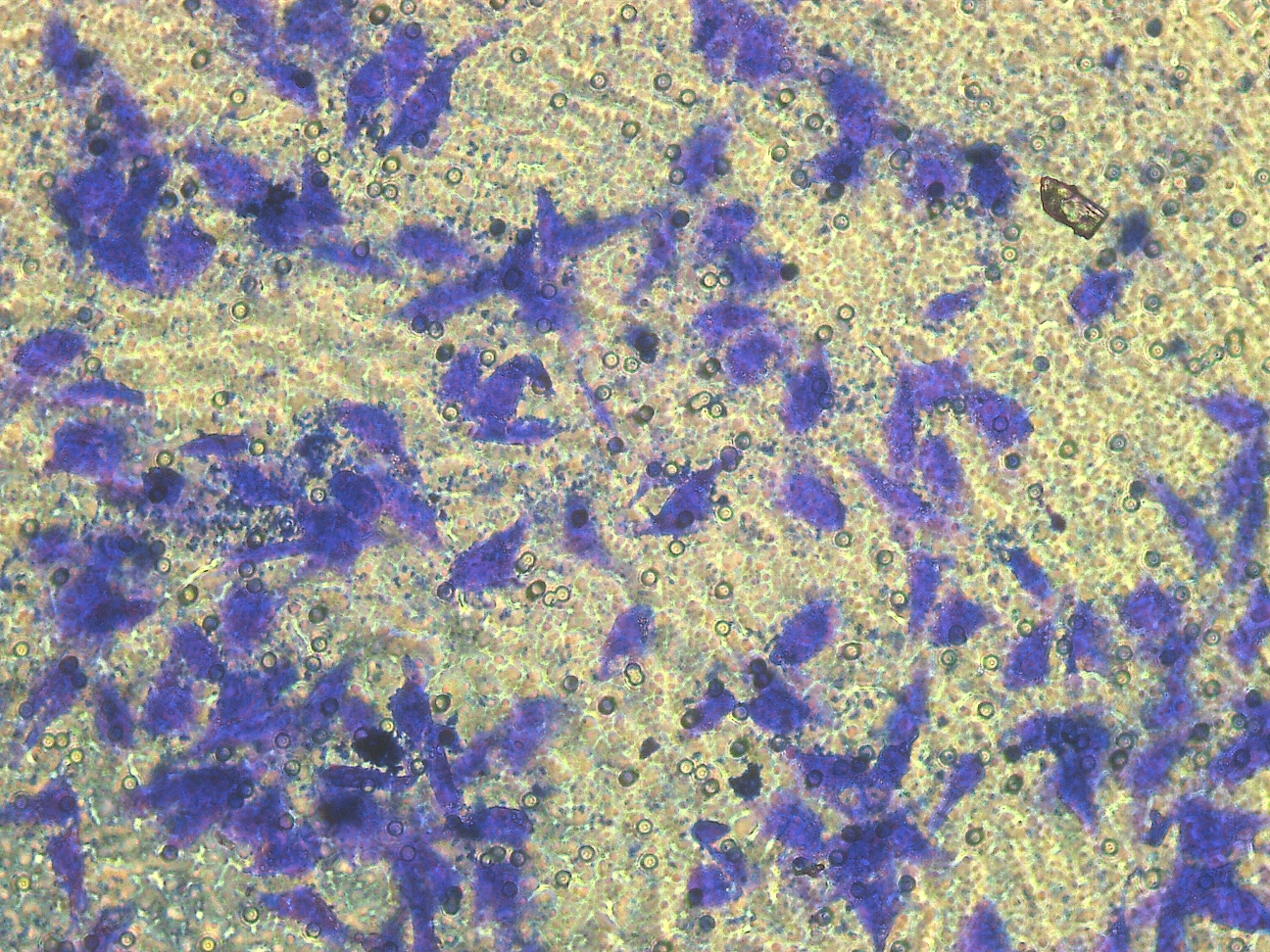

Supplement: Supplementary file 1 [file diagnostics-16-02250-s001.zip › Supplementary original images/Transwell assay original images/BCPAP Vector (Repeat3).jpg]

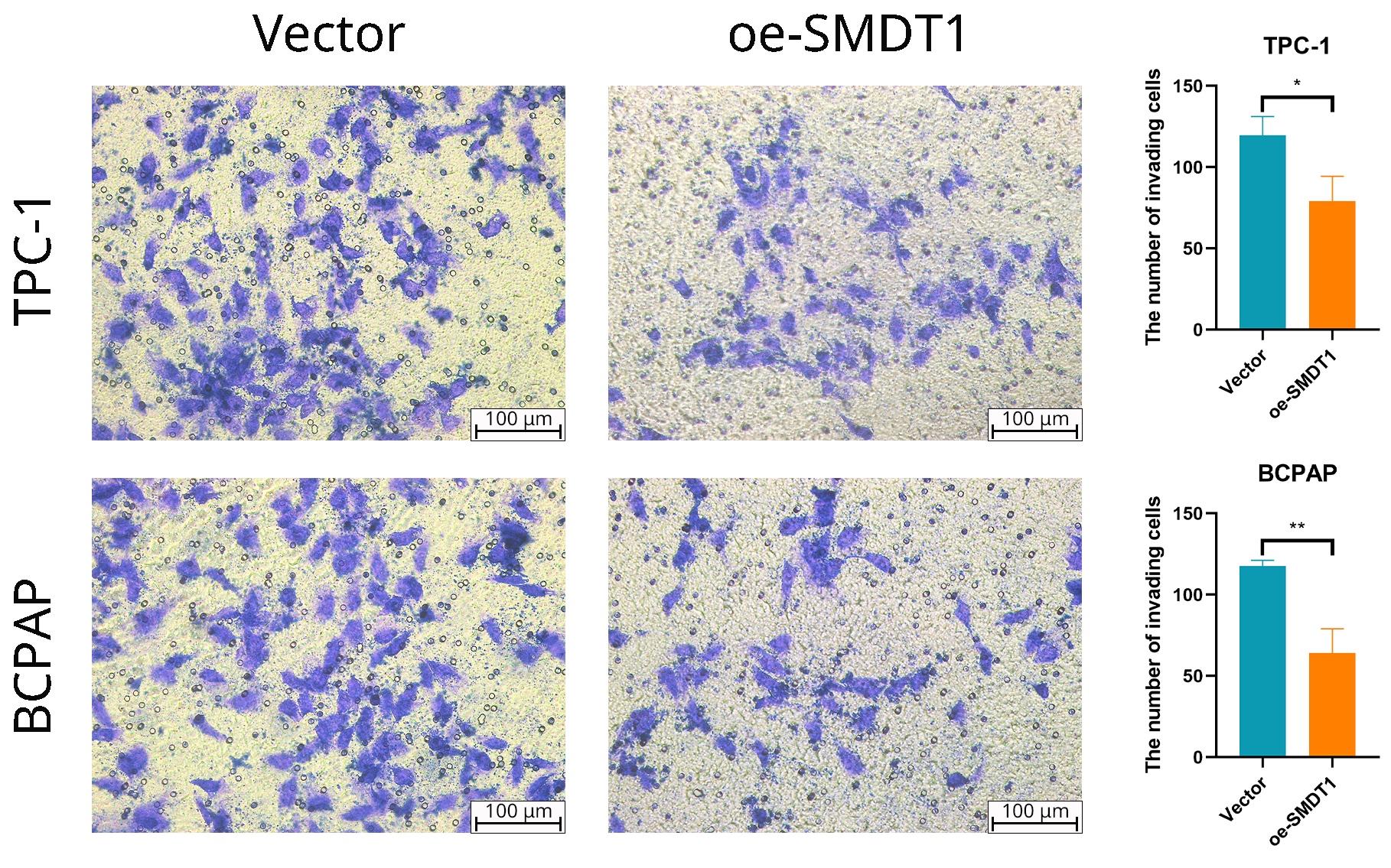

Supplement: Supplementary file 1 [file diagnostics-16-02250-s001.zip › Supplementary original images/Transwell assay original images/Figure 8D.jpg]

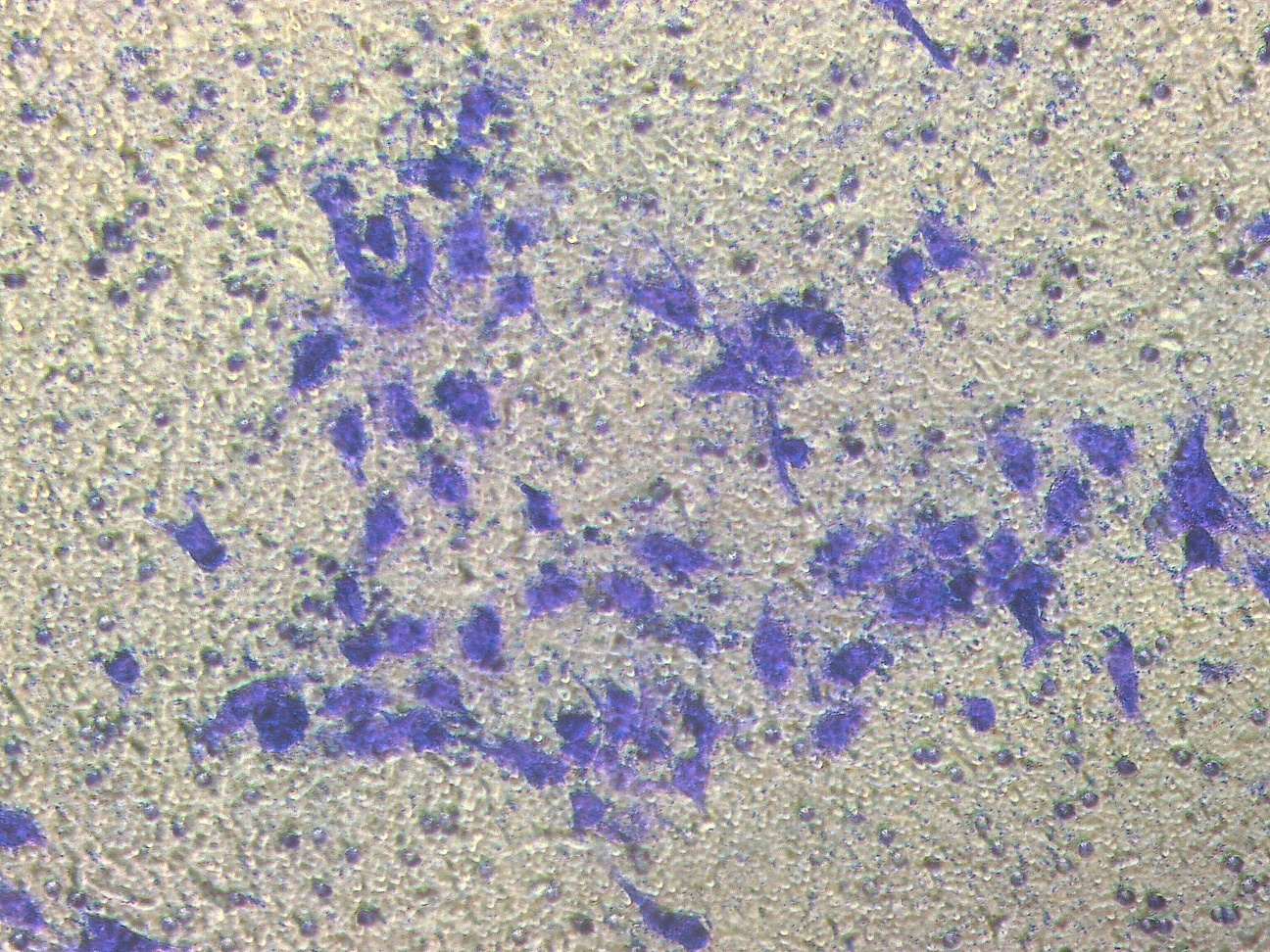

Supplement: Supplementary file 1 [file diagnostics-16-02250-s001.zip › Supplementary original images/Transwell assay original images/TPC-1 oe-SMDT1 (Repeat1).jpg]

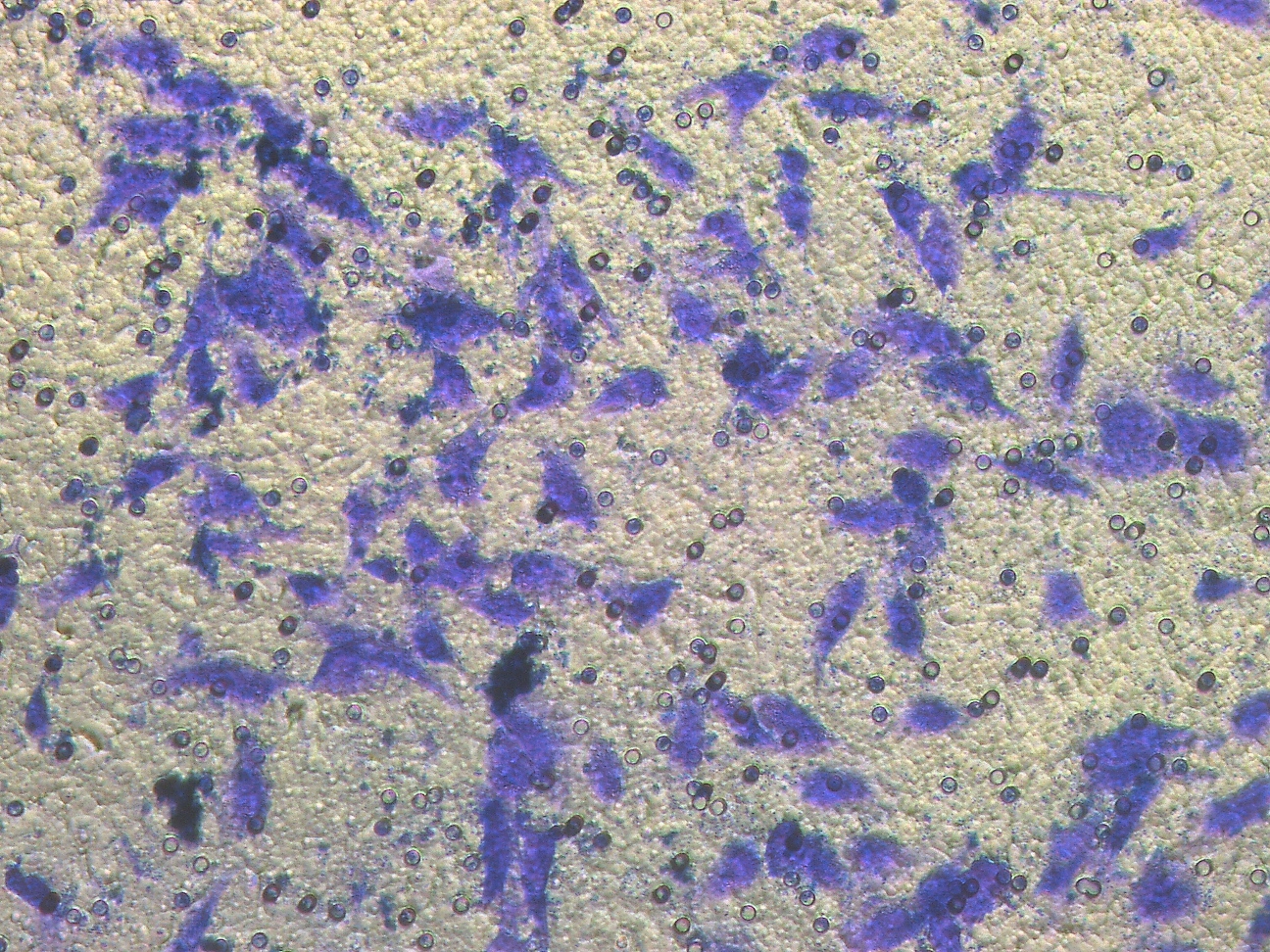

Supplement: Supplementary file 1 [file diagnostics-16-02250-s001.zip › Supplementary original images/Transwell assay original images/TPC-1 oe-SMDT1 (Repeat2).jpg]

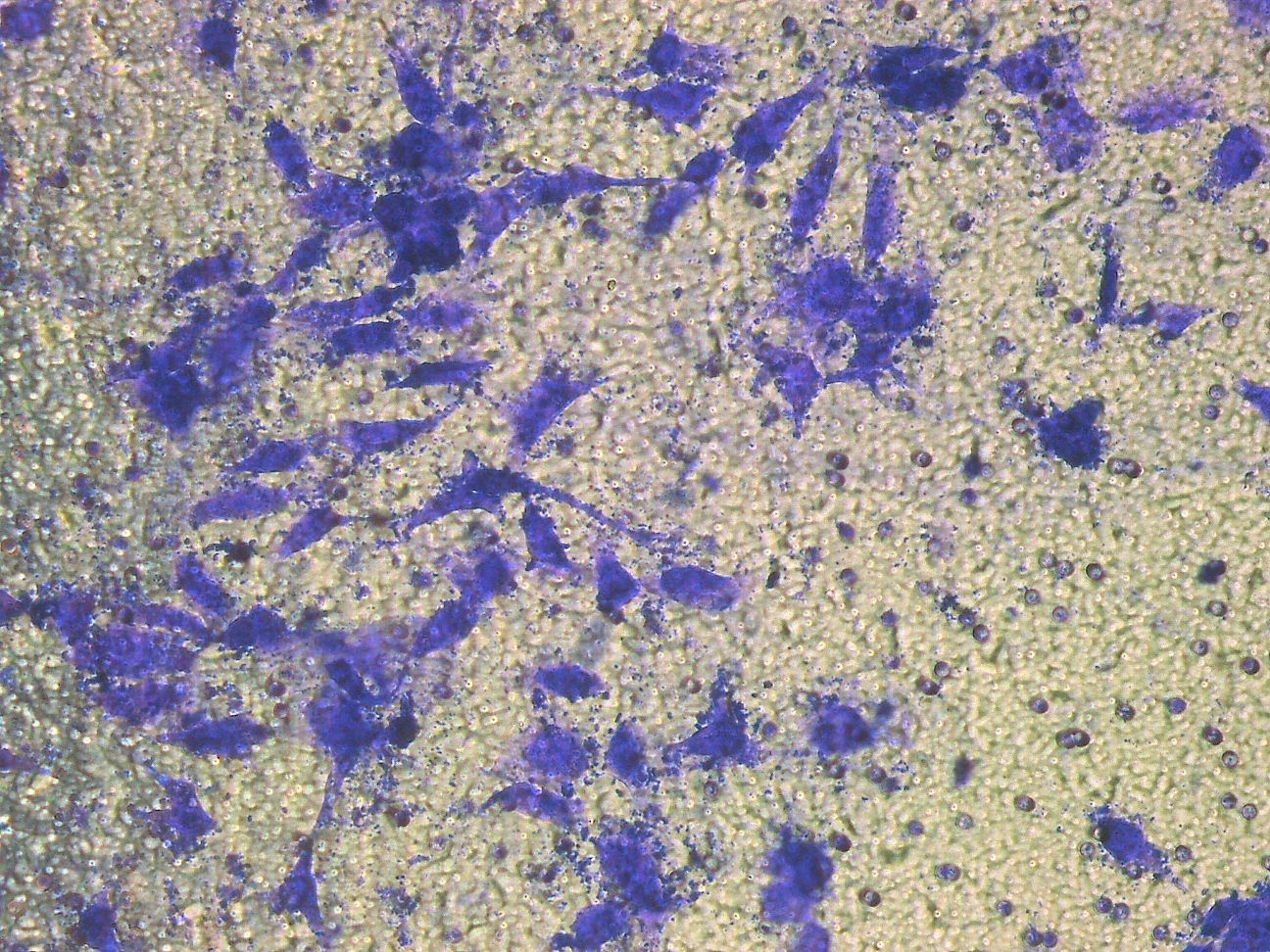

Supplement: Supplementary file 1 [file diagnostics-16-02250-s001.zip › Supplementary original images/Transwell assay original images/TPC-1 oe-SMDT1 (Repeat3).jpg]

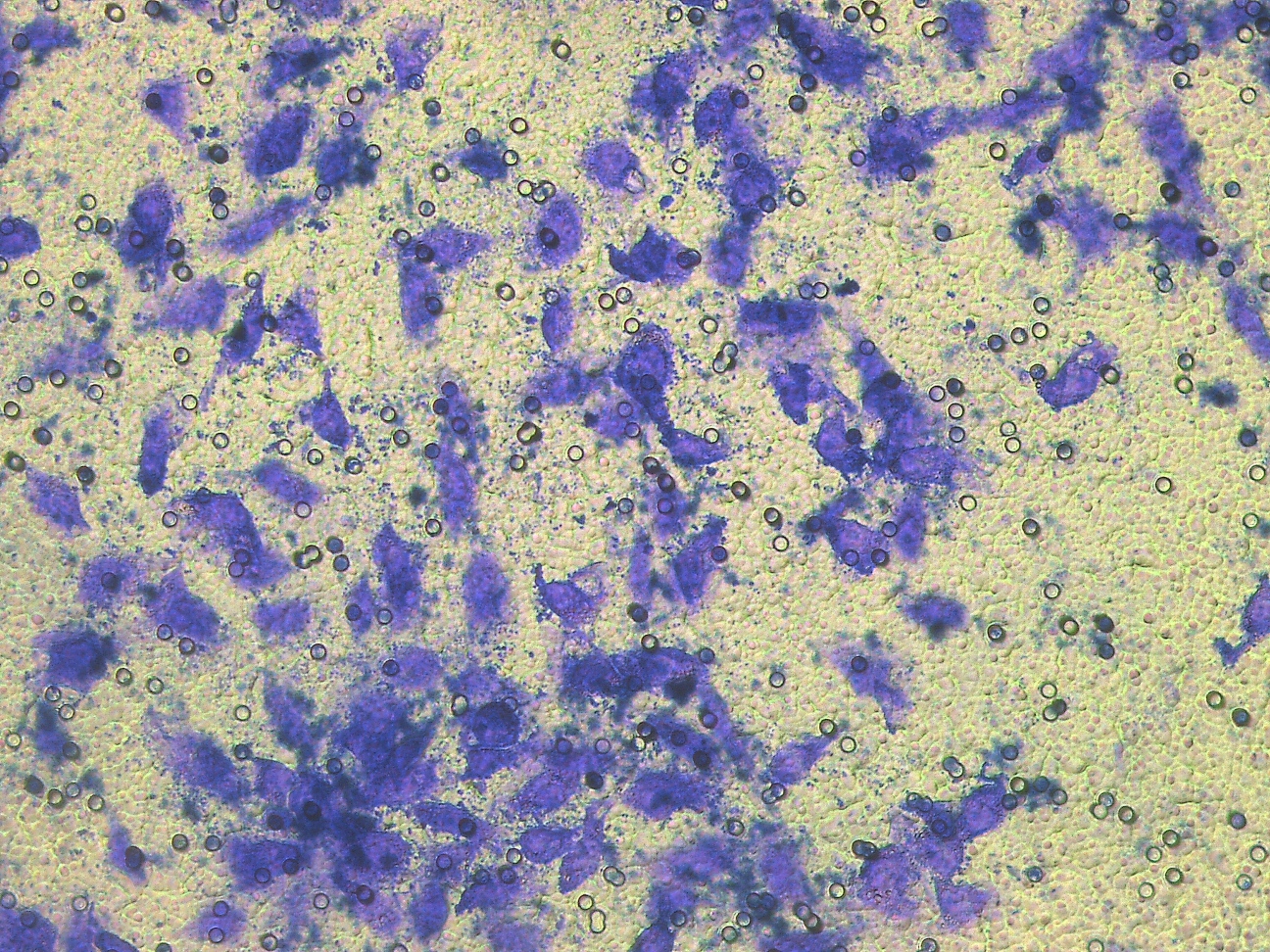

Supplement: Supplementary file 1 [file diagnostics-16-02250-s001.zip › Supplementary original images/Transwell assay original images/TPC-1 Vector (Repeat1).jpg]

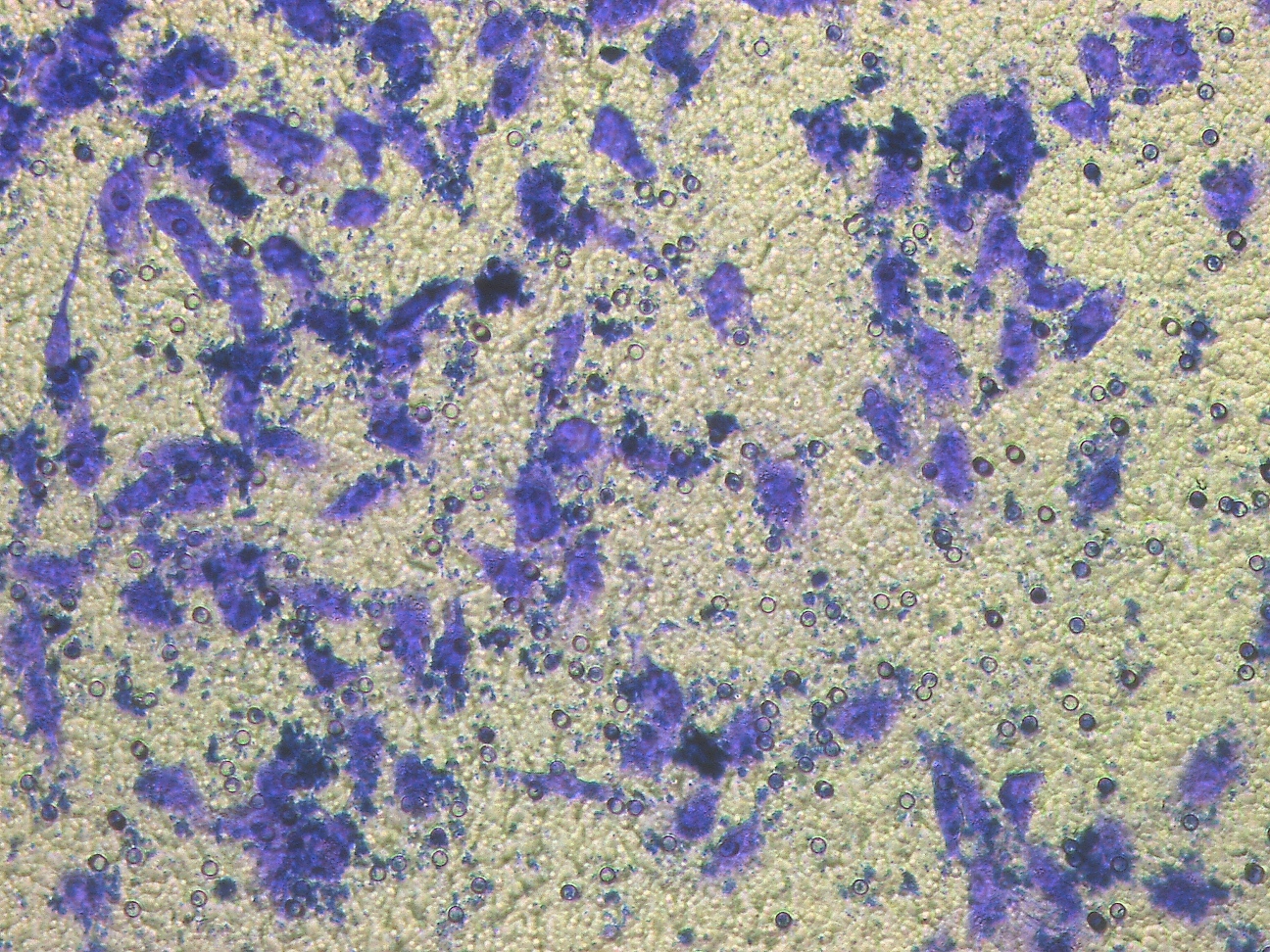

Supplement: Supplementary file 1 [file diagnostics-16-02250-s001.zip › Supplementary original images/Transwell assay original images/TPC-1 Vector (Repeat2).jpg]

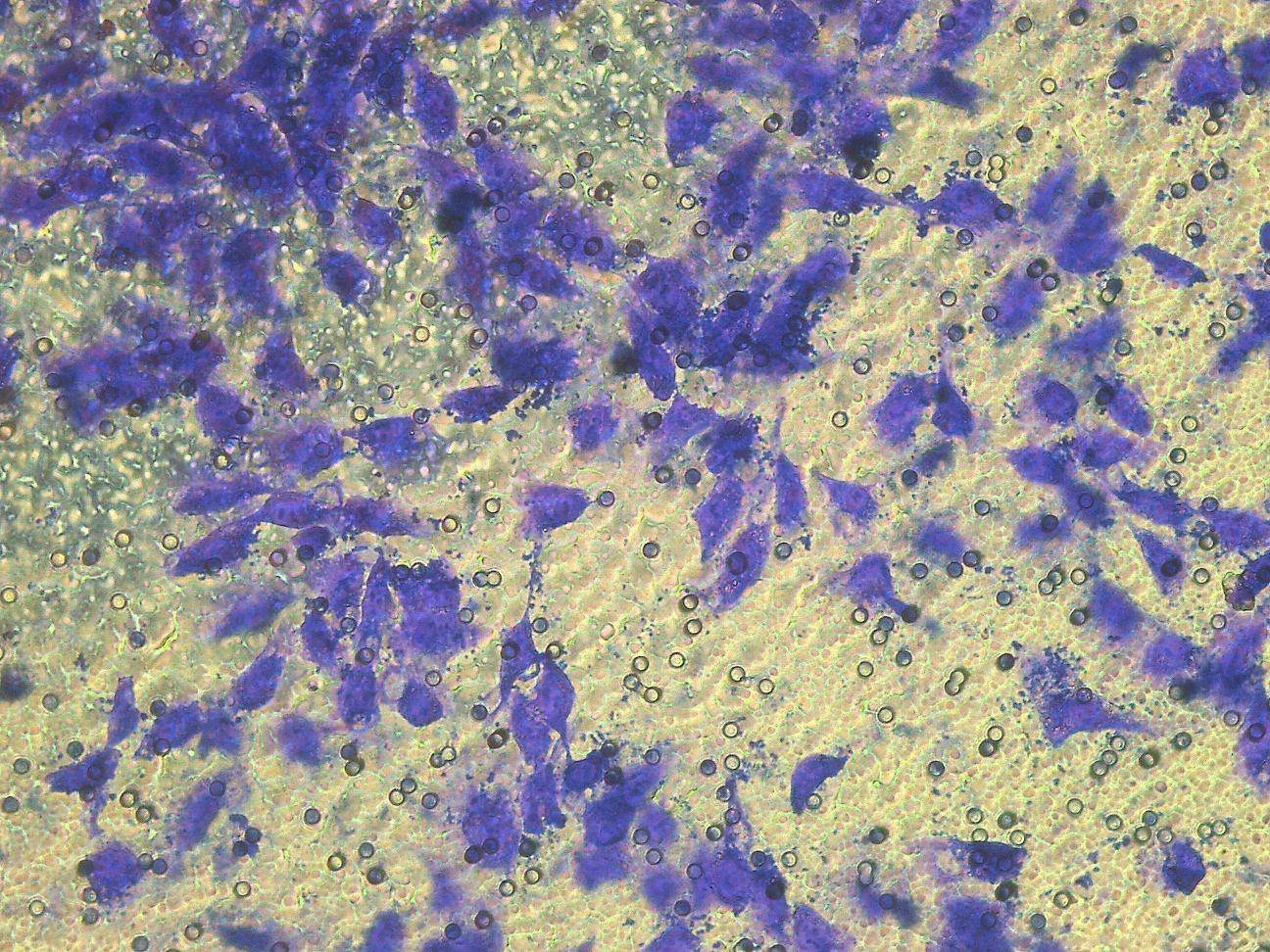

Supplement: Supplementary file 1 [file diagnostics-16-02250-s001.zip › Supplementary original images/Transwell assay original images/TPC-1 Vector (Repeat3).jpg]

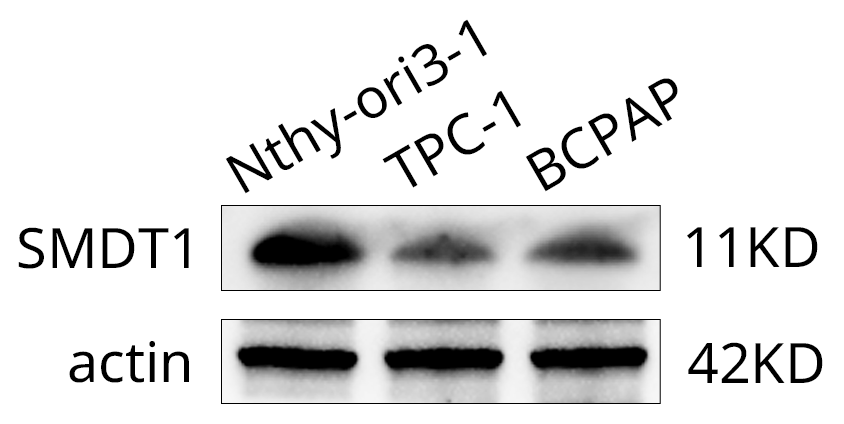

Supplement: Supplementary file 1 [file diagnostics-16-02250-s001.zip › Supplementary original images/Western blotting original images/Figure 7C.tif]

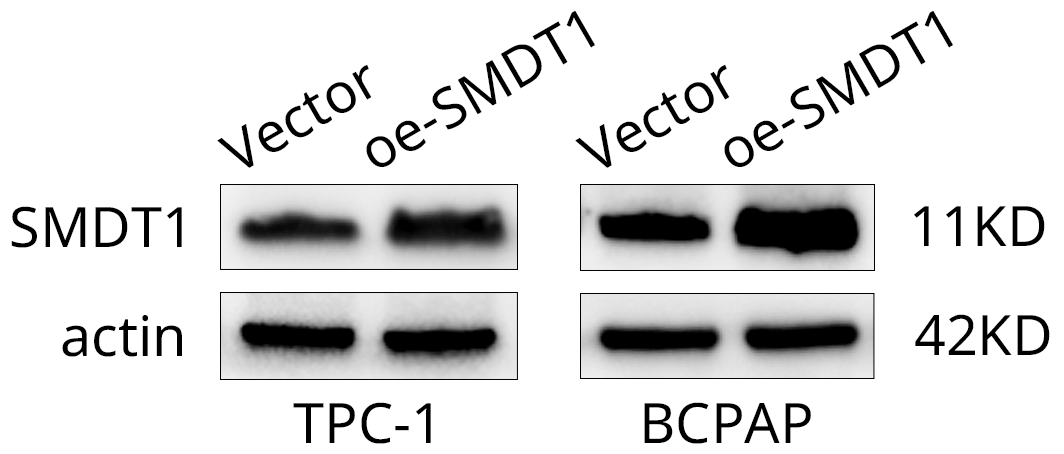

Supplement: Supplementary file 1 [file diagnostics-16-02250-s001.zip › Supplementary original images/Western blotting original images/Figure 7F.tif]

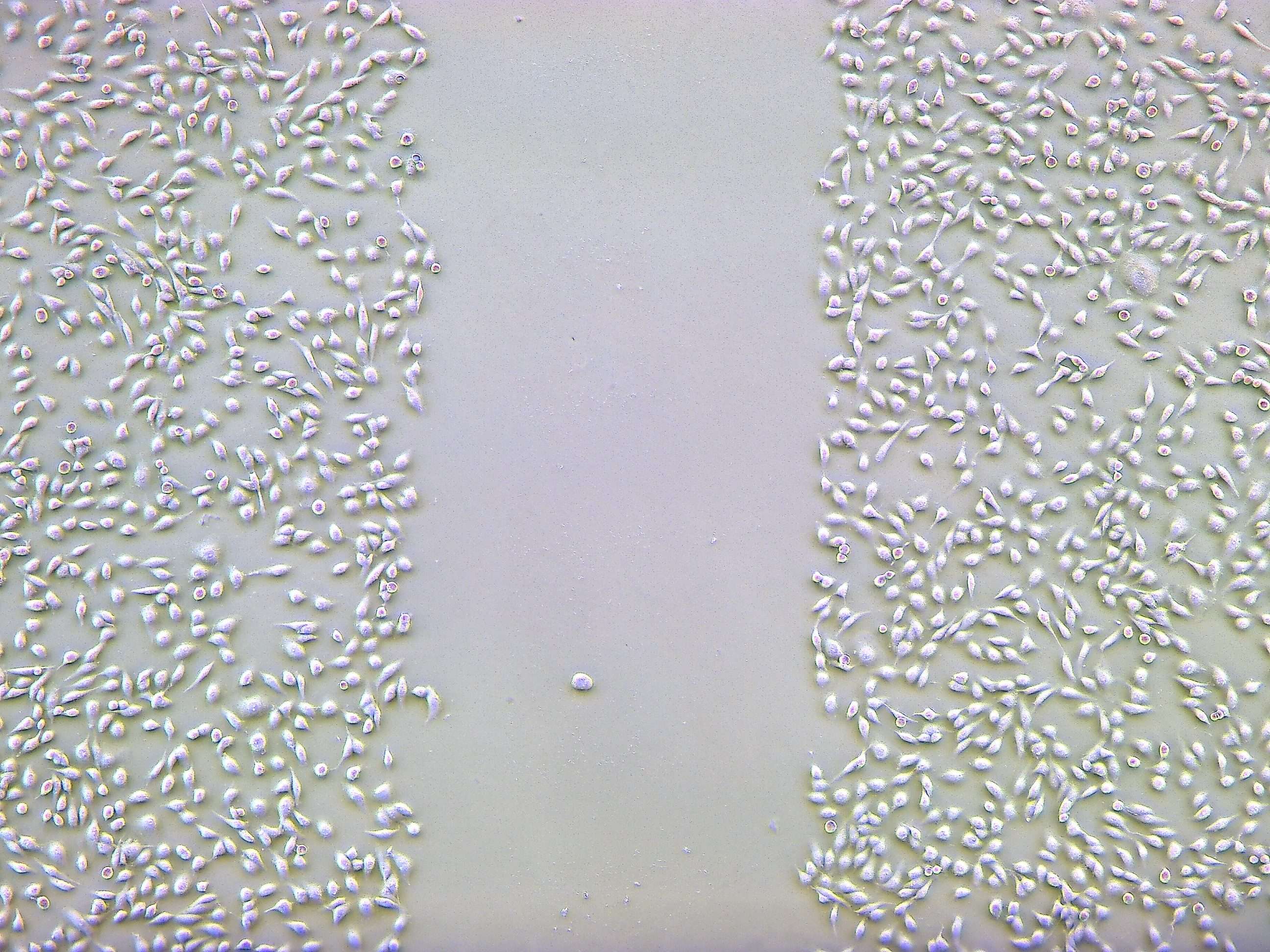

Supplement: Supplementary file 1 [file diagnostics-16-02250-s001.zip › Supplementary original images/Wound healing assay original images/BCPAP oe-SMDT1 0h (Repeat1).jpg]

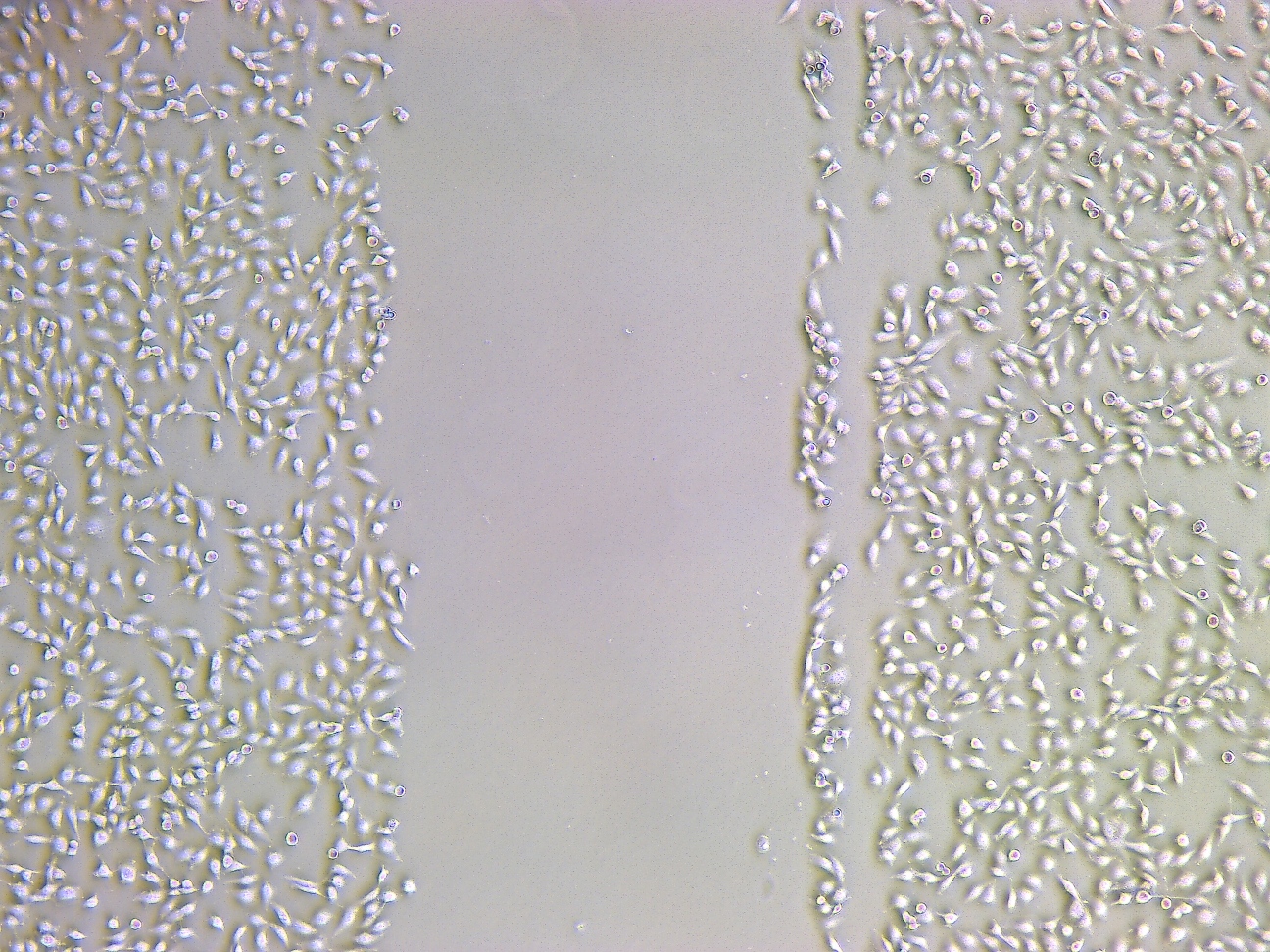

Supplement: Supplementary file 1 [file diagnostics-16-02250-s001.zip › Supplementary original images/Wound healing assay original images/BCPAP oe-SMDT1 0h (Repeat2).jpg]

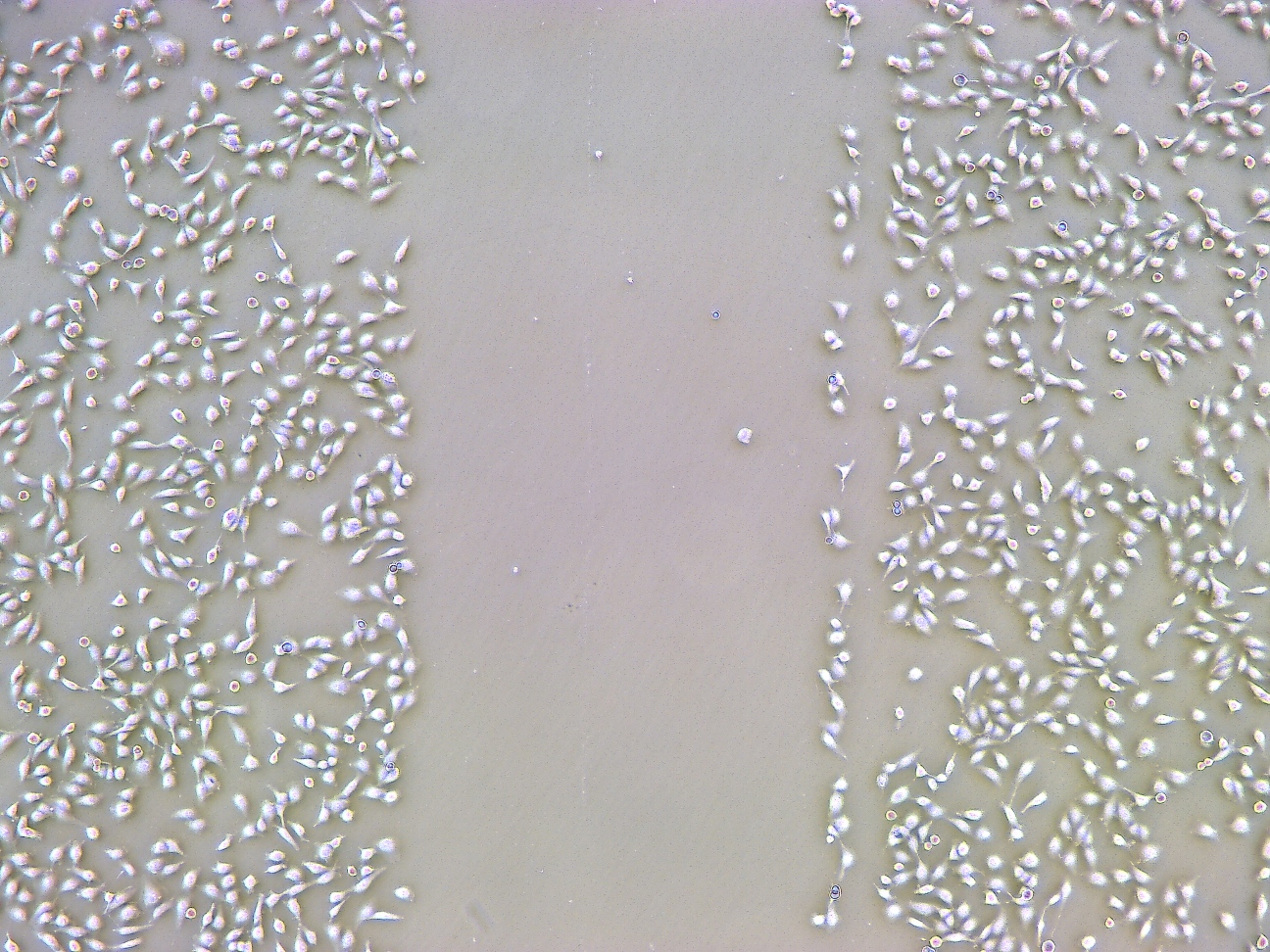

Supplement: Supplementary file 1 [file diagnostics-16-02250-s001.zip › Supplementary original images/Wound healing assay original images/BCPAP oe-SMDT1 0h (Repeat3).jpg]

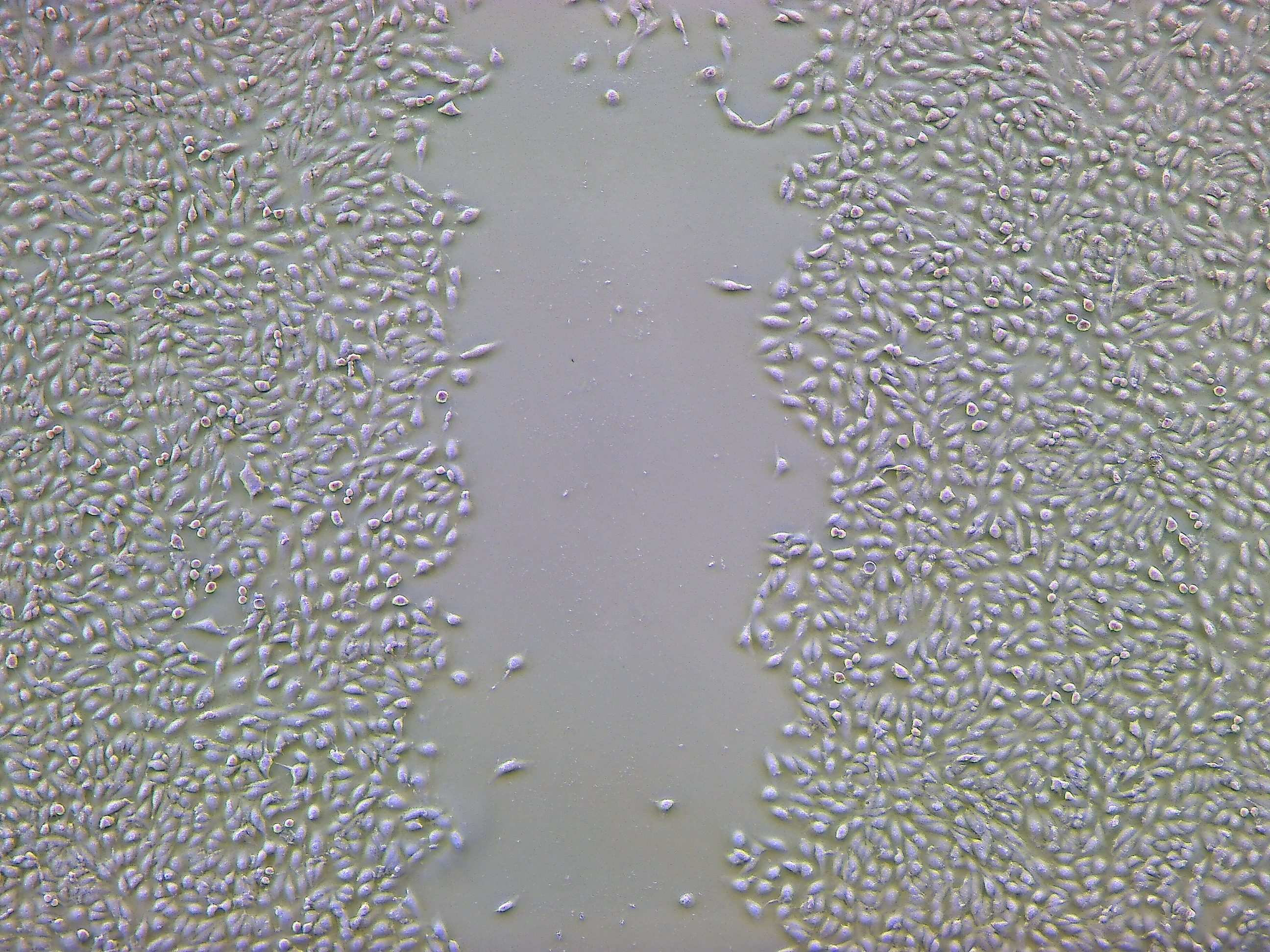

Supplement: Supplementary file 1 [file diagnostics-16-02250-s001.zip › Supplementary original images/Wound healing assay original images/BCPAP oe-SMDT1 24h (Repeat1).jpg]

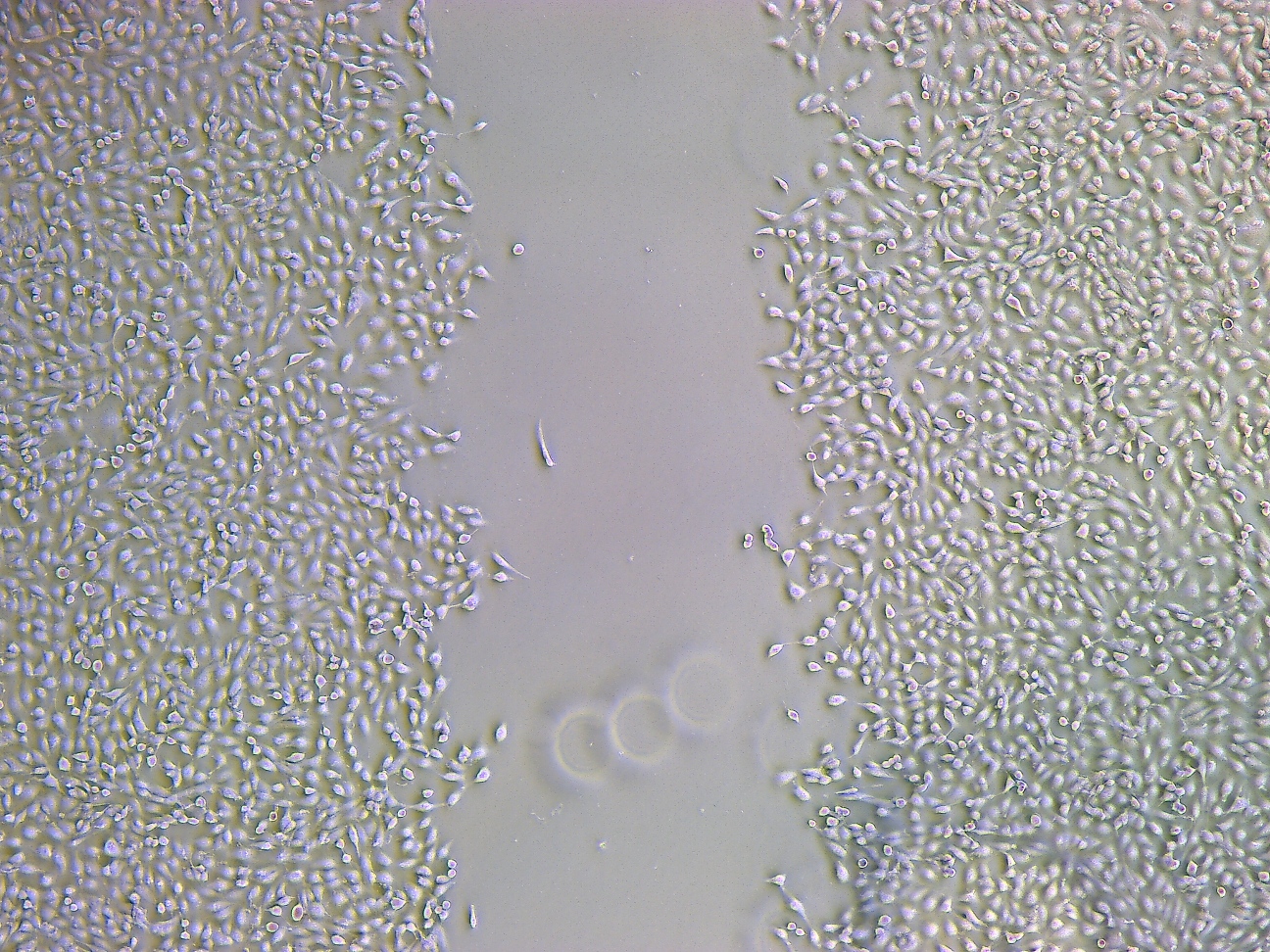

Supplement: Supplementary file 1 [file diagnostics-16-02250-s001.zip › Supplementary original images/Wound healing assay original images/BCPAP oe-SMDT1 24h (Repeat2).jpg]

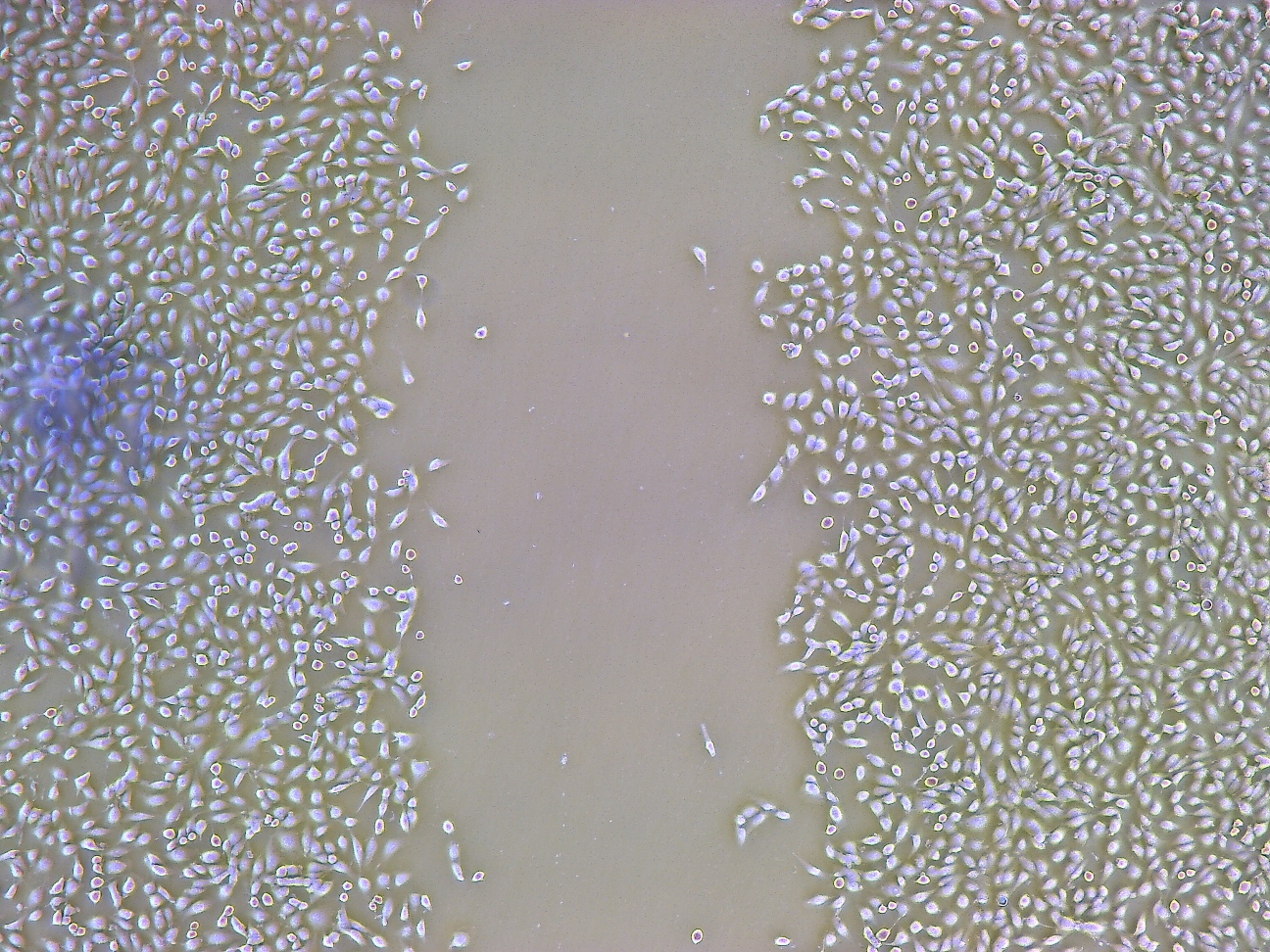

Supplement: Supplementary file 1 [file diagnostics-16-02250-s001.zip › Supplementary original images/Wound healing assay original images/BCPAP oe-SMDT1 24h (Repeat3).jpg]

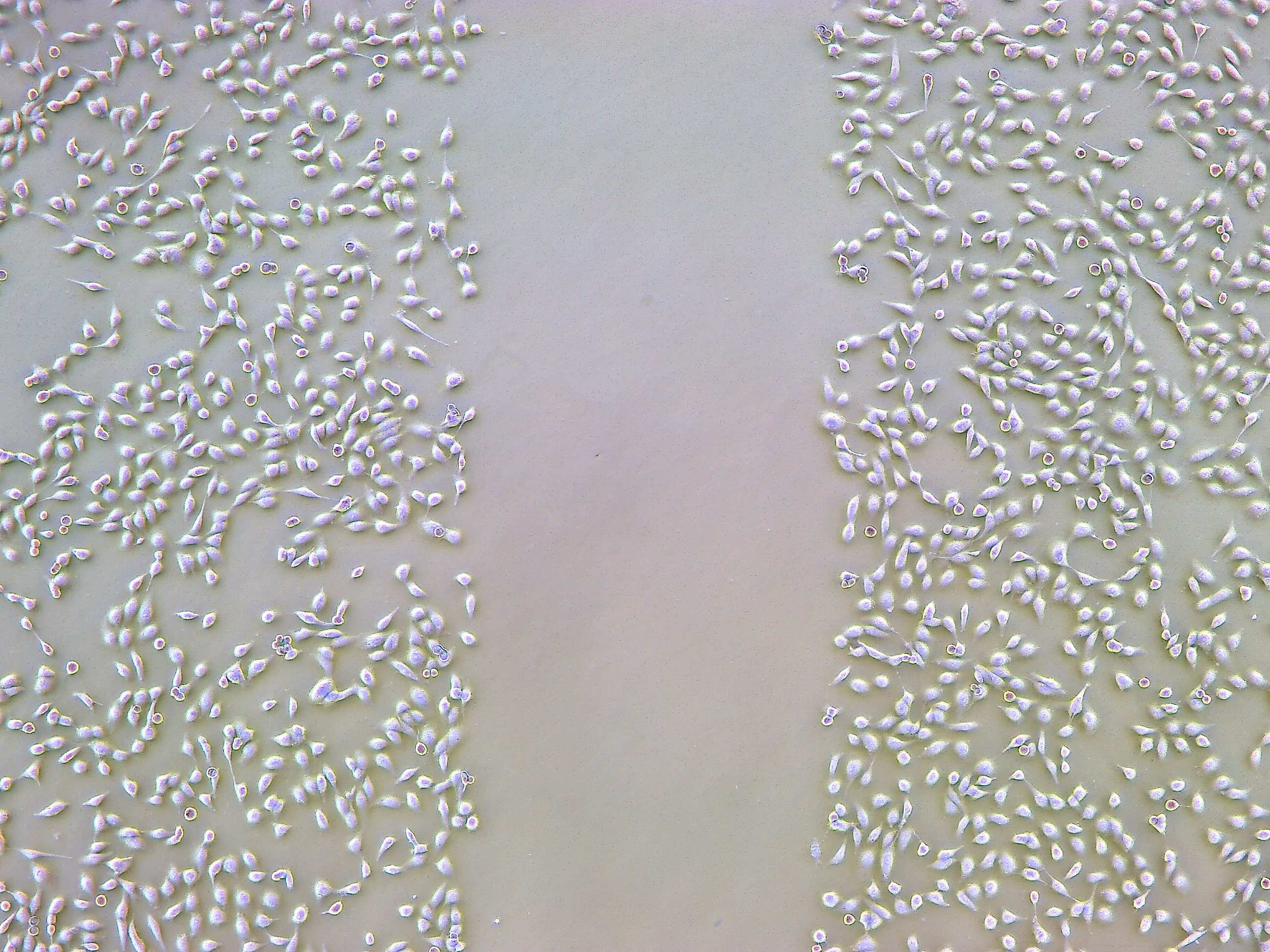

Supplement: Supplementary file 1 [file diagnostics-16-02250-s001.zip › Supplementary original images/Wound healing assay original images/BCPAP Vector 0h (Repeat1).jpg]

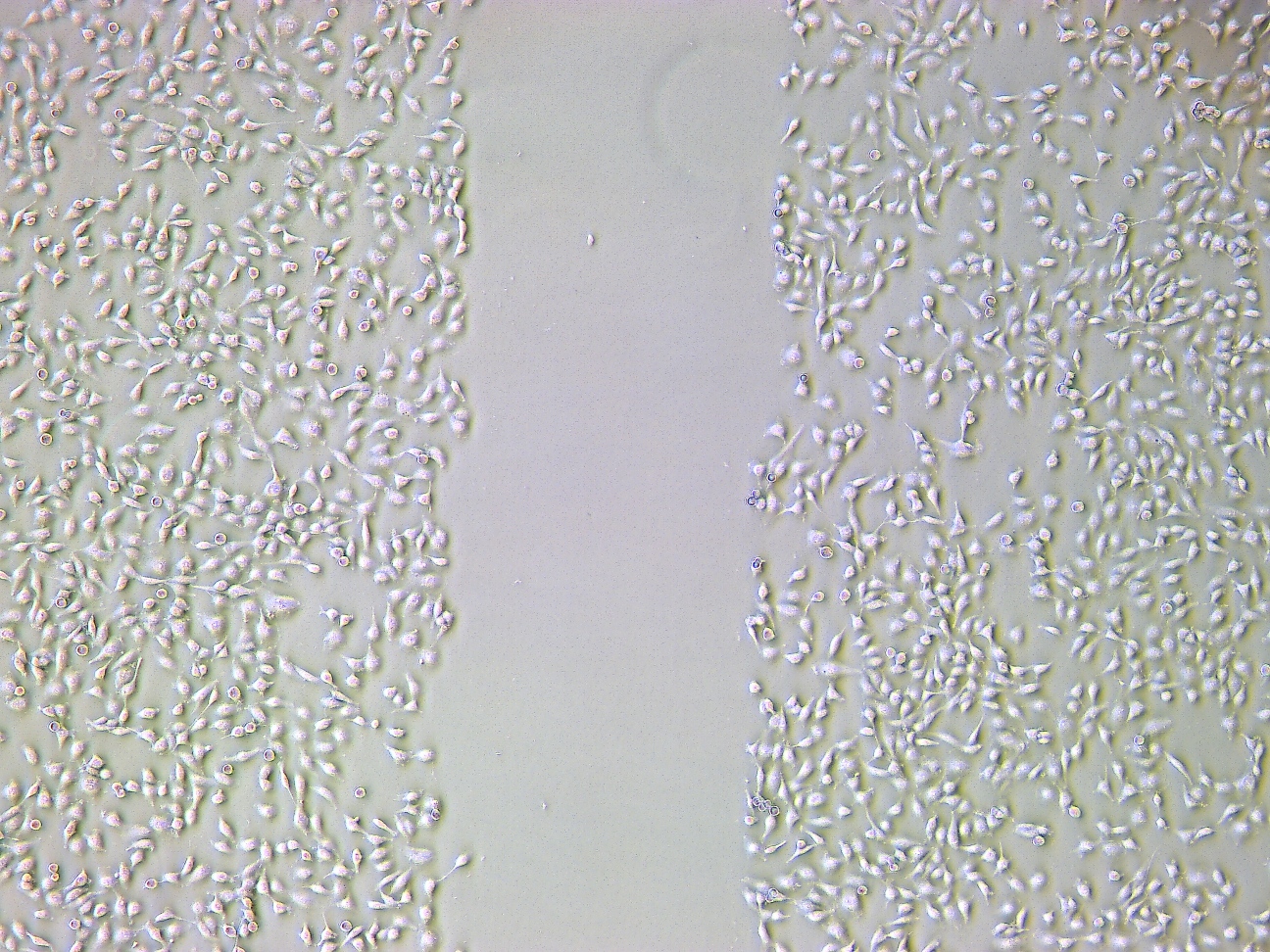

Supplement: Supplementary file 1 [file diagnostics-16-02250-s001.zip › Supplementary original images/Wound healing assay original images/BCPAP Vector 0h (Repeat2).jpg]

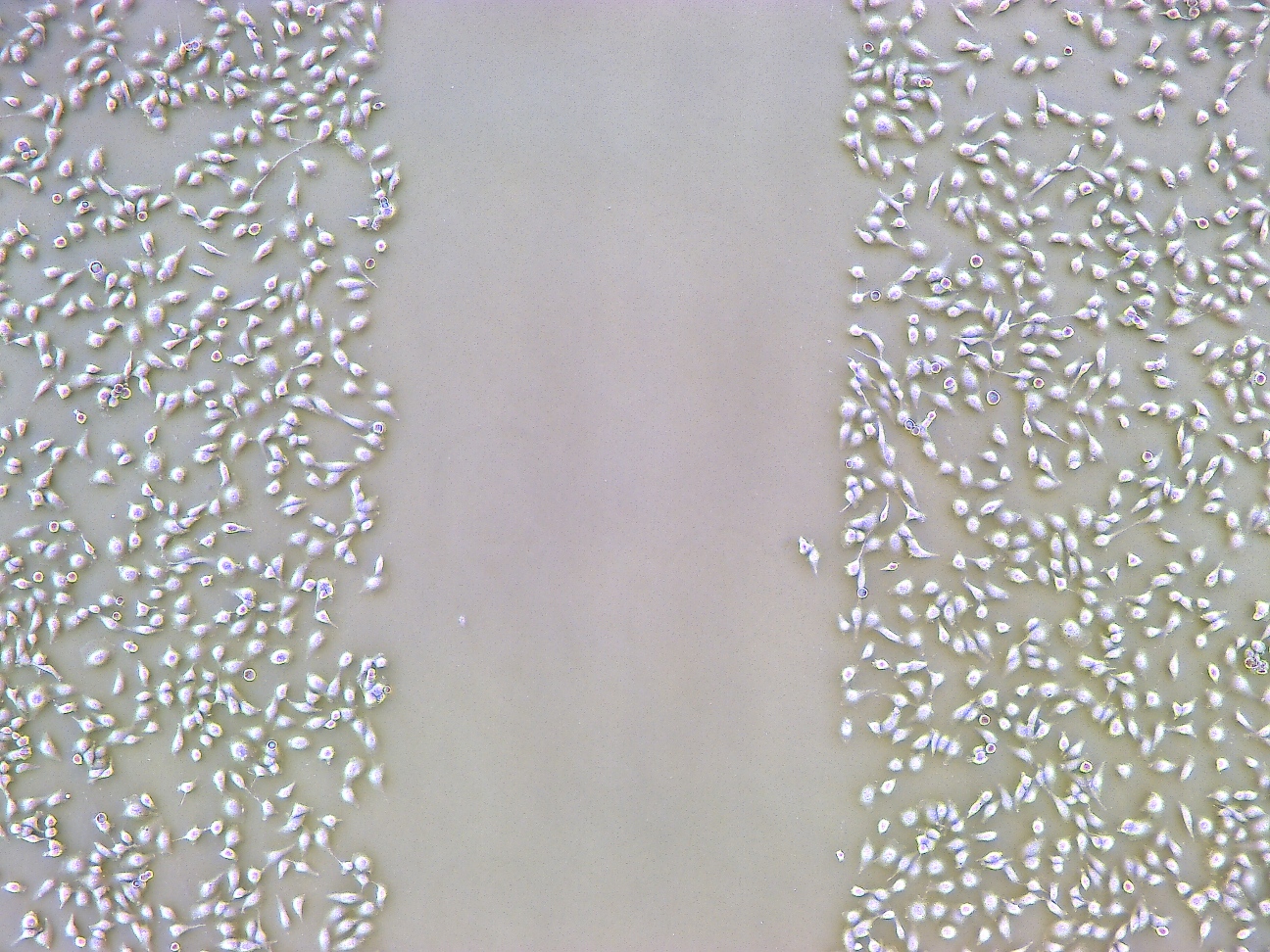

Supplement: Supplementary file 1 [file diagnostics-16-02250-s001.zip › Supplementary original images/Wound healing assay original images/BCPAP Vector 0h (Repeat3).jpg]

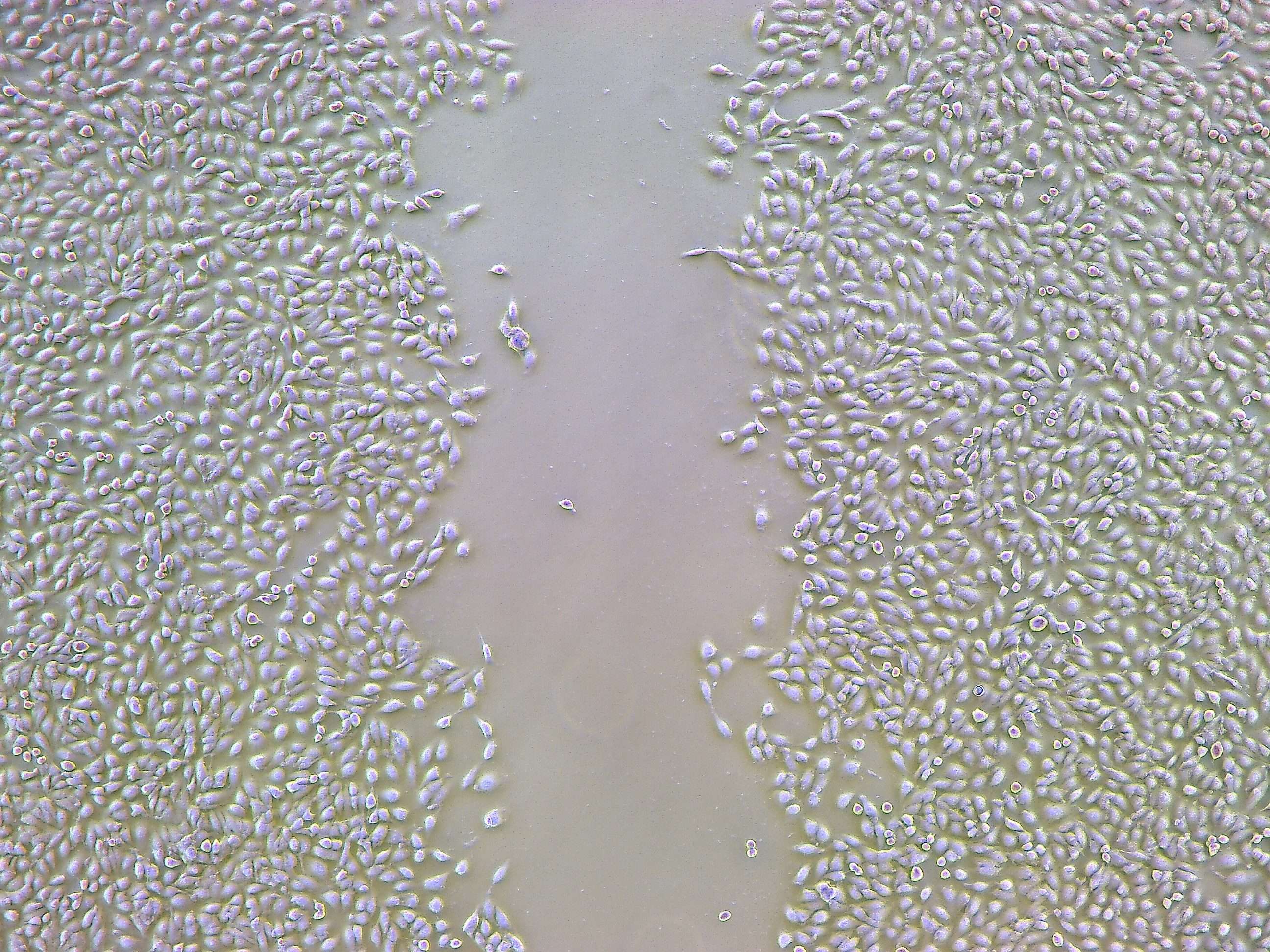

Supplement: Supplementary file 1 [file diagnostics-16-02250-s001.zip › Supplementary original images/Wound healing assay original images/BCPAP Vector 24h (Repeat1).jpg]

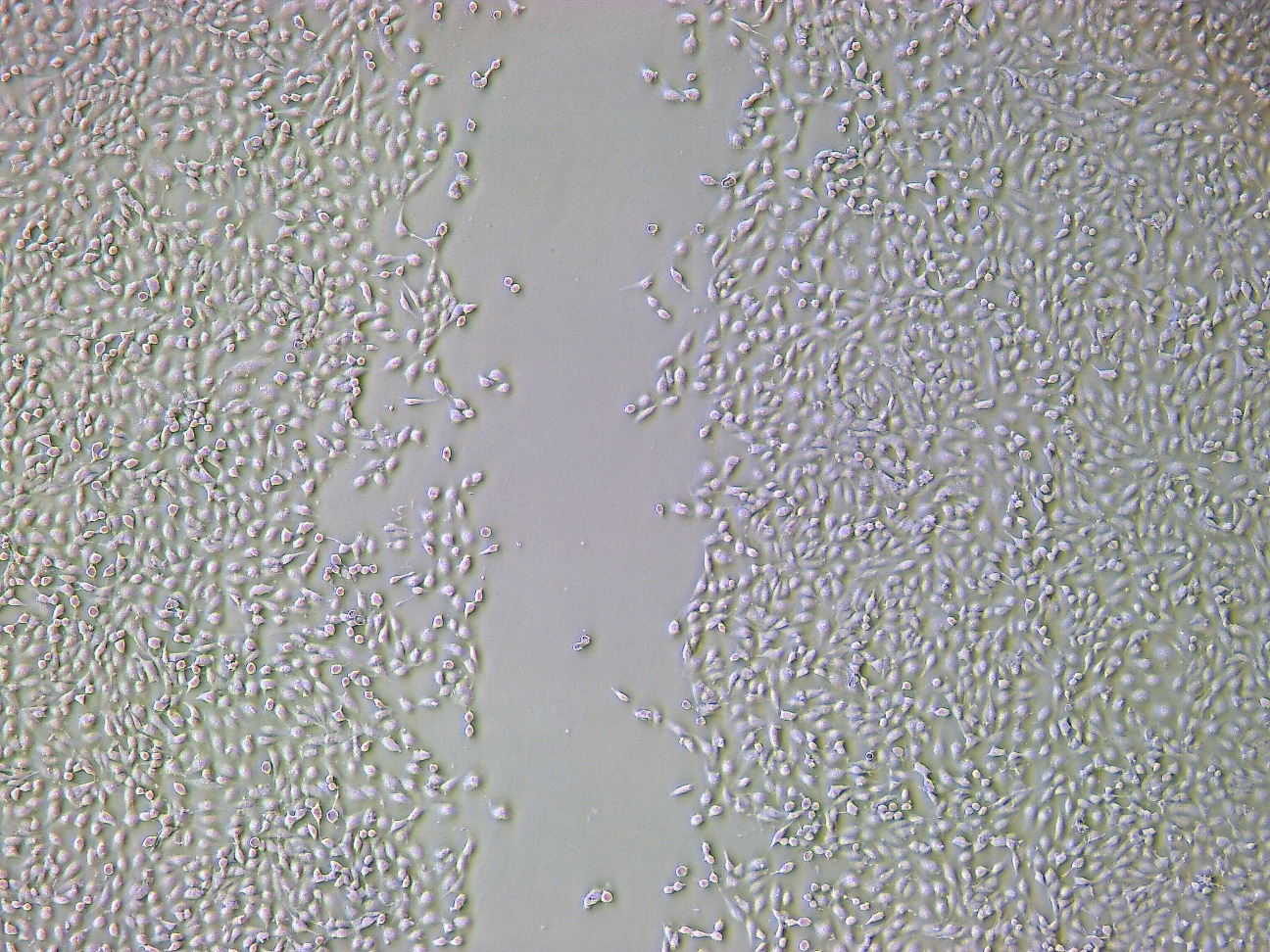

Supplement: Supplementary file 1 [file diagnostics-16-02250-s001.zip › Supplementary original images/Wound healing assay original images/BCPAP Vector 24h (Repeat2).jpg]

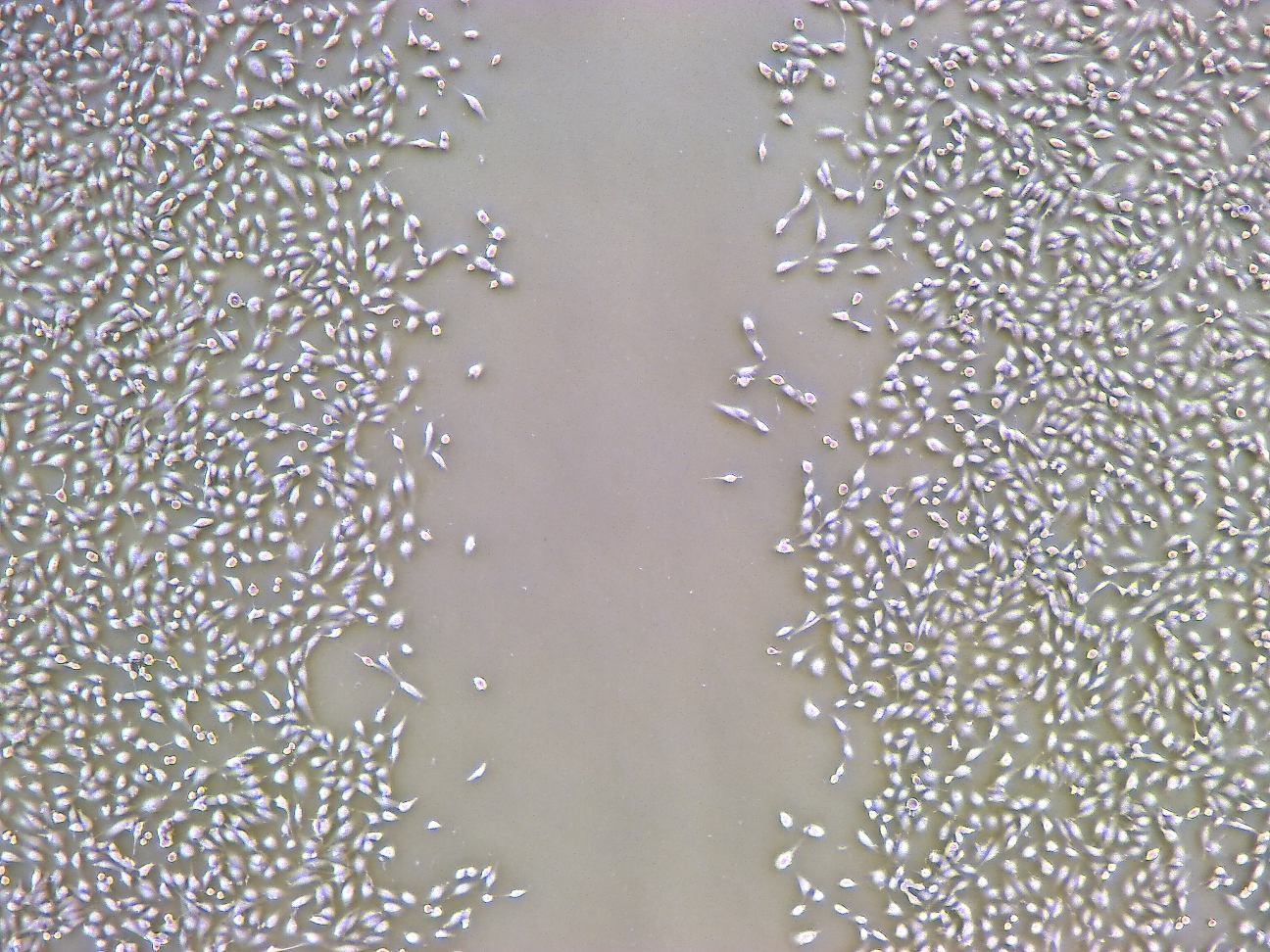

Supplement: Supplementary file 1 [file diagnostics-16-02250-s001.zip › Supplementary original images/Wound healing assay original images/BCPAP Vector 24h (Repeat3).jpg]

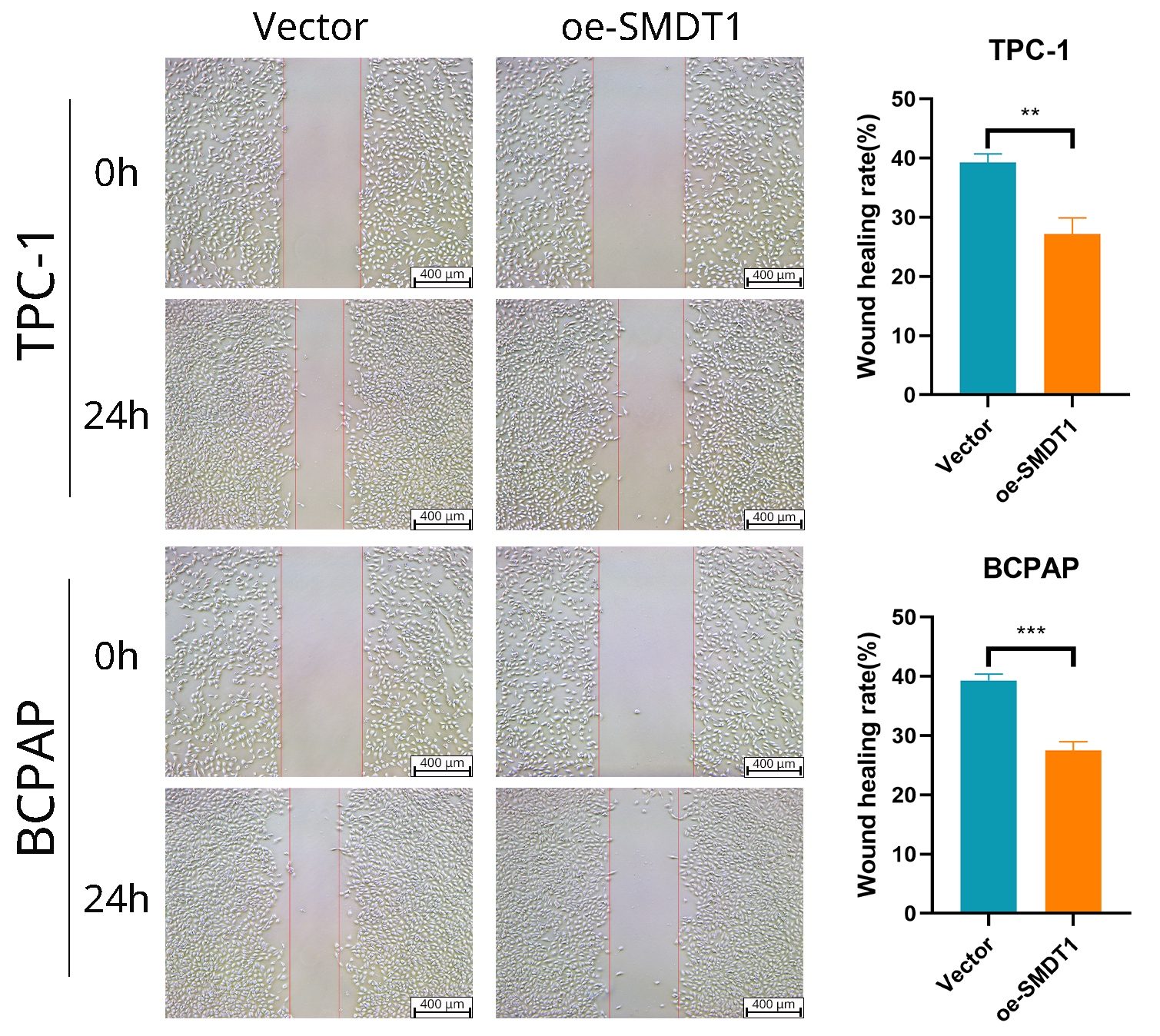

Supplement: Supplementary file 1 [file diagnostics-16-02250-s001.zip › Supplementary original images/Wound healing assay original images/Figure 8C.jpg]

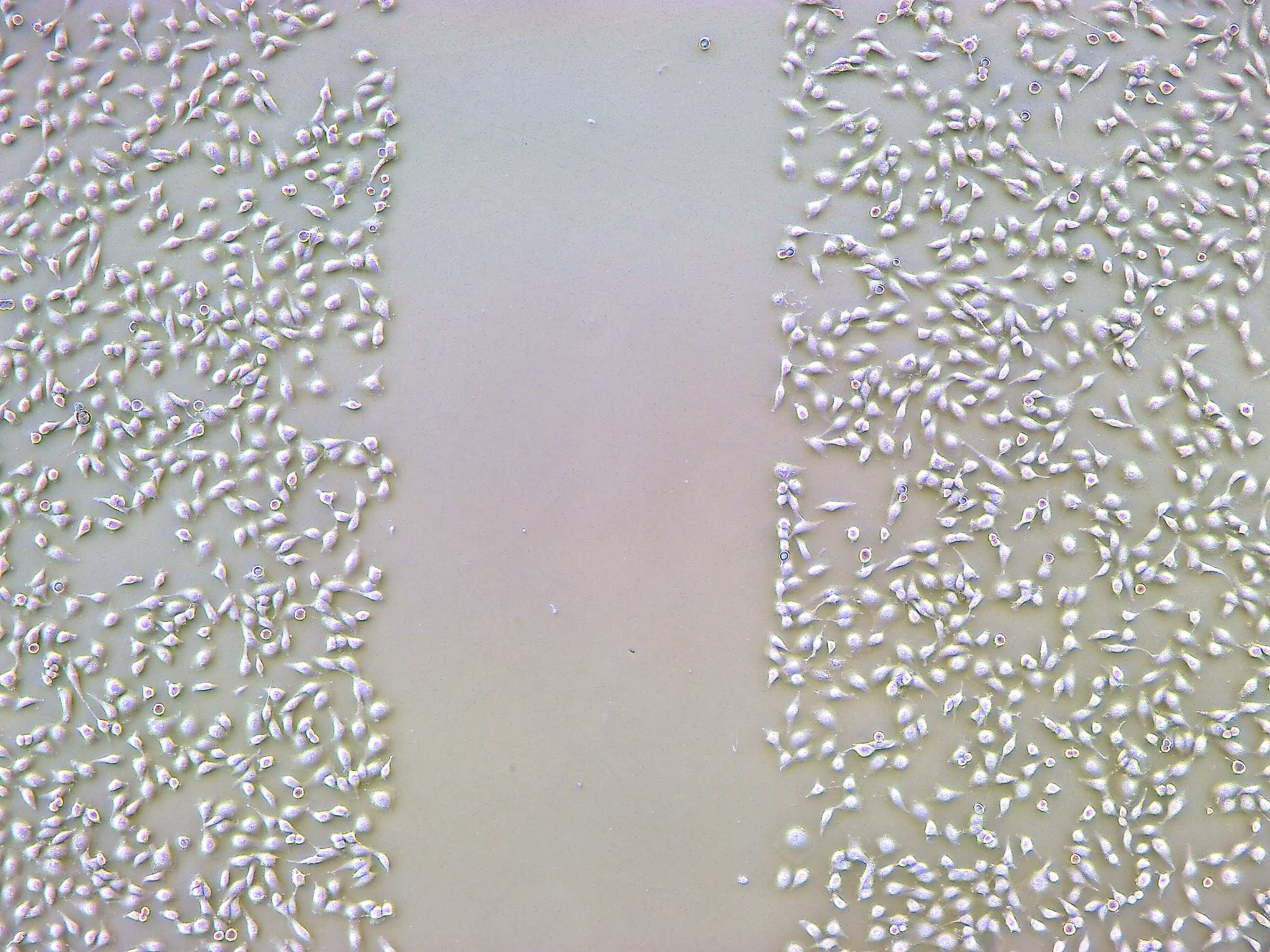

Supplement: Supplementary file 1 [file diagnostics-16-02250-s001.zip › Supplementary original images/Wound healing assay original images/TPC-1 oe-SMDT1 0h (Repeat1).jpg]

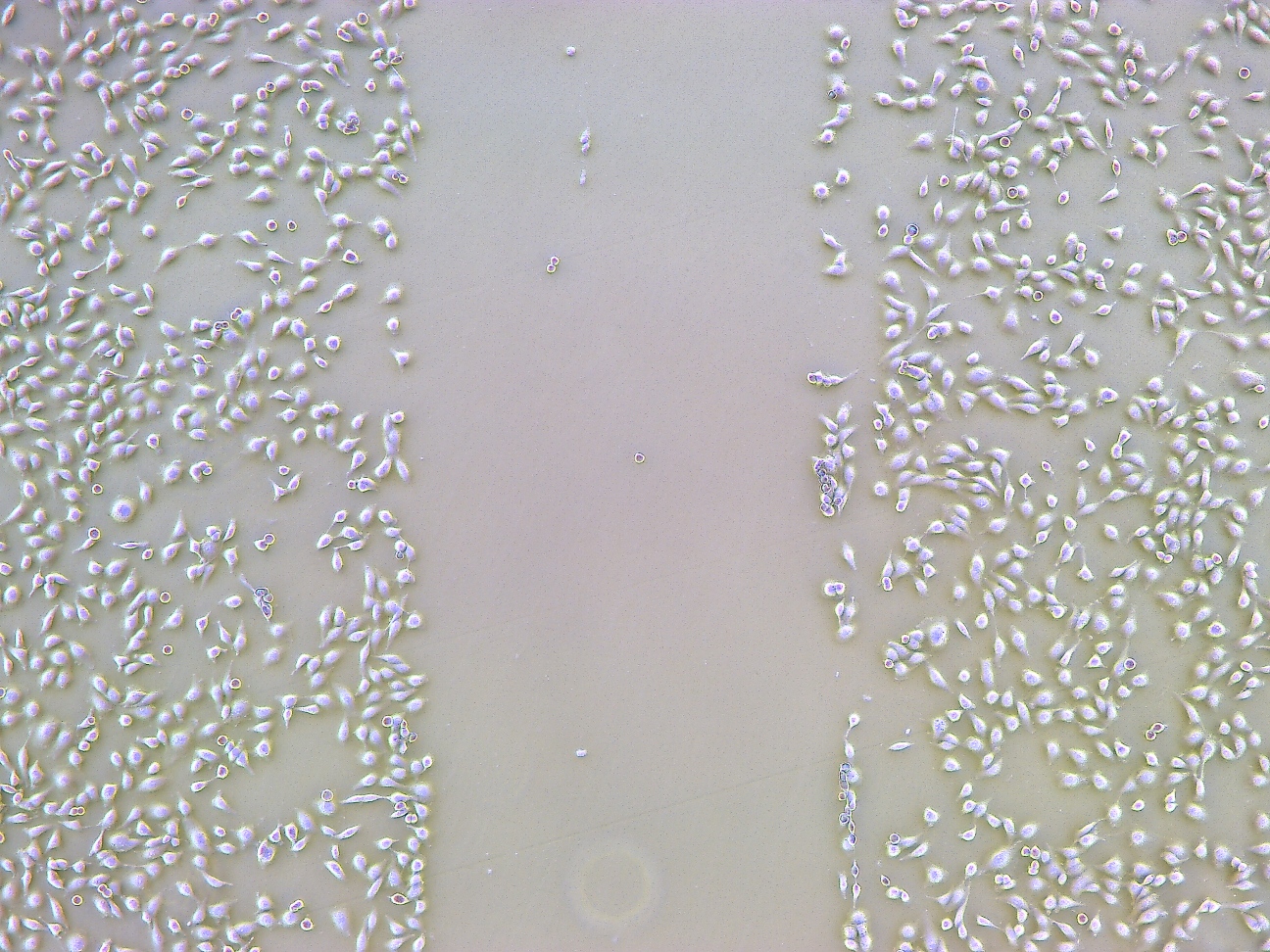

Supplement: Supplementary file 1 [file diagnostics-16-02250-s001.zip › Supplementary original images/Wound healing assay original images/TPC-1 oe-SMDT1 0h (Repeat2).jpg]

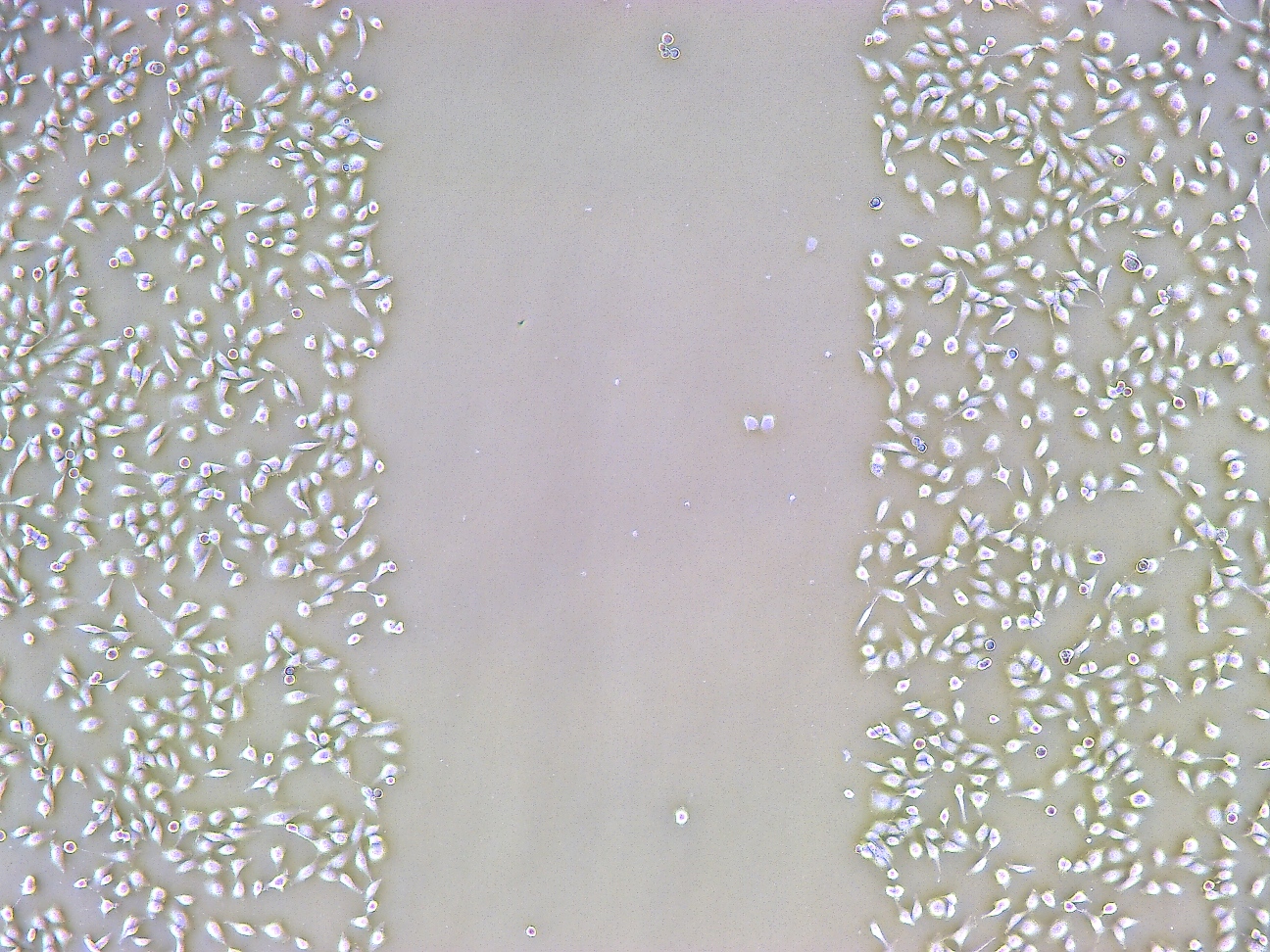

Supplement: Supplementary file 1 [file diagnostics-16-02250-s001.zip › Supplementary original images/Wound healing assay original images/TPC-1 oe-SMDT1 0h (Repeat3).jpg]

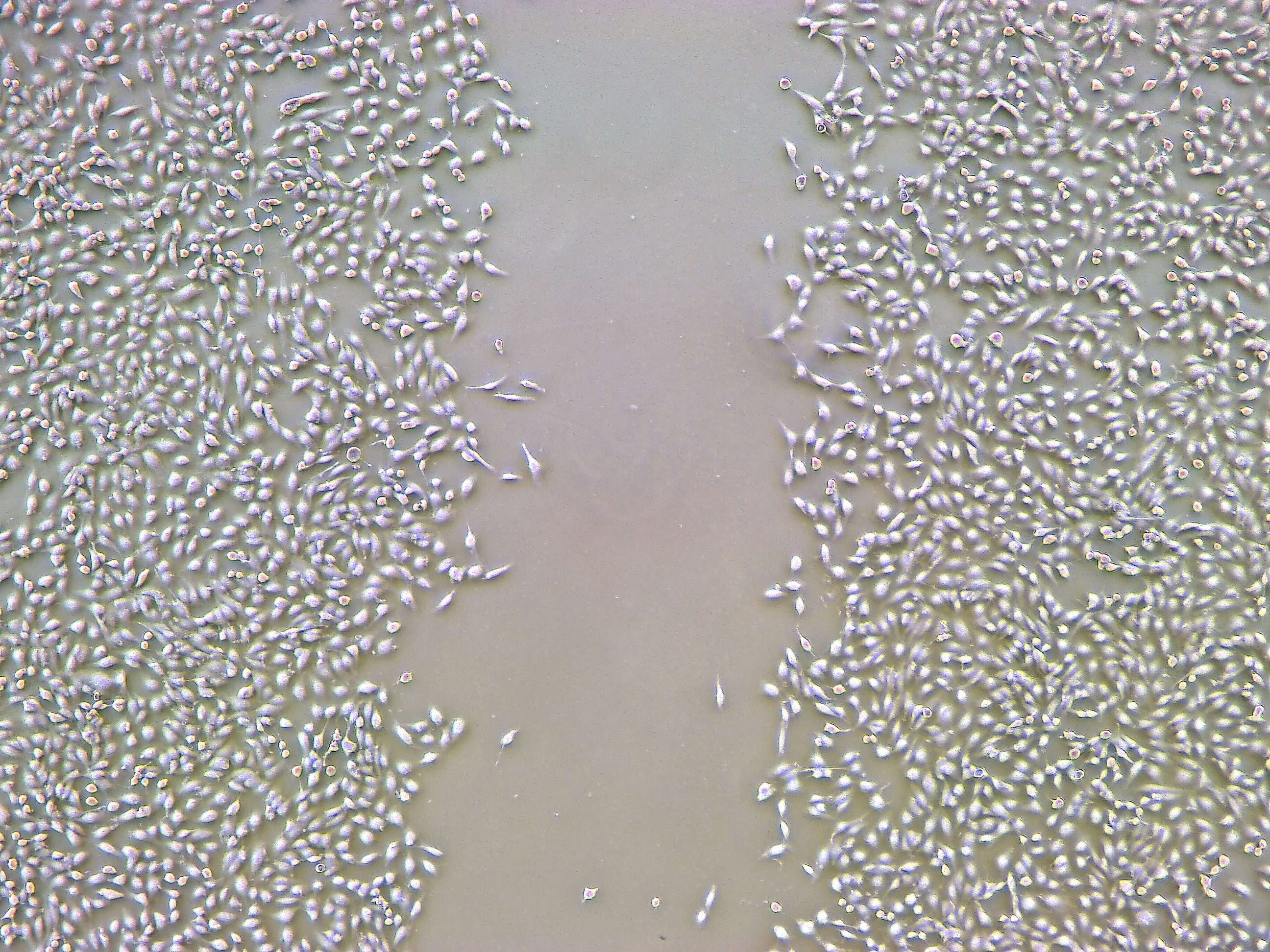

Supplement: Supplementary file 1 [file diagnostics-16-02250-s001.zip › Supplementary original images/Wound healing assay original images/TPC-1 oe-SMDT1 24h (Repeat1).jpg]

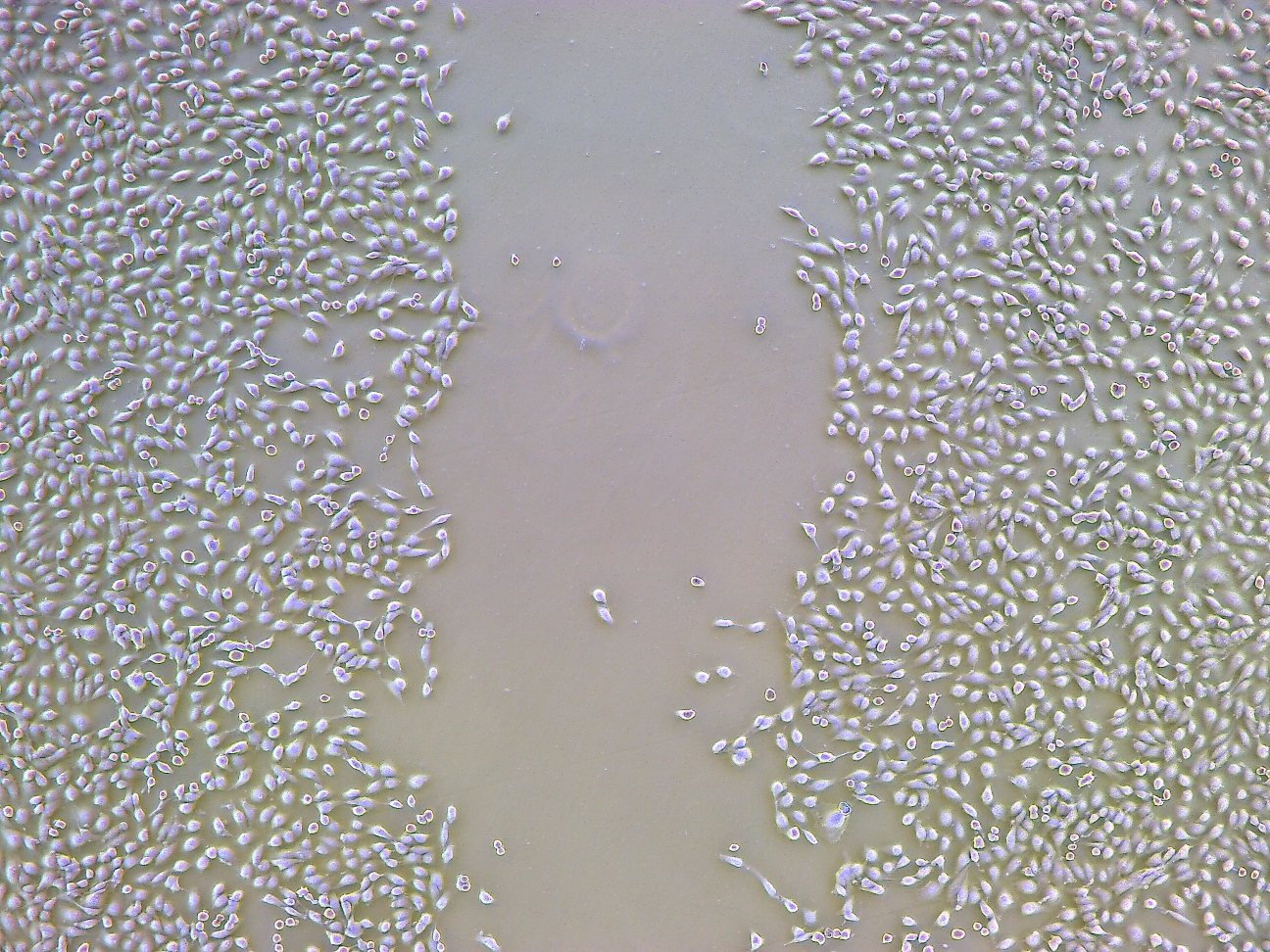

Supplement: Supplementary file 1 [file diagnostics-16-02250-s001.zip › Supplementary original images/Wound healing assay original images/TPC-1 oe-SMDT1 24h (Repeat2).jpg]

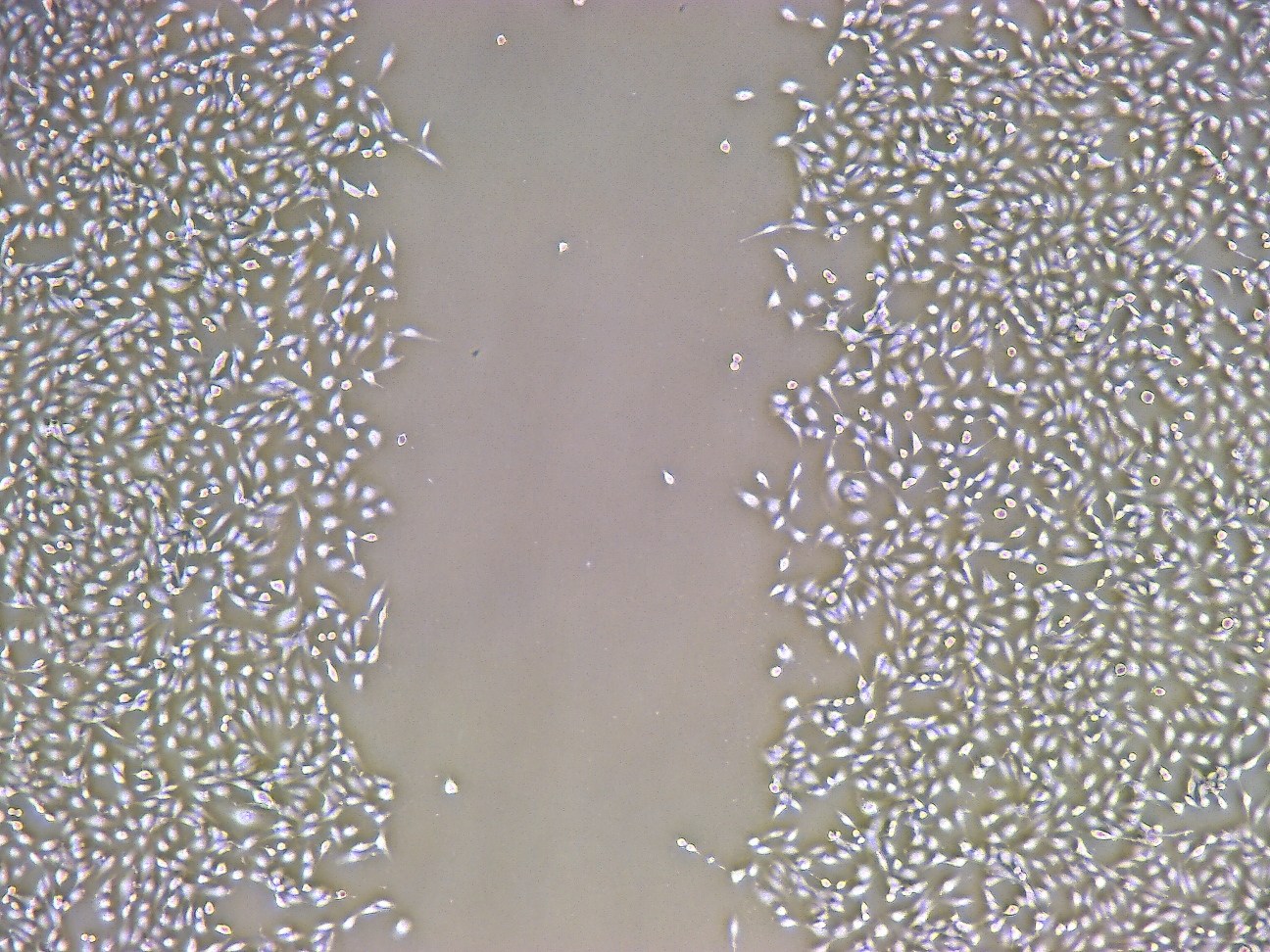

Supplement: Supplementary file 1 [file diagnostics-16-02250-s001.zip › Supplementary original images/Wound healing assay original images/TPC-1 oe-SMDT1 24h (Repeat3).jpg]

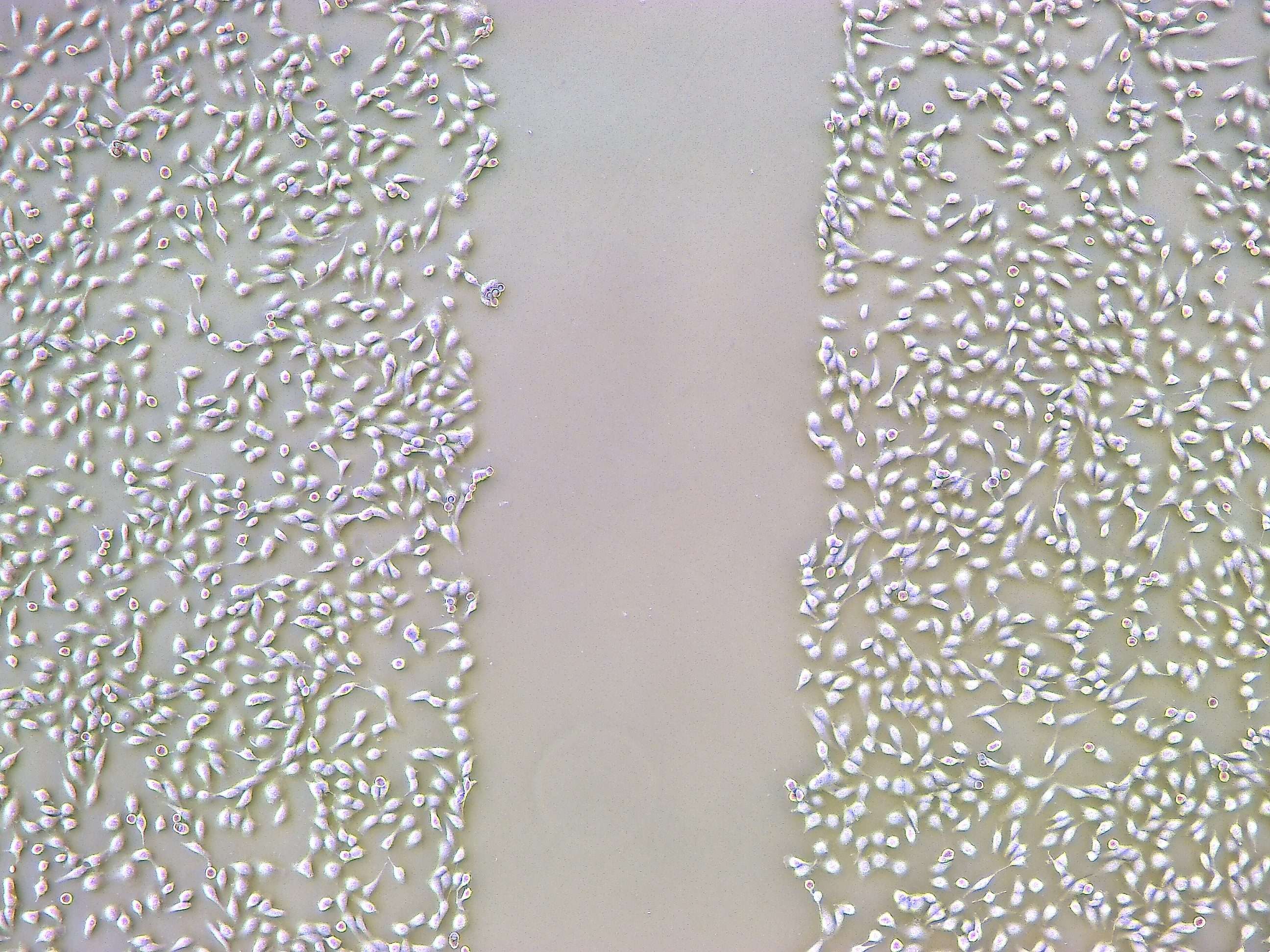

Supplement: Supplementary file 1 [file diagnostics-16-02250-s001.zip › Supplementary original images/Wound healing assay original images/TPC-1 Vector 0h (Repeat1).jpg]

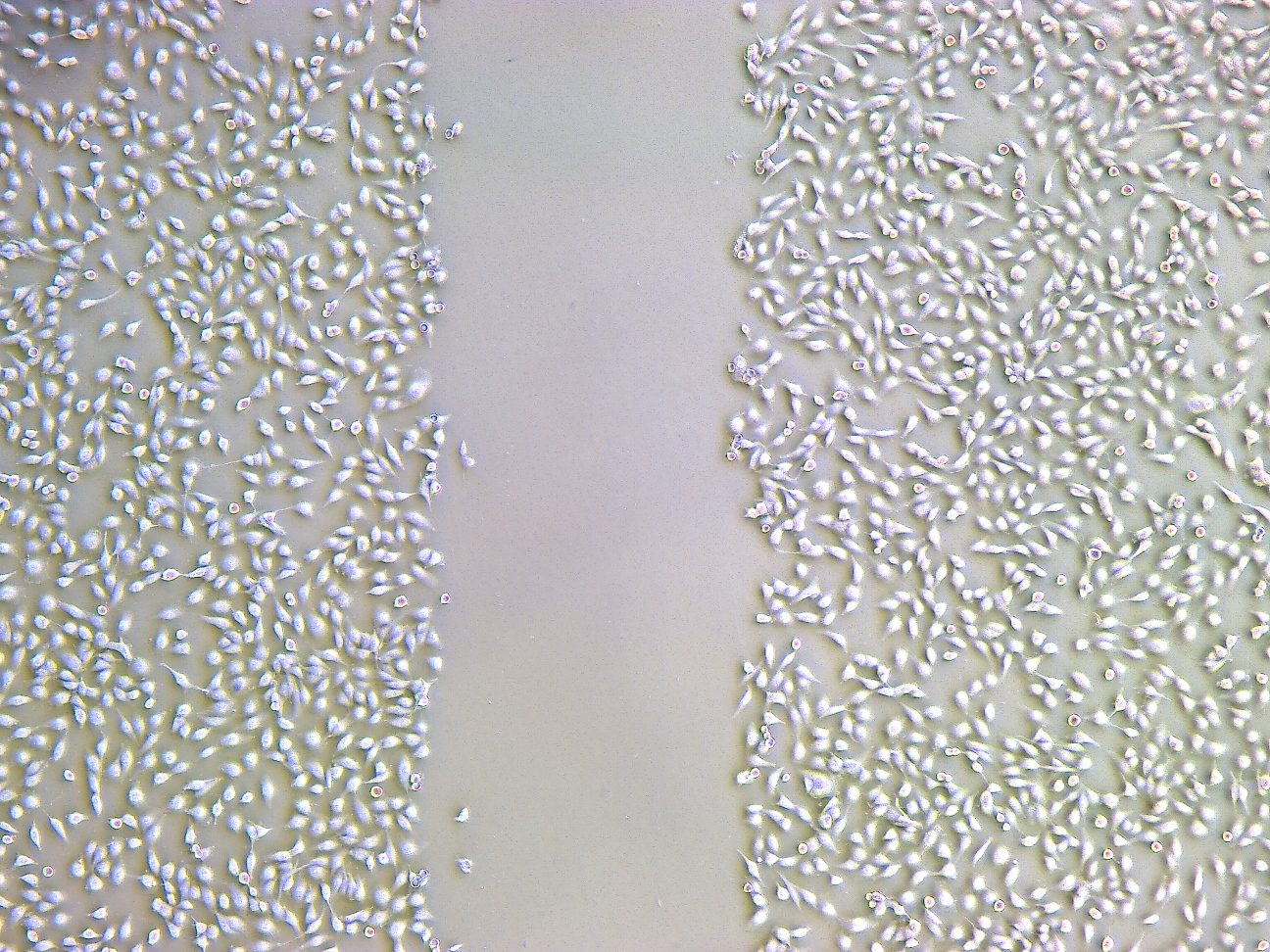

Supplement: Supplementary file 1 [file diagnostics-16-02250-s001.zip › Supplementary original images/Wound healing assay original images/TPC-1 Vector 0h (Repeat2).jpg]

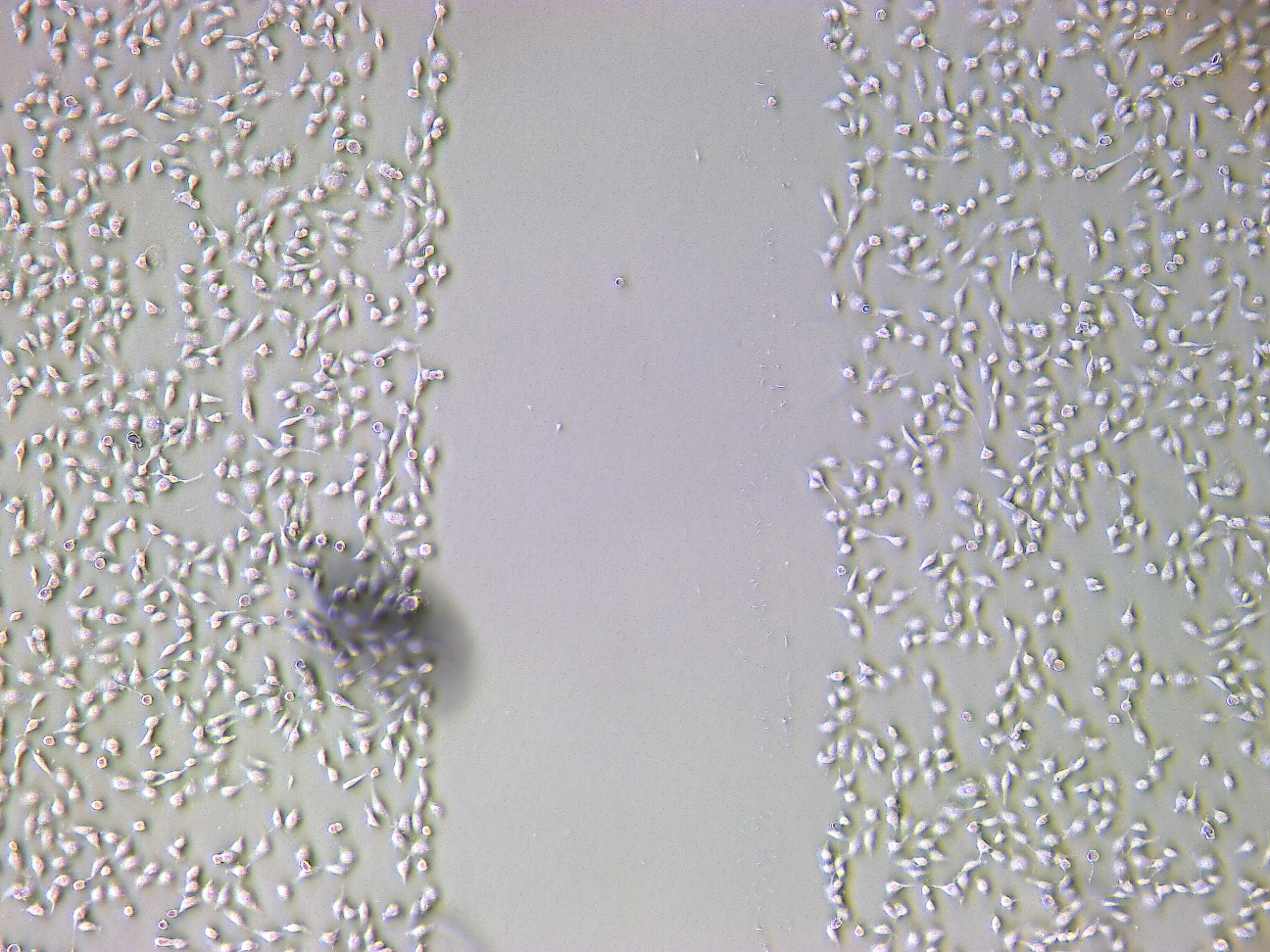

Supplement: Supplementary file 1 [file diagnostics-16-02250-s001.zip › Supplementary original images/Wound healing assay original images/TPC-1 Vector 0h (Repeat3).jpg]

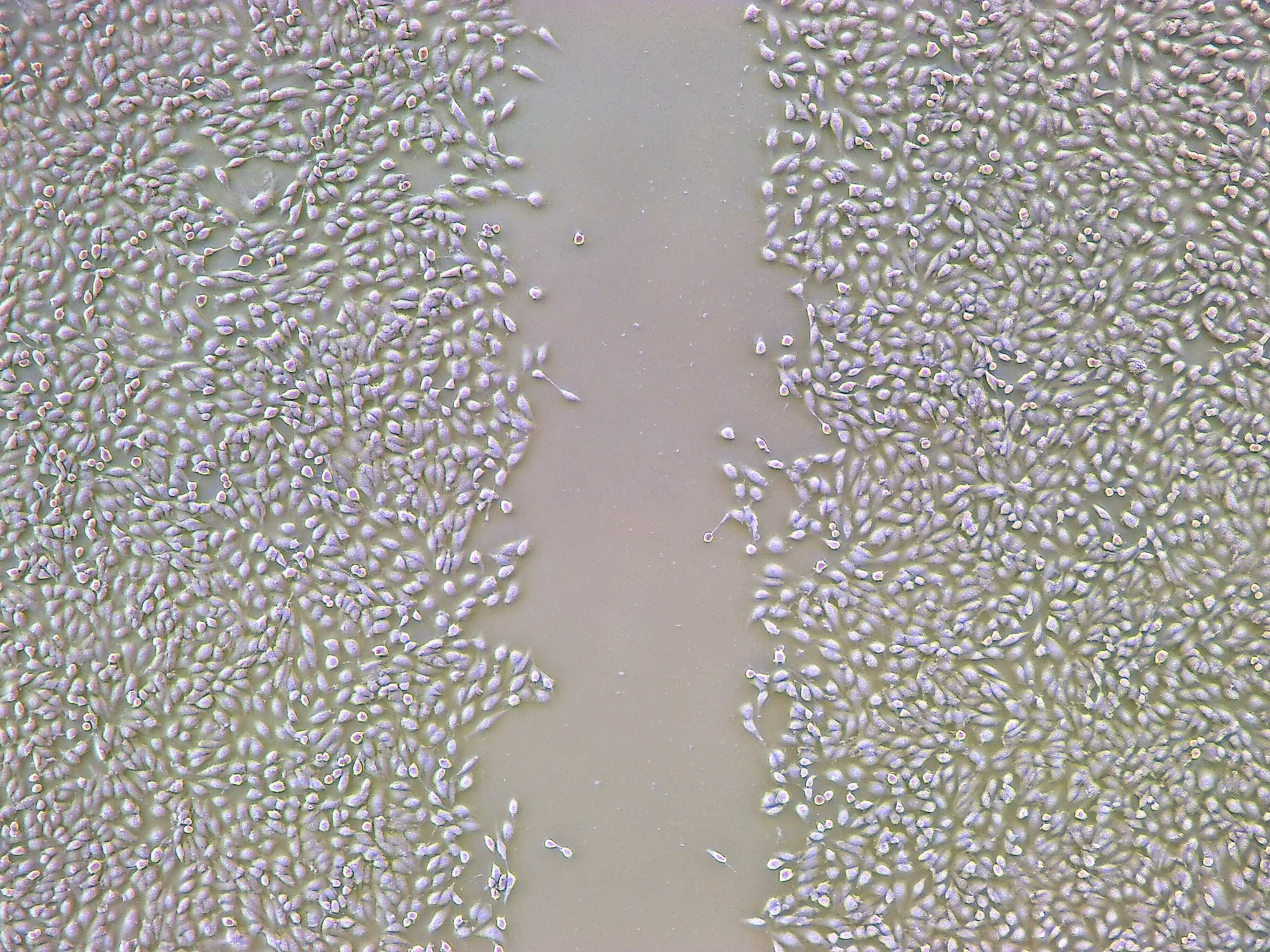

Supplement: Supplementary file 1 [file diagnostics-16-02250-s001.zip › Supplementary original images/Wound healing assay original images/TPC-1 Vector 24h (Repeat1).jpg]

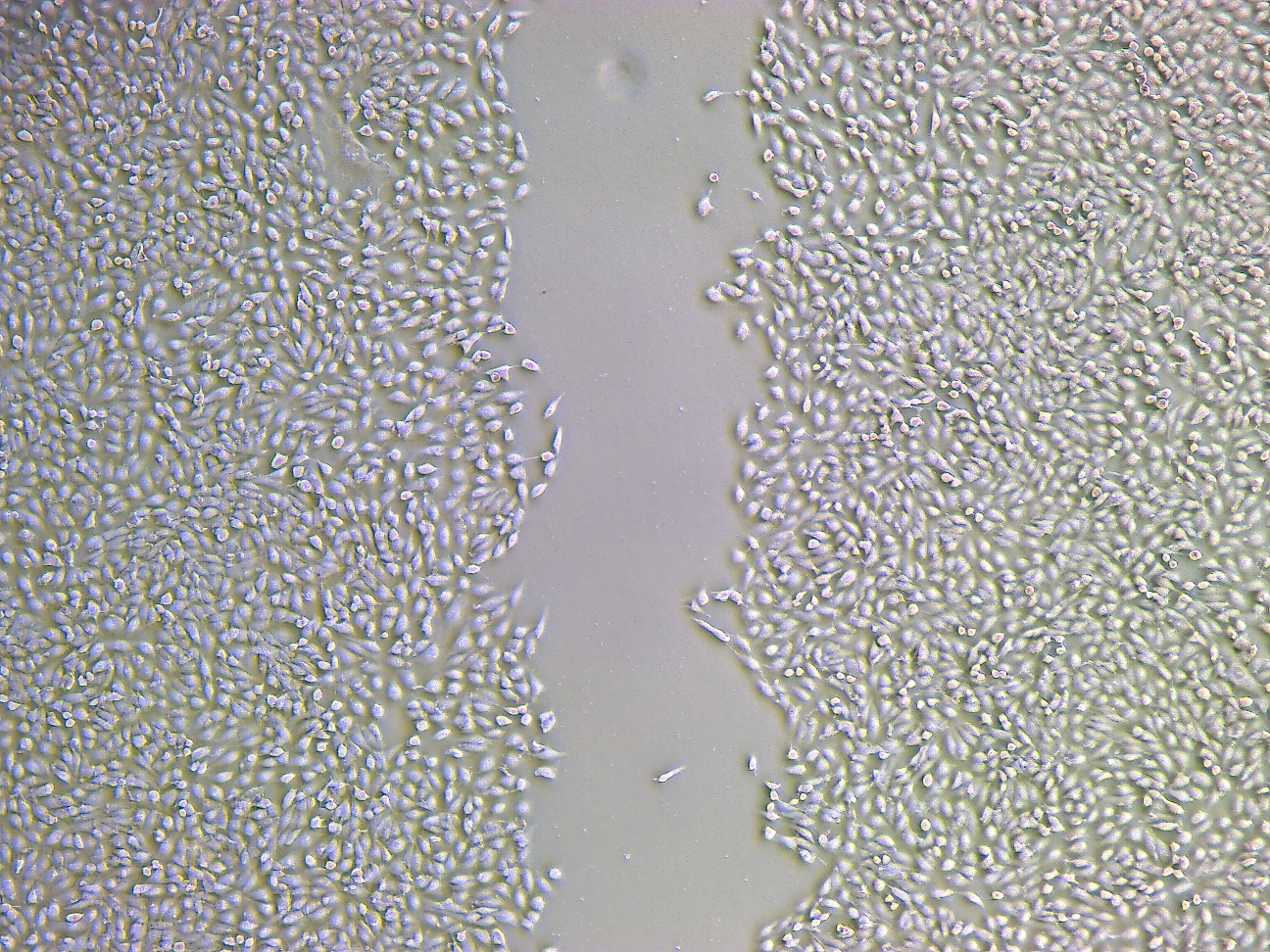

Supplement: Supplementary file 1 [file diagnostics-16-02250-s001.zip › Supplementary original images/Wound healing assay original images/TPC-1 Vector 24h (Repeat2).jpg]

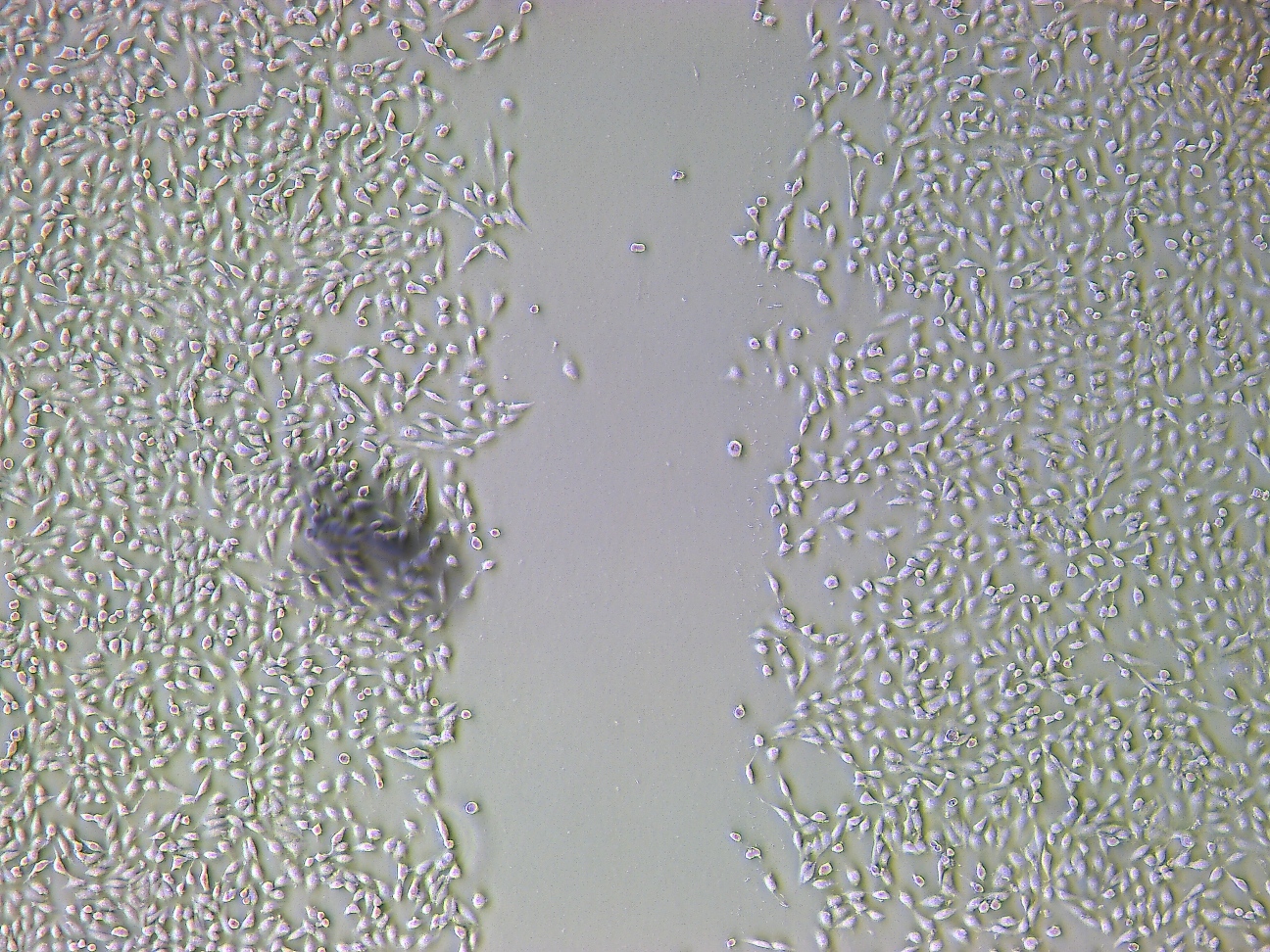

Supplement: Supplementary file 1 [file diagnostics-16-02250-s001.zip › Supplementary original images/Wound healing assay original images/TPC-1 Vector 24h (Repeat3).jpg]
